# GSK3 inhibition circumvents and overcomes acquired lorlatinib resistance in ALK-rearranged non-small-cell lung cancer

**DOI:** 10.1038/s41698-022-00260-0

**Published:** 2022-03-17

**Authors:** Yuki Shimizu, Koutaroh Okada, Jun Adachi, Yuichi Abe, Ryohei Narumi, Ken Uchibori, Noriko Yanagitani, Sumie Koike, Satoshi Takagi, Makoto Nishio, Naoya Fujita, Ryohei Katayama

**Affiliations:** 1 Division of Experimental Chemotherapy, Cancer Chemotherapy Center, Japanese Foundation for Cancer Research, Tokyo, Japan; 2 Department of Computational Biology and Medical Sciences, Graduate School of Frontier Sciences, The University of Tokyo, Tokyo, Japan; 3 Laboratory of Proteomics for Drug Discovery, Laboratory of Clinical and Analytical Chemistry, Center for Drug Design Research, National Institutes of Biomedical Innovation, Health and Nutrition, Osaka, Japan; 4 Laboratory of Proteome Research, National Institutes of Biomedical Innovation, Health and Nutrition, Osaka, Japan; 5 Department of Thoracic Medical Oncology, The Cancer Institute Hospital, Japanese Foundation for Cancer Research, Tokyo, Japan; 6 Director, Cancer Chemotherapy Center, Japanese Foundation for Cancer Research, Tokyo, Japan

**Keywords:** Non-small-cell lung cancer, Cancer therapeutic resistance, Targeted therapies

## Abstract

Anaplastic lymphoma kinase (ALK) fusion is found in ~3%–5% of patients with non-small-cell lung cancers (NSCLCs). Although the third-generation ALK tyrosine kinase inhibitor (TKI) lorlatinib shows high clinical efficacy in ALK-positive NSCLC, most of the patients eventually relapse with acquired resistance. Recently, drug-tolerant persister (DTP) cells have been considered an important seed of acquired resistance cells. In this study, we established lorlatinib intermediate resistant cells from a patient-derived cell model. Glycogen synthase kinase 3 (GSK3) inhibitions significantly suppressed lorlatinib intermediate resistant cell growth. GSK3 inhibition also sensitized acquired resistance cells derived from alectinib-treated patients with or without secondary mutations to lorlatinib. Therefore, GSK3 plays a crucial role in developing acquired resistance against lorlatinib in ALK-positive NSCLC mediated by lorlatinib intermediate resistant cells and could be a potential molecular target to prevent acquired lorlatinib resistance and overcome ALK-TKI resistance.

## Introduction

There have been recent advancements in molecular targeted therapies, and many tyrosine kinase inhibitors (TKIs) have been developed for the treatment of non-small-cell lung cancer (NSCLC) patients with driver oncogenes^1,2^. Chromosomal rearrangements of anaplastic lymphoma kinase (*ALK*) occur in ~3%–5% of NSCLCs and in a broad range of other human cancers, such as anaplastic large cell lymphoma and colorectal cancers (CRCs)^3–6^. ALK fusion aberrantly induces activation of downstream signaling, including the mitogen-activated protein kinase (MAPK) and phosphoinositide 3-kinase (PI3K) pathways, strongly promoting tumorigenesis^7,8^. ALK-TKIs suppress ALK fusion-oriented oncogenic signaling and the growth of ALK-rearranged cancers. Thus, patients with NSCLC harboring *ALK* rearrangements receive clinical benefits from ALK-TKI treatment.

The third-generation ALK-TKI lorlatinib was developed to overcome acquired resistance after treatment with first- or second-generation ALK-TKIs^9,10^. Indeed, lorlatinib has shown a significant response in patients with first- or second-generation ALK-TKI failure, especially those with secondary resistance mutations in ALK^11,12^. Furthermore, on the basis of the results of a clinical trial, lorlatinib was recently approved by the Food and Drug Administration for the first-line treatment of ALK-positive NSCLC patients^13^. Although lorlatinib is an effective drug for the treatment of ALK-positive NSCLC patients, as with first- and second-generation ALK-TKIs, the majority of patients relapse due to acquired resistance. Several reports have described the emergence of compound mutations as resistance mechanisms against second- or later-line lorlatinib treatment^14–17^. In addition, some papers have reported several acquired resistance mechanisms through bypass signaling mediated by receptor tyrosine kinases (RTKs) and other signaling mediators such as ERBB family kinases and Src family kinases, the same as first- and second-generation ALK-TKIs^18,19^. Therefore, to further improve the clinical outcomes for ALK-positive NSCLC patients, it is necessary to comprehensively understand the underlying mechanisms of ALK-TKI resistance and develop novel therapeutic strategies to overcome it.

Recently, drug-tolerant persister (DTP) cells have been considered a reservoir for acquired resistance cell emergence across a wide range of cancer cells^20,21^. DTP cells are thought to have the potency to survive in the presence of inhibitors by escaping cell death induction and adapting to inhibitors and evolve into full-acquired resistance cells in a genetic or nongenetic manner in several cancers, including NSCLC with epidermal growth factor receptor (EGFR) mutation^22–24^. DTP cells provoke the emergence of diverse acquired resistance mechanisms with a variety of survival signals to escape inhibitors, and therefore, it is important to develop therapeutic strategies to eliminate DTP cells.

In this study, to understand the lorlatinib-tolerant and lorlatinib-resistant mechanisms and investigate therapeutic strategies to overcome lorlatinib persistence/resistance in ALK-positive NSCLC, we tried to establish lorlatinib tolerant persister cells with the intermittent lorlatinib treatment repeated 3 times, and succeeded to establish lorlatinib intermediate resistant cells and explored the mechanisms and effective inhibitors to eliminate them. Our in-house inhibitor library screening revealed that GSK3 inhibition with lorlatinib was crucial to suppress the viability of lorlatinib intermediate resistant cells. Moreover, we found that GSK3 inhibitor was also effective against acquired resistant cells by using ALK-positive NSCLC patient-derived cell (PDC) models without secondary resistant mutations in ALK. In addition, even the ALK-I1171N secondary mutation mediated alectinib-resistant cells, which are thought to be sensitive to lorlatinib, showed similar persister phenotype like the residual cells, and were further sensitized to lorlatinib by the GSK3 co-inhibition. Our findings may lead to the development of novel therapeutic strategies for overcoming acquired lorlatinib resistance.

## Results

### Lorlatinib intermediate resistant cells showed cross-resistance to multiple ALK-TKIs

**Fig. 1:**
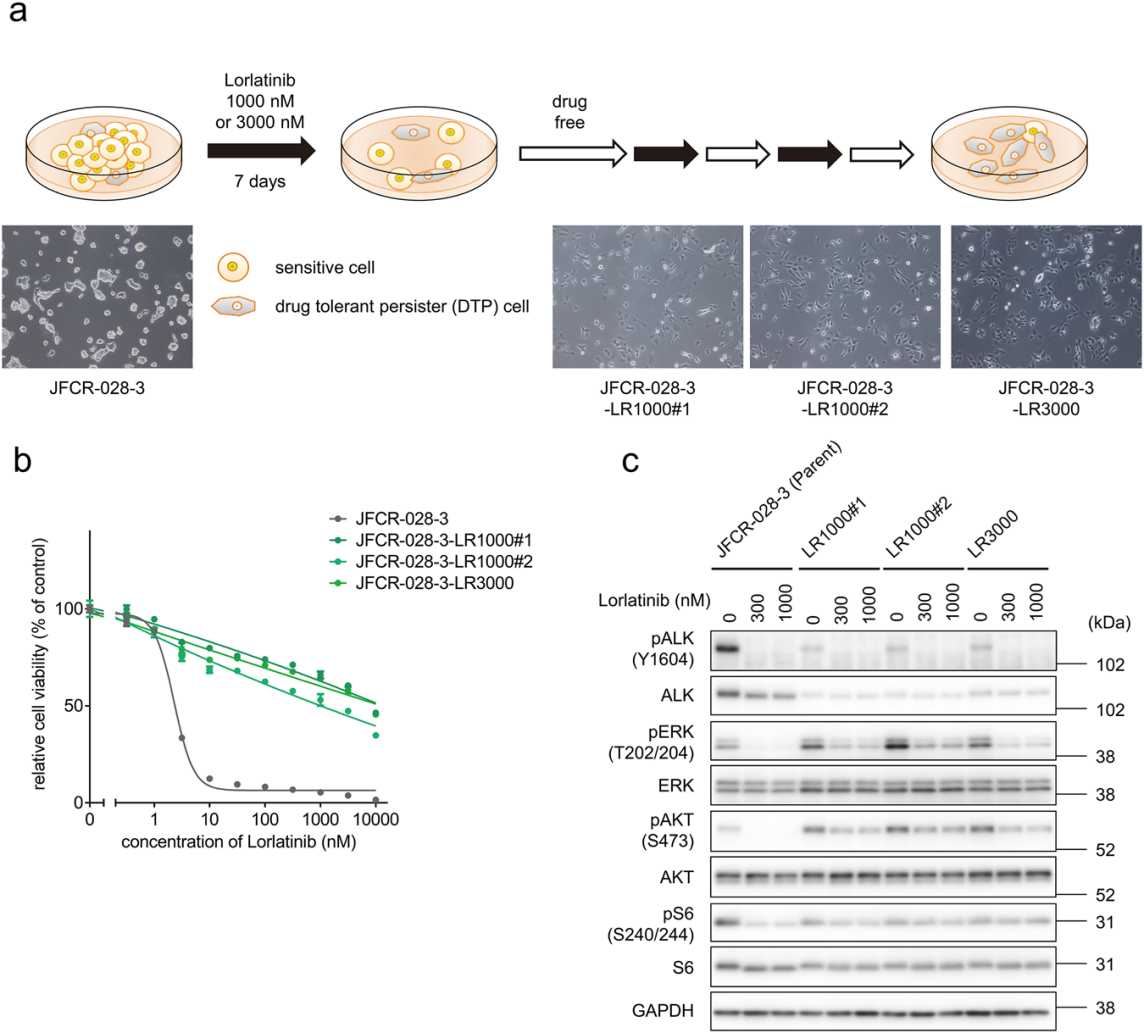
Lorlatinib intermediate resistant cells derived from JFCR-028-3 parental cells showed intermediate resistance to lorlatinib. **a** Schematic of the workflow to establish lorlatinib intermediate resistant cells from JFCR-028-3 parental cells, established after three cycles of drug treatment and a drug holiday. Scale bar = 100 μm. **b** Cell viability of JFCR-028-3 parental and lorlatinib intermediate resistant cells (*n* = 3). Each point represents the mean ± SD of three replicates. **c** Western blot analysis showing suppression of phospho-ALK and its downstream signaling in JFCR-028-3 parental and lorlatinib intermediate resistant cells treated with indicated concentrations of lorlatinib for 3 h. GAPDH was used as a loading control.

To establish lorlatinib resistant cells in vitro, ALK-TKI-sensitive patient-derived JFCR-028-3 parental cells obtained from the pleural effusion of a patient with EML4-ALK-positive NSCLC before alectinib treatment were exposed to high concentrations of lorlatinib (1 or 3 µM) for 1 week. The small fraction of remaining cells were cultured without lorlatinib for a few weeks until the cell growth was recovered. After the drug holiday, the cells were re-treated with the same concentrations of lorlatinib for three cycles. After the third cycle of lorlatinib exposure, we designated the cells as JFCR-028-3-LR1000#1, JFCR-028-3-LR1000#2, and JFCR-028-3-LR3000 (Fig. 1a). These established lorlatinib resistant cells were highly resistant to lorlatinib and other clinically approved ALK-TKIs (Fig. 1b and Supplementary Fig. 1a–d). To determine whether these lorlatinib resistant cells lose lorlatinib resistance after a drug holiday, JFCR-028-3-LR1000#1, JFCR-028-3-LR1000#2, and JFCR-028-3-LR3000 cells were cultured without lorlatinib for 12 days, and then the cells (now named JFCR-028-3-LR1000#1-d12, JFCR-028-3-LR1000#2-d12, and JFCR-028-3-LR3000-12) were treated with lorlatinib for 3 days. Although these lorlatinib resistant cells after a drug holiday were not completely resensitized to lorlatinib as the JFCR-028-3 parental cells, they showed intermediate sensitivity to lorlatinib compared with the lorlatinib-resistant cells without a drug holiday, suggesting that the lorlatinib sensitivity of established lorlatinib resistant cells is partially reversible and these cells are lorlatinib intermediate resistant state (Supplementary Fig. 1e). Since acquired mutation in the ALK kinase domain is a major resistance mechanism to ALK-TKIs, including lorlatinib, we investigated the mutation in the ALK kinase domain in these lorlatinib intermediate resistant cells. We did not observe any mutations, such as L1256F, which confers resistance to lorlatinib^15^. Therefore, we examined the status of ALK and its downstream signaling in the lorlatinib intermediate resistant cells. Western blot analysis revealed that lorlatinib treatment completely inhibited ALK autophosphorylation in both JFCR-028-3 parental and lorlatinib intermediate resistant cells (Fig. 1c); however, phospho-ERK and phospho-Akt were not fully suppressed in lorlatinib intermediate resistant cells, while phosphorylation of these proteins was more completely suppressed in JFCR-028-3 parental cells. Interestingly, Akt phosphorylation increased in lorlatinib intermediate resistant cells compared with JFCR-028-3 parental cells, whereas ALK autophosphorylation decreased in lorlatinib intermediate resistant cells. These results indicated that lorlatinib intermediate resistant cells acquire resistance to lorlatinib in an ALK-independent manner.

### Lorlatinib intermediate resistant cells were sensitive to Src family kinase inhibitor and GSK3 inhibitor combined with lorlatinib

**Fig. 2:**
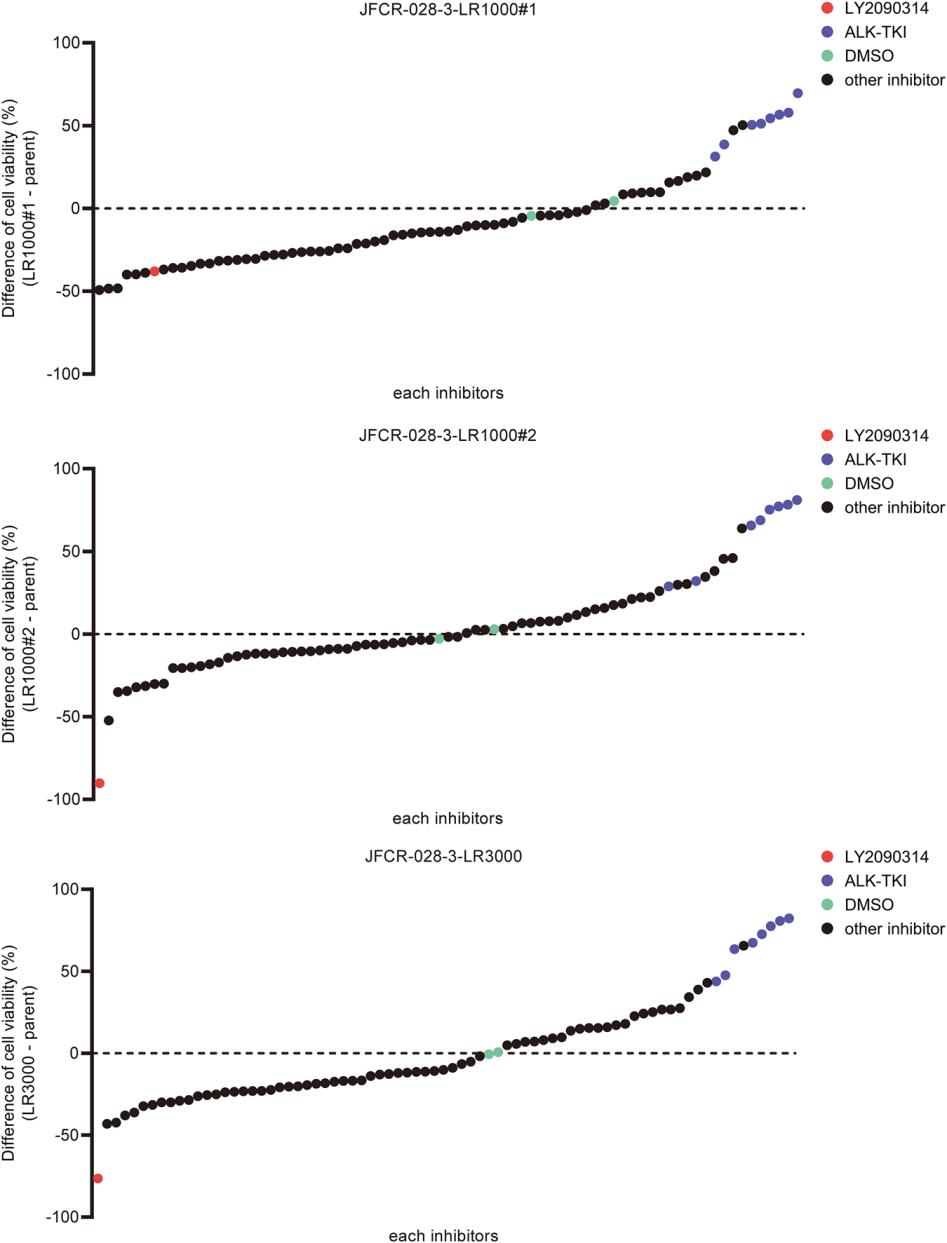
Focused inhibitor library screening revealed that GSK3 inhibitors specifically suppressed lorlatinib intermediate resistant cell viability. Difference in drug sensitivity to each drug between JFCR-028-3 lorlatinib intermediate resistant cells and JFCR-028-3 parental cells. The difference of drug sensitivity was calculated from the cell viability of JFCR-028-3 lorlatinib intermediate resistant cells and JFCR-028-3 parental cells in each drug (*n* = 2). Relative cell viability was calculated from each value divided by the DMSO control.

**Fig. 3:**
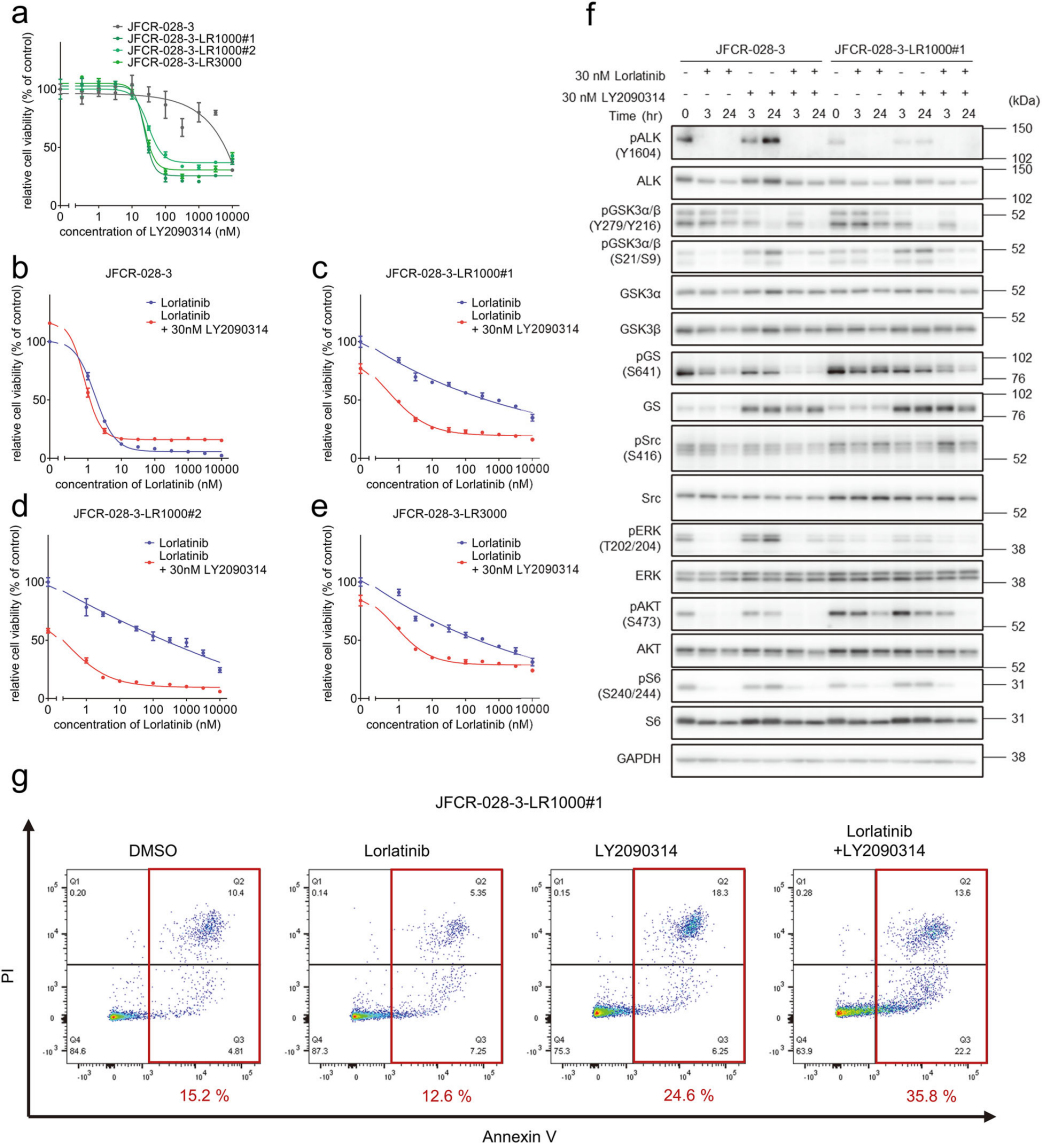
GSK3 inhibitors resensitized lorlatinib intermediate resistant cells to lorlatinib. **a** Cell viability of JFCR-028-3 parental and lorlatinib intermediate resistant cells treated with the indicated concentration of LY2090314 for 72 h (*n* = 3). Each point represents the mean ± SD of three replicates. Cell viability of **b** JFCR-028-3 parental and **c–e** lorlatinib intermediate resistant cells treated with the indicated concentration of lorlatinib in the presence or absence of a fixed concentration of LY2090314 for 72 h (*n* = 3). Each point represents the mean ± SD of three replicates. **f** Immunoblot analysis of indicated proteins after treatment of JFCR-028-3 parental and JFCR-028-3-LR1000#1 cells with indicated concentrations of lorlatinib in the absence or presence of LY2090314 for 0, 3, and 24 h. GAPDH was used as a loading control. **g** Apoptosis assay of JFCR-028-3-LR1000#1 cells treated with the 30 nM lorlatinib and 100 nM LY2090314. Apoptosis was evaluated using Annexin-V and PI staining after 72 h of the indicated drug treatment. The apoptotic cells were shown in red square and the percentage of apoptotic cells is shown in red value.

To explore effective inhibitors of lorlatinib intermediate resistant cells, drug screening with an in-house focused inhibitor library, mainly composed of clinically approved drugs and inhibitors under clinical development, was conducted in the absence or presence of 100 nM lorlatinib. This screening revealed that lorlatinib intermediate resistant cells show marked sensitivity to multiple Src family kinase inhibitors in combination with lorlatinib (Supplementary Fig. 2). Especially, dasatinib, a Src family kinase inhibitor, resensitized all three lorlatinib intermediate resistant cell types to lorlatinib (Supplementary Fig. 3a–d). To confirm whether Src plays an important role in lorlatinib resistance in these lorlatinib intermediate resistant cells, we evaluate the effect for cell viability by knocking down Src with specific siRNAs in JFCR-028-3-LR1000#1 cells. Silencing Src partially sensitized JFCR-028-3-LR1000#1 cells against lorlatinib (Supplementary Fig. S4a, b), indicating that lorlatinib intermediate resistant cells partially depend on Src family kinase-mediated signaling to survive in the presence of lorlatinib. Furthermore, LY2090314, a potent GSK3 inhibitor, significantly decreased the cell viability of lorlatinib intermediate resistant cells (LR1000#1, LR1000#2, and LR3000) in both the absence and the presence of lorlatinib (Fig. 2). Consistent with the results of inhibitor library screening, all lorlatinib intermediate resistant cells showed growth inhibition at a lower concentration of LY2090314 compared with JFCR-028-3 parental cells (Fig. 3a), indicating that lorlatinib intermediate resistant cells are more dependent on GSK3-mediated signaling for survival and proliferation. In addition, to confirm the combinational effect of lorlatinib and LY2090314, we evaluated the sensitivity to lorlatinib with a fixed concentration of LY2090314. JFCR-028-3 parental cells showed no difference in sensitivity to lorlatinib monotherapy and the lorlatinib combination with LY2090314 (Fig. 3b). In lorlatinib intermediate resistant cells, lorlatinib monotherapy partially suppressed cell viability, while the lorlatinib combination treatment with LY2090314 significantly inhibited the growth of lorlatinib intermediate resistant cells compared with lorlatinib monotherapy (Fig. 3c–e). TWS119, another potent GSK3 inhibitor, also demonstrated a similar combination effect in lorlatinib intermediate resistant cells but not in JFCR-028-3 parental cells (Supplementary Fig. 5a–d). To further determine the mechanisms underlying this combination effect, we examined cellular signaling by Western blot analysis. LY2090314 treatment induced the dephosphorylation of GSK3α (pY279) and GSK3β (pY216) as activation sites and the phosphorylation of GSK3α (pS21) and GSK3β (pS9) as inactivation sites in lorlatinib intermediate resistant and JFCR-028-3 parental cells (Fig. 3f). Furthermore, we examined the phosphorylation of glycogen synthase (GS), at serine 641 which is directly phosphorylated by GSK3. Interestingly, lorlatinib suppressed the phosphorylation of GS (S641) in JFCR-028-3 parental cells, but lorlatinib slightly suppressed the phosphorylation in JFCR-028-3-LR1000#1 cells. However, the combination of lorlatinib with LY2090314 reduced the phosphorylation of GS in JFCR-028-3-LR1000#1 cells. In JFCR-028-3-LR1000#1 cells, the combination of lorlatinib with LY2090314 decreased Akt phosphorylation more strongly compared with lorlatinib monotherapy. Similarly, in JFCR-028-3-LR1000#2 and JFCR-028-3-LR3000 cells, we observed suppression of phospho-Akt after treatment with lorlatinib monotherapy and the lorlatinib–LY2090314 combination (Supplementary Fig. 5e). Although we evaluated the phosphorylation of Src to examine whether LY2090314 inhibited Src signaling, LY2090314 did not show marked suppression of the phosphorylation of Src. In addition, we evaluated the effect of lorlatinib–LY2090314 combination on apoptosis. As the results, the combination drug treatment induced marked apoptosis in three lorlatinib intermediate resistant cells compared with lorlatinib or LY2090314 monotherapy (Fig. 3g and Supplementary Fig. 6a, b). To confirm whether GSK3 is crucial for cell viability in these lorlatinib intermediate resistant cells, we evaluate the cell viability of JFCR-028-3-LR1000#1 cells in GSK3 knockdown. Knockdown of GSK3β, but not GSK3α, partially sensitized JFCR-028-3-LR1000#1 cells to lorlatinib (Supplementary Fig. S7a, b), indicating that lorlatinib intermediate resistant cells depend on the signal pathways mediated by GSK3, especially GSK3β in part, to survive in the presence of lorlatinib. We next hypothesized that the combination of lorlatinib and a GSK3 inhibitor circumvents the emergence of resistant clones, and we performed colony formation assay to confirm our hypothesis. While several clones survived in lorlatinib monotherapy for 1 week treatment, these clones were diminished in the combination with LY2090314 (Supplementary Fig. S8). These findings suggested that a GSK3 inhibitor combined with lorlatinib might be effective in suppressing growth and inducing apoptosis of lorlatinib intermediate resistant cells.

### GSK3 inhibitor combined with ALK-TKIs was effective in ALK-TKI-resistant patient-derived cells

**Fig. 4:**
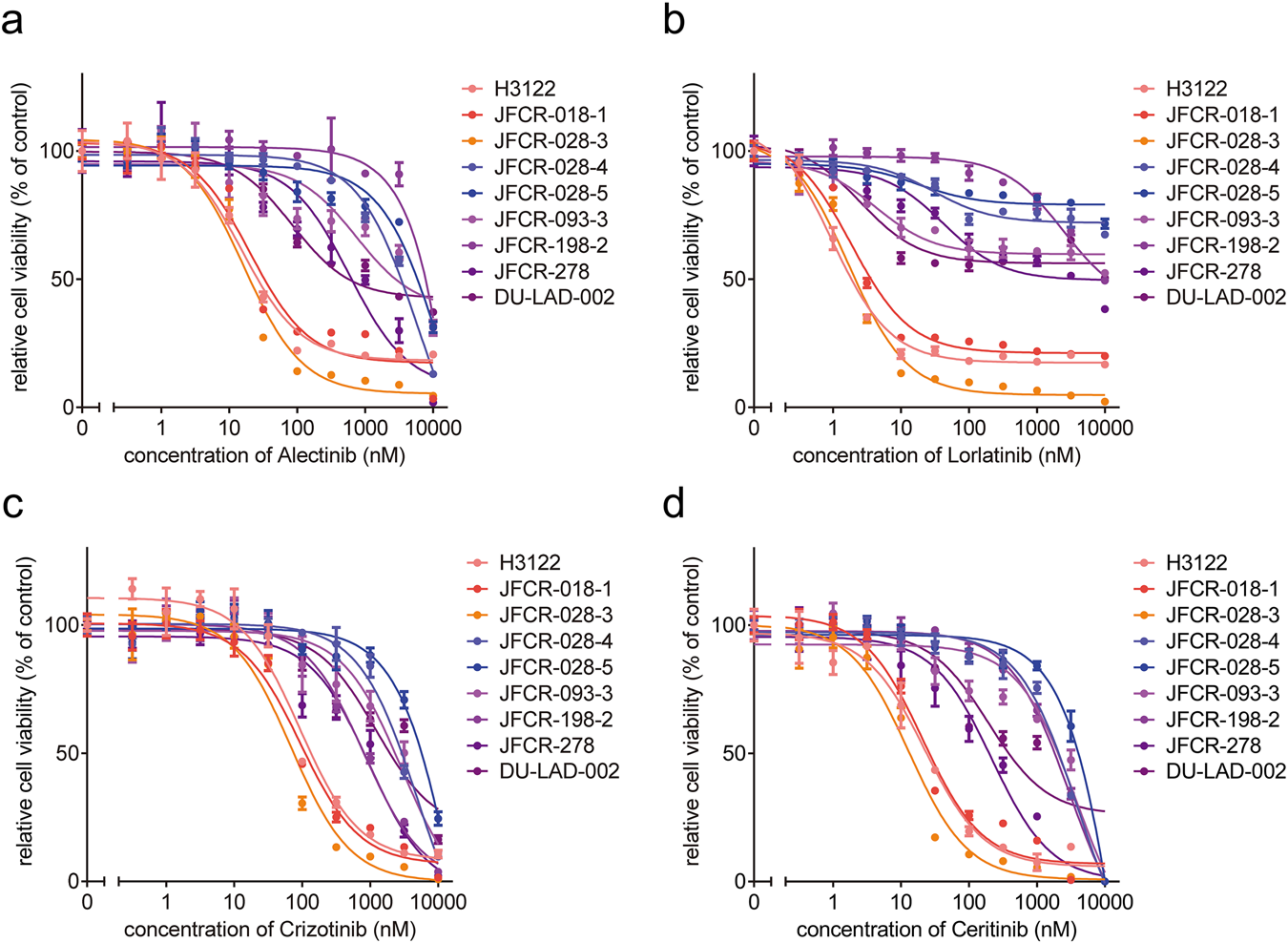
Alectinib-failure PDCs were resistant to multiple ALK-TKIs. Cell viability of ALK-positive PDCs treated with the indicated concentration of **a** alectinib, **b** lorlatinib, **c** crizotinib, and **d** ceritinib for 72 h was measured (*n* = 3). Each point represents the mean ± SD of three replicates.

**Fig. 5:**
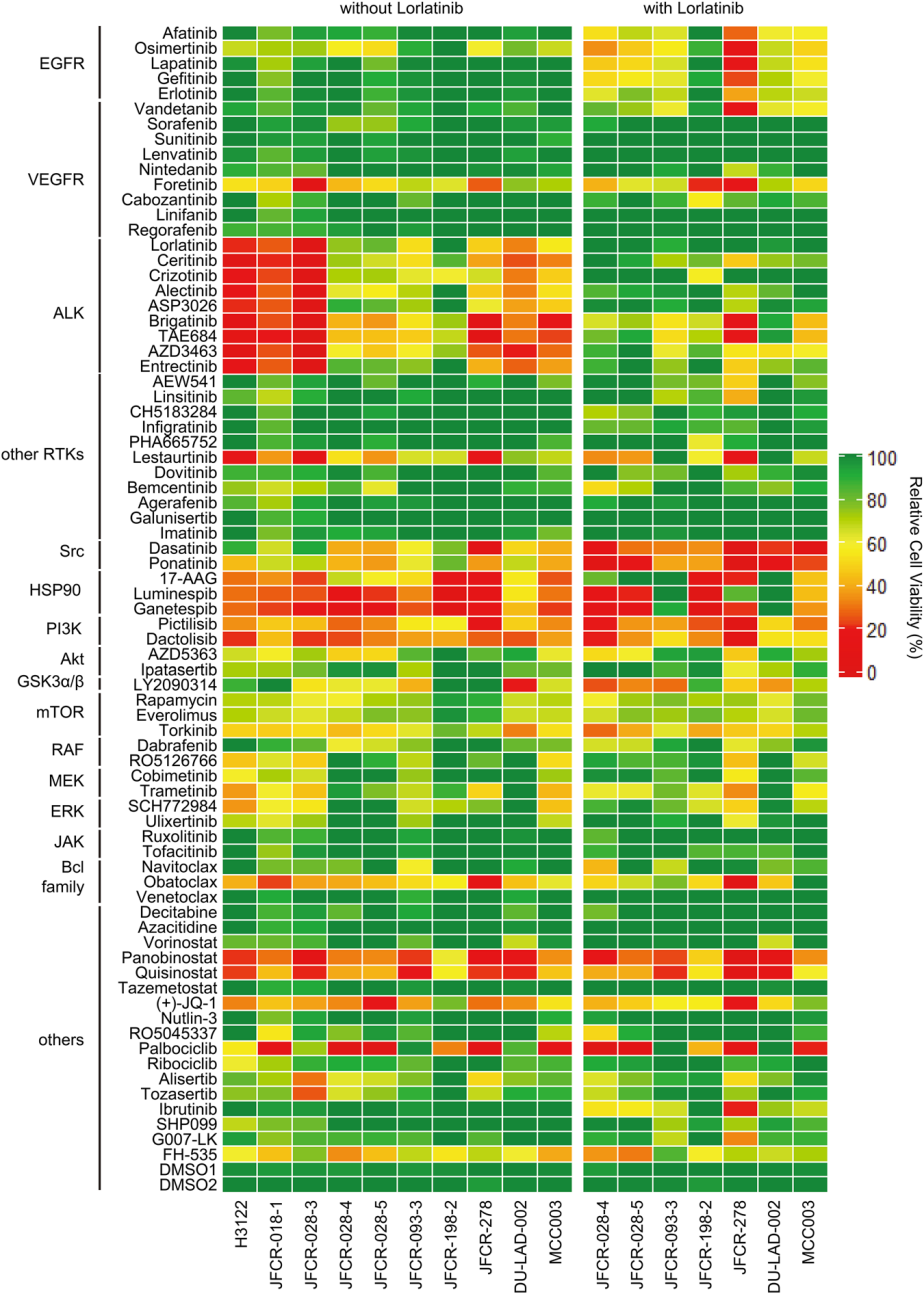
Drug sensitivity profile of ALK-positive cancer cell lines and PDCs using focused inhibitor library screening. Cell viability of ALK-positive cancer cell lines and PDCs treated with each inhibitor with or without 300 nM lorlatinib for 72 h was measured (*n* = 2). Relative cell viability was calculated from each value divided by the DMSO control.

**Fig. 6:**
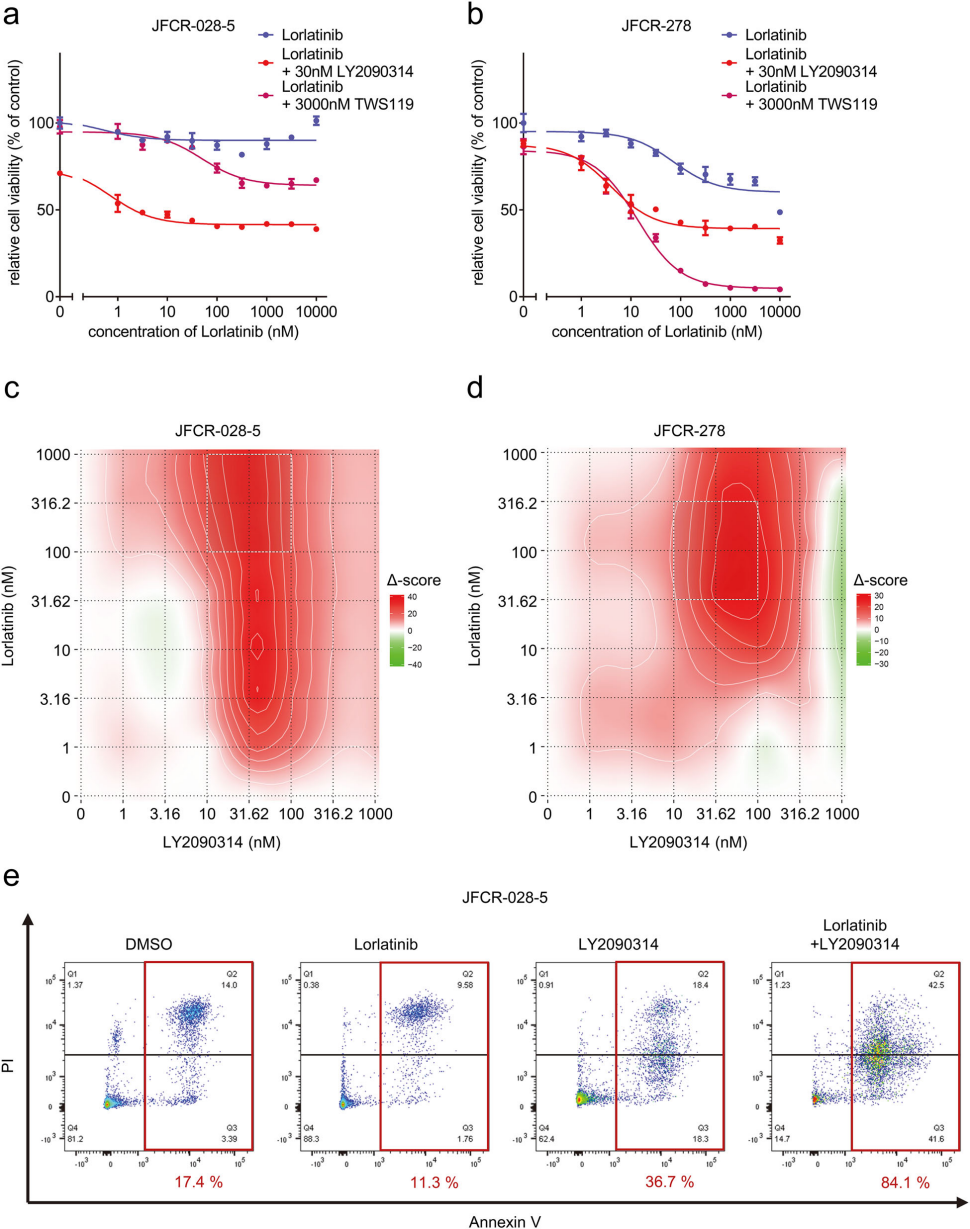
PDCs established from alectinib-failure patients showed high resistance to multiple ALK-TKIs but were vulnerable to GSK3 inhibition. Cell viability of ALK-positive cancer cell lines and PDCs treated with the indicated concentration of **a** alectinib and **b** lorlatinib for 72 h (*n* = 3) and **c** JFCR-028-5 and **d** JFCR-278 cells treated with the indicated concentration of lorlatinib with or without a fixed concentration of GSK3 inhibitors for 72 h (*n* = 3). Each point represents the mean ± SD of three replicates. Synergy distribution maps of **e** JFCR-028-5 and **f** JFCR-278 cells treated with the indicated concentration of lorlatinib in the absence or presence of the indicated concentration of LY2090314 for 24 h. Synergy scores were calculated using the ZIP model. The squares of white dots indicate areas with the highest ZIP synergy scores. **e** Evaluation of the effect on apoptosis of JFCR-028-5 cells treated with the of 30 nM lorlatinib and 100 nM LY2090314. Apoptosis was evaluated using Annexin-V and PI staining after 72 h of the indicated drug treatment. The apoptotic cells were shown in red square and the percentage of apoptotic cells is shown in red value.

To investigate effective inhibitors to overcome acquired resistance to ALK-TKIs, we established several patient-derived cells (PDCs) obtained from ALK fusion-positive NSCLC patients with alectinib-failure. Similar to a clinical setting, alectinib-resistant PDCs (JFCR-028-4, JFCR-028-5, JFCR-093-3, JFCR-198-2, JFCR-278, and DU-LAD-002) were strongly resistant to alectinib compared with ALK-TKI-sensitive cells (H3122, JFCR-018-1, and JFCR-028-3) (Fig. 4a). In these PDCs, we observed no mutation associated with ALK-TKI resistance in the ALK kinase domain. In addition, the PDCs were highly resistant to other ALK-TKIs, including lorlatinib (Fig. 4b–d), indicating that they acquire resistance to alectinib and lorlatinib in an ALK-independent manner. To explore promising combination strategies with lorlatinib, we again performed focused inhibitor library screening with or without 300 nM lorlatinib by using PDCs. Interestingly, drug sensitivity profiling revealed that LY2090314 suppressed cell viability in five of six resistant cell lines without secondary mutation in ALK (JFCR-028-4, JFCR-028-5, JFCR-093-3, JFCR-278, and DU-LAD-002) in the presence of lorlatinib (Fig. 5). To confirm the combination effect of ALK-TKIs with GSK3 inhibitors, we evaluated the drug sensitivities of these PDCs to lorlatinib with a fixed concentration of GSK3 inhibitors. GSK3 inhibitors, particularly LY2090314, induced marked resensitization of JFCR-028-5 and JFCR-278 cells to lorlatinib and alectinib (Fig. 6a, b and Supplementary Fig. 9a, b). Furthermore, to evaluate whether the combined effect of lorlatinib and LY2090314 is additive or synergistic, we performed an interactive analysis of multidrug combination profiling using the zero interaction potency (ZIP) model. The ZIP model captures drug interaction relationships by comparing the change in the potency of dose–response curves between individual drugs and their combinations, and then synergy analysis using this model can not only assess whether the combination effect is antagonistic, additive, or synergistic but also identify synergistic and antagonistic dose regions^25^. The combination of lorlatinib and GSK3 inhibitors showed a marked synergistic effect in JFCR-028-5 and JFCR-278 cells (Fig. 6c, d). The synergy assay also indicated the highest ZIP synergy scores at LY2090314 concentrations of 10–100 nM, suggesting that LY2090314 is effective in combination with lorlatinib, even at low concentrations. For further investigation of the combination effect on cellular signaling, we examined the downstream signaling of ALK and GSK3 using Western blot analysis. The LY2090314 combined with lorlatinib tended to decrease GS, Akt, and S6 phosphorylation compared with lorlatinib monotherapy (Supplementary Fig. 9c, d). Apoptosis assay also showed that the combination of lorlatinib and LY2090314 dramatically induced apoptosis in JFCR-028-5 and JFCR-278 cells compared with lorlatinib monotherapy (Fig. 6e and Supplementary Fig. 10). Although we evaluated cell viability of JFCR-278 cells in the presence of lorlatinib with or without GSK3 knockdown by siRNAs, lorlatinib sensitivity of JFCR-278 cells was changed by neither GSK3α nor GSK3β knockdown (Supplementary Fig. S11a, b), suggesting that other signals targeted by LY2090314 and TWS119 may also contribute to the cell survival of JFCR-278 under ALK inhibition. Additionally, we performed phosphoproteomics analysis to obtain insight into the mechanisms underlying GSK3 inhibition-induced suppression of cell growth in cells with acquired resistance to ALK-TKIs. We compared the phosphorylation status between JFCR-028-3 cells, as an ALK-TKI naive cell model, and JFCR-028-4 and JFCR-028-5 cells, as ALK-TKI acquired resistance cell models to directly compare the differences in a patient. Phosphoproteomics analysis revealed that 184 phosphorylated peptides significantly increased more than twofold in JFCR-028-4 cells compared with JFCR-028-3 cells (Supplementary Fig. 12a). Similarly, 233 phosphorylated peptides significantly increased more than twofold in JFCR-028-5 cells compared with JFCR-028-3 cells (Supplementary Fig. 12b). We next estimated proteins upstream of the phosphorylated proteins differentially expressed in JFCR-028-4 and JFCR-028-5 cells compared with JFCR-028-3 cells using a web-based kinase enrichment analysis tool (KEA2)^26^. GSK3β was significantly enriched in both JFCR-028-4 and JFCR-028-5 cells, while GSK3α was enriched but not significantly in JFCR-028-5 cells (Supplementary Fig. 12c, d). Western blot analysis revealed that the phosphorylation of GSK3β was upregulated in not only JFCR-028-5 and JFCR-278 but also the lorlatinib intermediate resistant cells (Supplementary Fig. 13), suggesting that GSK3, particularly GSK3β, might be an upstream core regulator in cells with acquired resistance to ALK-TKIs. These findings indicated that GSK3 inhibition is effective in overcoming several acquired resistances to multiple ALK-TKIs through ALK-independent mechanisms.

### GSK3 inhibitor enhanced the efficacy of lorlatinib in ALK mutation-mediated acquired resistance

Lorlatinib has potent inhibitory activity against ALK-TKI-resistant mutations, such as the ALK-I1171N mutation, one of the major alectinib-resistant mutations. MCC003 cells are resistant to alectinib but sensitive to lorlatinib^15,27^. MCC003 cells were established from an ALK-positive NSCLC patient with the EML4-ALK I1171N mutation and alectinib-failure. Lorlatinib significantly inhibited MCC003 cell growth and proliferation; however, a part of the cells remained even at a high concentration of lorlatinib (Supplementary Fig. 14a).

Interestingly, drug sensitivity profiling through the focused inhibitor library showed that the combined treatment of lorlatinib and LY2090314 more strongly decreased MCC003 cell viability compared with lorlatinib monotherapy (Fig. 5). Cell viability assay showed that LY2090314 combined with lorlatinib more strongly decreased MCC003 cell viability compared with lorlatinib monotherapy, as well as TWS119 (Supplementary Fig. 14a). Moreover, similar to our results with lorlatinib intermediate resistant cells and acquired resistance PDCs, Western blot analysis showed that Akt and S6 phosphorylation was more significantly suppressed after treatment with the combination of lorlatinib and LY2090314 (Supplementary Fig. 14b). These findings illustrated that GSK3 inhibition is also effective in enhancing lorlatinib sensitivity in acquired resistance cells through ALK mutation.

### Combined inhibition of EGFR and Src family kinase resensitized ALK-TKI-resistant patient-derived cells to lorlatinib

**Fig. 7:**
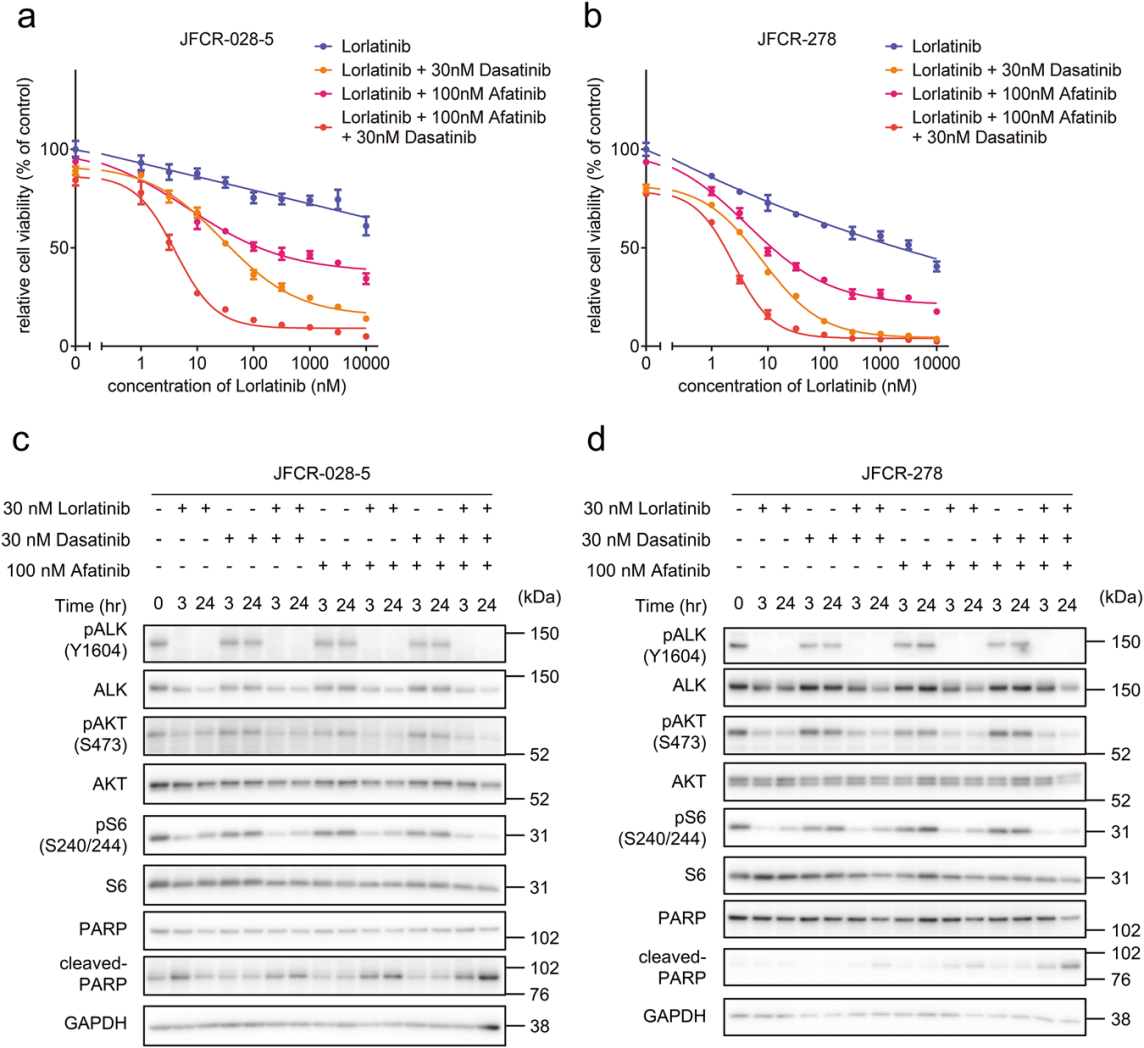
Combination therapy of lorlatinib, Src family kinase inhibitor, and EGFR inhibitor showed significant suppression of cell viability in lorlatinib-acquired resistance cells. Cell viability of **a** JFCR-028-5 and **b** JFCR-278 cells treated with the indicated concentration of lorlatinib with or without a fixed concentration of dasatinib or afatinib for 72 h (*n* = 3). Each point represents the mean ± SD of three replicates. Western blot analysis showing suppression of phospho-ALK and its downstream signaling in **c** JFCR-028-5 and **d** JFCR-278 cells treated with 30 nM lorlatinib in the absence or presence of the indicated concentration of dasatinib or afatinib for 0, 3, and 24 h. GAPDH was used as a loading control.

The focused inhibitor library screening revealed that multiple Src family kinase inhibitors and EGFR inhibitors in combination with lorlatinib are consistently effective in several first- or second-generation ALK-TKI-resistant PDCs (Fig. 5)^28^. We evaluated the effects of combined treatment with a fixed dose of an Src inhibitor dasatinib and a pan-ERBB family inhibitor afatinib. The ALK-TKI-resistant JFCR-028-5 and JFCR-278 cells were sensitized to lorlatinib by combining it with afatinib or dasatinib (Fig. 7a, b). The phosphor-RTK array demonstrated that ALK inhibition increased EGFR phosphorylation, and phosphor-EGFR was higher in JFCR-028-5 cells compared with ALK-TKI-naive JFCR-028-3 parental cells (Supplementary Fig. 15). Interestingly, these cells were more significantly sensitized by dual inhibition of EGFR and Src family kinases (Fig. 7a, b). Next, we investigated the combination therapy effect on cellular signaling. Western blot analysis of JFCR-028-5 cells revealed that the dasatinib combined with lorlatinib decreased Akt and S6 phosphorylation compared with lorlatinib monotherapy (Fig. 7c and Supplementary Fig. 16a). However, the dasatinib combined with lorlatinib suppressed phosphor-Akt and phosphor-S6 in JFCR-278 cells (Fig. 7d and Supplementary Fig. 16b). Similar to JFCR-028-5 cells, the combination treatment of lorlatinib, dasatinib, and afatinib significantly suppressed Akt and S6 phosphorylation. Interestingly, dasatinib and afatinib partially suppressed the phosphorylation of GSK3, and then the combination of lorlatinib with these inhibitors reduced the phosphorylation of GS more significantly compared with lorlatinib monotherapy. Although silencing GSK3 did not sensitize JFCR-278 cells against lorlatinib, silencing Src partially suppressed cell growth of JFCR-278 in presence of lorlatinib (Supplementary Fig. 17a and b), indicated that EGFR and Src might have the crosstalk with GSK3 and partially contributes to GSK3 signaling in JFCR-278 cells as one of the upstream molecules of GSK3. These results indicated that dual inhibition of Src and EGFR may be more effective in overcoming resistance to multiple ALK-TKIs compared with inhibition of each alone.

## Discussion

In this study, we established lorlatinib resistant cells showing intermediate resistance to lorlatinib and other ALK-TKIs. In our lorlatinib resistant cell models, the lorlatinib-resistant characteristics were not completely but partially reversible. Reversibility of drug sensitivity is considered as one of the important characteristics of drug-tolerant persister cells^21,29^. Considering these points, our lorlatinib intermediate resistant cells might be a pool of heterogenous subpopulation consists of the cells acquired complete resistance, and the cells retaining reversibility of lorlatinib sensitivity. Thus, our established lorlatinib intermediate resistant cells could be seeds of complete resistant cells. In this study, we identified GSK3 as a key molecule to survive in lorlatinib intermediate resistant cells and acquired resistant cells. GSK3 inhibitors, including LY2090314, show significant growth suppression and apoptosis induction of acquired resistance cells with a mesenchymal phenotype in EGFR mutation-positive NSCLC^30^. We also checked the EMT markers such as E-cadherin and N-cadherin in lorlatinib intermediate resistant cells and acquired resistant cells harboring ALK-fusion, and then the expression of these EMT-associated proteins was similar between JFCR-028-3 parental cells and the lorlatinib intermediate resistant cells and the acquired resistant cells, although the expression of vimentin was upregulated in the lorlatinib intermediate resistant cells and the acquired resistant cells (Supplementary Fig. 13). Interestingly, the protein expression of ALK-fusion was downregulated in the lorlatinib intermediate resistant cells. Droplet digital PCR and qRT-PCR unveiled that the gene expression of not only *EML4-ALK* but also *EML4* was downregulated (Supplementary Fig. 18). These results indicated that the expression of *EML4* decreased in the lorlatinib intermediate resistant cells, resulting in downregulation of *EML4-ALK* fusion gene expression.

Alectinib is approved for the treatment of ALK-positive NSCLC and has been widely used as first-line therapy. However, about half of the patients experience acquired resistance within 3 years. About half of these relapsed patients harbor secondary mutations in the ALK domain, and a small fraction of resistant tumors harbor *cMET* amplification. However, in the rest, the mechanisms underlying acquired resistance are still unclear^31–34^. Our PDCs (JFCR-028-3, JFCR-028-4, and JFCR-028-5 cells) were obtained from the same patient. JFCR-028-3 parental cells were derived from the pleural effusion of prior alectinib treatment, JFCR-028-4 cells from the pleural effusion during alectinib treatment, and JFCR-028-5 cells from the pleural fluid after alectinib treatment. Both JFCR-028-4 and JFCR-028-5 cells had no secondary ALK mutation. JFCR-028-5 cells showed higher resistance to alectinib compared with JFCR-028-4 cells, suggesting that JFCR-28-4 cells are intermediate alectinib-resistant cells. Remarkably, the drug sensitivity profile of JFCR-028-4 and JFCR-028-5 cells was similar to that of lorlatinib intermediate resistant cells established from JFCR-028-3 parental cells (JFCR-028-3-LR1000#1, JFCR-028-3-LR1000#2, and JFCR-028-3-LR3000 cells). These results indicated that lorlatinib intermediate resistant cells and acquired resistance cells depend on similar signaling pathways, including GSK3, for cell growth and survival. The evolutionary intermediates of acquired resistance may present temporally under the presence of ALK-TKIs in the ALK-positive NSCLC model^18^. In addition, acquired resistance cells can emerge from drug-tolerant persister cells in other driver oncogenes–positive cancer cells^20^. Therefore, although lorlatinib intermediate resistant cells and clinically-acquired resistance cells were established under different conditions of drug exposure, our results support the fact that acquired resistance cells, such as JFCR-028-4 and JFCR-028-5 cells, may emerge from mediators like lorlatinib intermediate resistant cells.

Lorlatinib is highly effective in overcoming ALK mutants, such as I1171N and V1180L, which cause acquired resistance to alectinib^9^. Indeed, lorlatinib suppresses cell growth and induced apoptosis in alectinib acquired resistance PDCs, MCC003 cells, which harbor the I1171N mutation. In contrast; the MCC003 cell remains after a high concentration of lorlatinib treatment. In addition, ALK-I1171N secondary mutation-mediated alectinib-resistant cells, which are thought to be sensitive to lorlatinib, show a similar persister phenotype like the residual cells and are further sensitized to lorlatinib by GSK3 co-inhibition. Thus, further investigation using acquired resistant models with ALK mutations may reveal that inhibition of GSK3 is effective against acquired resistance in an ALK-dependent manner.

Src family kinases, especially Src, and EGFR are key players in mechanisms underlying acquired resistance to ALK-TKIs, including lorlatinib^19,28,34–37^. Dual inhibition of Src family kinases and EGFR combined with lorlatinib might be an effective strategy for overcoming acquired resistance to ALK-TKIs, including lorlatinib. In particular, the Src family kinase inhibitor dasatinib significantly demonstrates growth suppression in lorlatinib intermediate resistant cells and acquired resistant cells, suggesting that Src family kinases are also core molecules involved in the survival of lorlatinib intermediate resistant cells and acquired resistant cells, the same as GSK3. Indeed, Src and EGFR have crosstalk by activating each other^38–40^, and Src is reported to phosphorylate and activate GSK3 directly^41^. In this study, a single knockdown of GSK3 and Src partially restored the sensitivity of lorlatinib. In addition, LY2090314 and dasatinib combined with lorlatinib suppressed the phosphorylation of GS, which was not completely reduced by lorlatinib monotherapy in lorlatinib intermediate resistant cells and the acquired resistant cells. These results suggested that GSK3 and Src might have crosstalk and share the same downstream signals. However, our result also indicated that dependency on GSK3 and Src is different across resistant cells. Thus, in the future, it is needed to clarify molecular markers able to identify which targets are most beneficial targets in each cell to completely suppress the growth of both lorlatinib intermediate resistant cells and cells with acquired resistance to ALK-TKIs.

This study had several limitations. First, the acquired resistance PDC lines used were mainly established from alectinib-failure, not lorlatinib-failure, patients. However, the PDC lines showed strong resistance to lorlatinib as well as alectinib. In the future, it will be important to evaluate the effectiveness of GSK3 inhibitors in PDCs established from lorlatinib-failure patients. Second, we did not evaluate the efficacy of the combination of lorlatinib and GSK3 inhibitors in vivo. Third, it remains unclear why lorlatinib intermediate resistant cells and several acquired resistance cells tend to depend on GSK3 signaling for survival and proliferation. However, in EGFR-mutation-positive NSCLC, insulin-like growth factor 1 receptor (IGF-1R) plays a crucial role in the emergence of cells tolerant to EGFR-TKIs^42^. GSK3 is a key component of downstream signaling mediated by RTKs and other signal proteins, and in this study, phosphoproteome analysis indicated GSK3 enrichment. Thus, GSK3 may provoke cell survival as a hub molecule in lorlatinib intermediate resistant cells and cells with acquired resistance to ALK-TKIs, although further investigation is needed in the future. However, GSK3 plays an indispensable role in glycometabolism in normal cells, and its inhibition may cause various adverse events^43,44^. In a phase 2 clinical trial of treatment of acute leukemia patients, the single agent LY2090314 showed limited clinical benefit but acceptable safety^45^. The combination of lorlatinib and LY2090314 shows efficacy even at low concentrations of LY2090314 in vitro, and LY2090314 may be a promising GSK3 inhibitor. However, further investigation using a mouse model is needed in the future to develop effective strategies for overcoming lorlatinib resistance using GSK3 inhibitors.

In conclusion, GSK3 inhibition shows efficacy against not only acquired resistance cells but also lorlatinib intermediate resistant PDCs. GSK3 inhibition is crucial to suppress the viability of lorlatinib intermediate resistant cells. Moreover, GSK3 inhibition is also effective against acquired resistance ALK-positive NSCLC PDC models without secondary resistant ALK mutations. This study provided new insights into the emergence of acquired resistance using mediators like lorlatinib intermediate resistant cells and proposed novel therapeutic strategies for improving clinical outcomes in ALK-positive NSCLC.

## Methods

### Cell lines and culture condition

H3122 cells were kindly gifted by Dr. Engelman JA and cultured in Roswell Park Memorial Institute (RPMI)-1640 medium (Wako Pure Chemical Industries, Ltd, Osaka, Japan) supplemented with 10% fetal bovine serum (FBS) and 100 µg/mL of kanamycin. ALK fusion-positive NSCLC PDC lines were established from patients, which provided informed consent for genetic and cell biological analyses performed in accordance with protocols approved by the Institutional Review Board of the Japanese Foundation for Cancer Research. MCC003 was established as previously shown in Okada et al., EBioMed 2019. In brief, MCC003 cells were established from pleural effusion of alectinib-resistant patient, and confirmed to express EML4-ALK I1171N mutant in both original pleural effusion and the established cell line (MCC003). ALK-TKI-naive EML4-ALK fusion-positive NSCLC PDC lines JFCR-018-1 and JFCR-028-3 and alectinib-resistant ALK fusion-positive NSCLC PDC lines JFCR-028-4, JFCR-028-5, JFCR-198-2, JFCR-278, DU-LAD-002, and MCC003 were cultured in RPMI-1640 medium and Ham’s F-12 medium with 20 mM 4-(2-hydroxyethyl)-1-piperazineethanesulfonic acid (HEPES; Nacalai Tesque Inc., Kyoto, Japan), 15% FBS, and 1× Antibiotic-Antimycotic Mixed Stock Solution (Nacalai Tesque). JFCR-093-3 cells were cultured in ACL-4 medium (Wako Pure Chemical Industries) supplemented with 10% FBS and 1x Antibiotic-Antimycotic Mixed Stock Solution. Lorlatinib intermediate resistant cells (JFCR-028-3-LR1000#1, JFCR-028-3-LR1000#2, and JFCR-028-3-LR3000) were established from JFCR-028-3 parental cells by treatment with 1 or 3 µM lorlatinib as de novo persistent resistant cells.

### Reagents

Lorlatinib, crizotinib, and brigatinib were purchased from ShangHai Biochempartner (Shanghai, China); alectinib and ceritinib from ActiveBiochem (Kowloon, Hong Kong); and dasatinib from SelleckChem (Houston, TX, USA). LY2090314 was purchased from AdooQ BioScience (Irvine, CA, USA) and TWS119 from Santa Cruz Biotechnology (Dallas, TX, USA). Brigatinib was dissolved in ethanol, and the other inhibitors were dissolved in dimethyl sulfoxide (DMSO) for cell culture experiments. Details of the other inhibitors for focused inhibitor library screening are shown in Supplementary Table 1.

### Cell viability assay

To evaluate cell viability, cells were seeded in triplicate at a density of 3000 cells/well in 96-well plates or 96-well collagen-coated plates (IWAKI, Shizuoka, Japan). After 72 h of drug treatment, the cells were incubated with CellTiter-Glo assay reagent (Promega Corporation, Madison, WI, USA) for 2 min. Following equivalation for 10 min, luminescence was measured using a Tristar LB941 microplate luminometer (Berthold Technologies, Oak Ridge, TN, USA). GraphPad Prism version 9.1.2 (GraphPad Software, La Jolla, CA, USA) was used to analyze and graphically display the data.

### Western blot analysis

Cells were seeded at a density of 1–3 × 10^5^ cells/well in 12-well plates or 12-well collagen-coated plates (IWAKI) and treated with the indicated concentration of drugs. Next, the cells were lysed using sodium dodecyl sulfate (SDS) lysis buffer containing 0.1 M Tris-HCl (pH 7.5), 10% glycerol, and 1% SDS and boiled at 100 °C for 5 min. Protein quantification of cell lysates was performed using a bicinchoninic acid (BCA) protein assay kit (Thermo Fischer Scientific, Waltham, MA, USA) according to the manufacturer’s instructions, and luminescence was measured using a Multiskan™ GO Microplate Spectrophotometer (Thermo Fischer Scientific). The cell lysates were adjusted to equal amounts of proteins using SDS lysis buffer, and 20% volume of 5× sample buffer containing 0.65 M Tris-HCl (pH 6.8), 20% 2-mercaptoethanol, 10% glycerol, 3% SDS, and 0.01% bromophenol blue was added. Equal amounts of proteins were applied to sodium dodecyl sulfate-polyacrylamide gel electrophoresis (SDS-PAGE) and immunoblotted. The following antibodies were purchased from Cell Signaling Technology (Danvers, MA, USA): total ALK (#3633, 1:2000), phospho-ALK (Y1604, #3341, 1:1000), total AKT (#4691, 1:1000), phospho-AKT (S473, #4060, 1:1000), total p42/44 ERK/MAPK (#9102, 1:1000), phospho-p42/44 ERK/MAPK (T202/Y204, #9101, 1:2000), total GSK3α (#4337, 1:1000), total GSK3β (#12456, 1:1000), phospho-GSK3α/β (S21/S9, #8566, 1:1000), total Glycogen Synthase (GS) (#3893, 1:1000), phospho-Glycogen Synthase (S641, #3891, 1:1000), total Src (#2123, 1:1000), phospho-Src (Y416, #6943, 1:1000), total S6 ribosomal protein (#2217, 1:1000), phospho-S6 ribosomal protein (S240/244, #5364, 1:8000), poly(ADP-ribose) polymerase (PARP) (#9542, 1:1000), cleaved PARP (#9541, 1:1000), E-cadherin (#3195, 1:1000), N-cadherin (#13116, 1:1000), and Vimentin (#5741, 1:1000). In addition, phospho-GSK3α/β (Y279/Y216) antibody was purchased from Abcam (#ab68476, Cambridge, UK, 1:1000), and glyceraldehyde 3-phosphate dehydrogenase (GAPDH) antibody was purchased from Millipore (MAB374, Burlington, MA, USA, 1:10,000). For signal detection, we used ECL Prime Western Blotting Detection Reagent (GE Healthcare, Chicago, IL, USA) or SuperSignal West Femto Maximum Sensitivity Substrate (Thermo Fischer Scientific). The signals were detected using Amersham Imager 600 (GE Healthcare) or Amersham Imager 800 (GE Healthcare).

### Inhibitor library screening

Cells were seeded in triplicate at a density of 3000 cells/well in 96-well plates or 96-well collagen-coated plates and treated with an original panel of the inhibitor library for 72 h. Next, the cells were incubated with CellTiter-Glo assay reagent (Promega) for 2 min. After equivalation for 10 min, luminescence was measured using a Tristar LB941 microplate luminometer (Berthold Technologies). The relative cell viability was calculated as a ratio of the DMSO control. In combination treatment with lorlatinib, the relative cell viability was recalculated as a ratio to lorlatinib monotherapy. The average relative cell viability of duplicates was calculated and graphically displayed as a heat map using ComplexHeatmap version 2.4.3 (R version 4.0.2)^46^. Original data are available in Supplementary Data 1.

### RNA interference for gene knockdown

Cells were transfected with the indicated concentration of siRNA mixed with Lipofectamin RNAiMAX Transfection Reagent (Thermo Fisher Scientific) in OPTI-MEM (1×) (Thermo Fisher Scientific) according to the manufacturer’s instructions. Cells were seeded at a density of 1–3 × 10^5^ cells/well in 6-well plates for cell lysate collection and at 3000 cells/well of triplicate in 96-well plates for cell viability assay. Cell lysate was collected after 48 h of seeding and analyzed by Western blotting described above. For the cell viability assay, cells were treated with lorlatinib to a final concentration of 30 nM after 24 h of seeding. After 72 h of drug treatment, the cells were collected and incubated with CellTiter-Glo assay reagent and luminescence was measured in the same methods described above. The following siRNA were purchased from Dharmacon (Lafayette, CO, USA): ON-TARGET plus Non-targeting Pool (D-001810-10-05) of UGGUUUACAUGUCGACUAA, UGGUUUACAUGUUGUGUGA, UGGUUUACAUGUUUUCUGA, and UGGUUUACAUGUUUUCCUA for siControl, ON-TARGET plus Human SRC (6714) siRNA (LQ-003175-00-0005) of CCAAGGGCCUCAACGUGAA for siSRC#1 and GGGAGAACCUCUAGGCACA for siSRC#2, ON-TARGET plus Human GSK3A (2931) siRNA (LQ-003009-00-0005) of GAAGGUGACCACAGUCGUA for siGSK3α#1 and GAGUUCAAGUUCCCUCAGA for siGSK3α#2, and ON-TARGET plus Human GSK3B (2932) siRNA (LQ-003010-00-0005) of GUUCCGAAGUUUAGCCUAU for siGSK3β#1 and GCACCAGAGUUGAUCUUUG for siGSK3β#2.

### Colony formation assay

Cells were seeded at a density of 1 × 10^5^ cells/well in 6-well plates in triplicate. After 48 h of seeding, cells were treated with the indicated inhibitors. Medium was changed every 3 days and cells were cultured with inhibitors for 1 week. Colonies were fixed in 4% paraformaldehyde phosphate buffer solution (Wako) for 15 min at room temperature and stained with 0.1% crystal violet (SIGMA, Kanagawa, Japan) in 10% ethanol (SIGMA) for 2 min at room temperature. After staining, pictures of the wells were taken.

### Apoptosis assay

Cells were seeded at a density of 1 × 10^5^ cells/well in 6-well plates. After overnight culture, cells were treated with the indicated concentration of drugs. All floating and adherent cells were collected after 72 h of drug treatment. Cells were stained with propidium iodide and Alexa Fluor 647 conjugated annexin V using a Annexin V/Dead Cell Apoptosis Kit (Thermo Fischer Scientific) for 15 min at room temperature. Measurement was performed using FACS Lyric (BD Bioscience, Franklin Lakes, NJ, USA) and FlowJo software (BD Bioscience) was used to analyze and graphically display the data.

### Synergistic effect assay

Cell viability was calculated as described in the Cell Viability Assay section. To evaluate the synergistic effect of combination therapy, synergistic scores were calculated based on the zero interaction potency (#ZIP#) reference model using the SynergyFinder 2.0 web application tool^47^.

### Phosphoproteomic analysis

JFCR-028-3, JFCR-028-4 and JFCR-028-5 cell lines were washed with PBS supplemented with cOmplete EDTA-free and PhosSTOP (Roche, Basel, Switzerland), and collected into 2.0 ml tubes with scrapers. After centrifugation, cell pellets were frozen quickly in liquid nitrogen, and stored at −80 °C until usage. Cell pellets were solubilized in phase transfer surfactant (PTS) buffer (50 mM NaHCO_3_, 12 mM Sodium N-Lauroylsarcosinate, 12 mM sodium deoxycholate)^48^ supplemented with cOmplete EDTA-free and PhosSTOP. Protein lysates were immediately boiled at 95 °C for 5 min, and sonicated for 30 min.

Global phosphoproteomics and phosphotyrosine (pY) proteomics were prepared as described previously^27^. Briefly, 2 mg of protein lysates were subjected to reduction, alkylation, and tryptic digestion. Protein lysates were incubated with trypsin (protein weight: 1/50) and Lys-C (protein weight: 1/50) for 16 h at 37 °C. After acidification with 1% trifluoroacetic acid (TFA) and centrifugation at 20,000 × *g* for 10 min, the supernatants were desalted with HLB OASIS column (Waters), and applied into Fe-IMAC column for phosphopeptide enrichment.

2.5% of digests were used for proteome analysis and the rest were used for global phosphoproteome and pY proteome analysis. Labeling of peptides and phosphopeptides with TMT 10plex reagents (Thermo Scientific, Bremen, Germany) was carried out according to the manufacturer’s protocol. After phosphopeptide labeling, 10 samples labeled with TMT reagents were pooled and divided into 50% for global phosphoproteomics and 50% for pY proteomics. For global phosphoproteomics, TMT-labeled phosphopeptides were fractionated with SCX/C18 stage-tip into 7 fractions^49^, and subsequently dried up. The fractions were reconstituted in 2% acetonitrile, 0.1%TFA before a measurement with LC-MS/MS. For phosphotyrosine proteomics, TMT-labeled phosphopeptides were subjected to enrichment of pY peptides with pY1000 antibody (CST signalings) as described previously^50^. Enriched pY peptides were dried up, and re-suspended in 10 µl of 2% ACN/1% TFA for LC-MS/MS analysis.

The measurements of global phosphoproteomics and pY proteomics were performed by Q Exactive Plus coupling an UltiMate 3000 Nano LC system (Thermo Scientific) and an HTC-PAL autosampler (CTC Analytics, Zwingen, Switzerland) in data-dependent mode. The UltiMate 3000 Nano LC system was operated with the gradients formed of Buffer A (2% acetonitrile and 0.1% formic acid) with 5–30% Buffer B (90% acetonitrile and 0.1% formic acid) over 135 min (global proteomics or 45 min (pY proteomics). The flow rate of the UltiMate 3000 Nano LC system was 280 nL/min. MS T Parameters of Q Exactive Plus were corresponding to the condition in a previous study^51^.

Phosphopeptide identification was performed by MaxQuant (version 1.5.1.2) supported by the Andromeda search engine^52^. Database search was done against the UniProt human database (released in January 2017) combined with 262 common contaminants. The enzyme specificity was corresponding to trypsin/P (the C-terminal of cleavage sites at the proline (P) bond allowed). Miss cleavages were tolerated up to two sites. A fixed modifications were set as carbamidomethylation at cysteine residue, and variable modifications were assumed that methionine oxidation and serine, threonine, and tyrosine phosphorylation occurred. Protein groups, peptides, and phosphosites were identified under each false discovery rate <0.01. Only phosphosites with more than 0.75 of localization probabilities were subjected to the following analysis.

The statistical analysis of the phosphoproteomic data was performed with Perseus 1.5.6.0 (www.perseus-framework.org)^53^. Intensities in each TMT reporter channel were log_2_ transformed and subtracted by median for normalization. Phosphoproteome data were further normalized by the median of proteomics data. To pick up phosphopeptides with significant differences, volcano plots were depicted based on minus log_10_ transformed *p* values from two-tailed Welch *t*-test, and log_2_ transformed fold changes (Log_2_ FC). Phosphopeptides with statistical significance (Log_2_ FC > 1 and *p* < 0.05) were subjected to a prediction of kinase activity score with KEA2.0^26^.

### Phospho-RTK array

Cells were seeded at a density of 3 × 10^6^ cells in a collagen-coated dish and cultured for 24 h. The cells were treated with DMSO (control) or the indicated concentration of drugs. Next, cell lysates were collected and applied to a phospho-RTK array assay using the Human Phospho-RTK Array Kit (R&D Systems, Minneapolis, MN, USA) according to the manufacturer’s instructions. The signal was detected using Amersham ImageQuant 800 (GE Healthcare).

### Quantitative RT-PCR

RNA was extracted from the cells using the RNeasy Mini Kit (QIAGEN, Hilden, Germany) according to the manufacturer’s instructions. cDNA was synthesized from the extracted RNA using Transcriptor First Strand cDNA Synthesis Kit (Roche, Basel, Switzerland) according to the protocol described in the kit. The synthesized cDNA was used for the template and mixed with FastStart Essential DNA Green Master kit (Roche) and target-specific primers, whose sequences were shown in Supplementary Table 2. Real-time PCR was performed using LightCycler 96 (Roche). GAPDH was used for control and the relative expression level of each gene was calculated as 2-ΔΔCt.

### Droplet digital PCR

cDNA was synthesized in the same methods described in the Quantitative RT-PCR section.

cDNA and EML4-ALK specific probes ddPCR EXD Assay EML4-ALK (Bio-Rad Laboratories, dHsaEXD86850342, Hercules, CA, USA) were mixed with ddPCR Supermix for Probe (no dUTP) (Bio-Rad Laboratories). Droplet was generated from the cDNA and probe mixture using Droplet Generator (Bio-Rad Laboratories) according to the manufacturer’s instructions. PCR was performed using C1000 Touch thermal cycler (Bio-Rad Laboratories) with the cycle described in the manufacturer’s protocol of the probes. After PCR, measurement of droplet was performed using Droplet Reader (Bio-Rad Laboratories) and QuantaSoft Analysis Pro Software (Bio-Rad Laboratories) was used for analysis.

### Statistical analysis

GraphPad Prism version 9.1.2 (GraphPad Software) was used to perform statistical analysis and graphically display the data. Statistical methods used in each analysis are shown in figure legends and statistical significance was accepted for *p* values < 0.05.

### Reporting summary

Further information on research design is available in the Nature Research Reporting Summary linked to this article.

## Supplementary information

Supplementary Figure 1-37 and Supplementary Table 1-2

Supplementary Data set 1

REPORTING SUMMARY

## Data Availability

We have deposited the phosphoproteomics data in this study to the jPOSTrepo; JPST001314 for jPOST and PXD028445 for ProteomeXchange^54^. All the other data supporting the findings of this study are available within the article and its Supplementary Information files and from the corresponding authors upon reasonable request.

## Code availability

No code or scripts are used in this study.
