# Supplementary material for: GSK3 inhibition circumvents and overcomes acquired lorlatinib resistance in ALK-rearranged non-small-cell lung cancer
Source: NPJ Precis Oncol. 2022 Mar 17;6:16. doi: 10.1038/s41698-022-00260-0 (PMC8931094; doi:10.1038/s41698-022-00260-0)
Supplement: Supplementary file 1 — Supplementary Figure 1-37 and Supplementary Table 1-2 [file 41698_2022_260_MOESM1_ESM.pdf]

Supplementary Figure 1

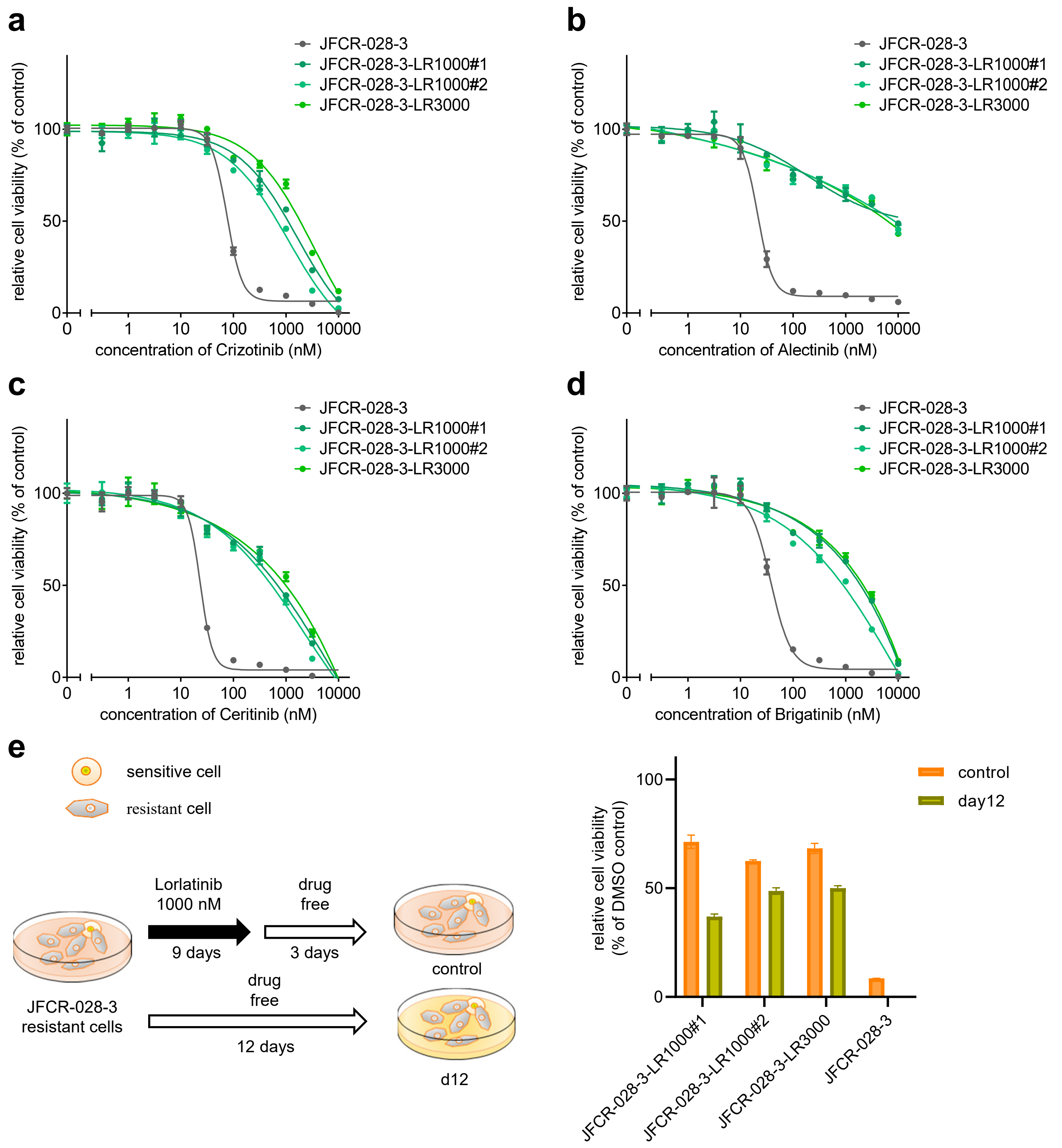

Supplementary Figure 1. Lorlatinib intermediate resistant cells derived from JFCR-028-3 were intermediate resistant to multiple ALK-TKIs.

(a-d) JFCR-028-3 parental cells and the lorlatinib intermediate resistant cells were treated with the indicated concentration of crizotinib (a), alectinib (b), ceritinib (c) and brigatinib (d) for 72 h. Cell viability was measured using the CellTiter-Glo assay (*n* = 3). Each points represent mean ± SD of three replicates. (e) JFCR-028-3 parental cells, JFCR-028-3 lorlatinib intermediate resistant cellls, and JFCR-028-3 lorlatinib intermediate resistant cells-d12 were treated with the indicated concentration of lorlatinib for 72 h. Cell viability was measured using the CellTiter-Glo assay (*n* = 3). Each points represent mean ± SD of three replicates.

Supplementary Figure 2

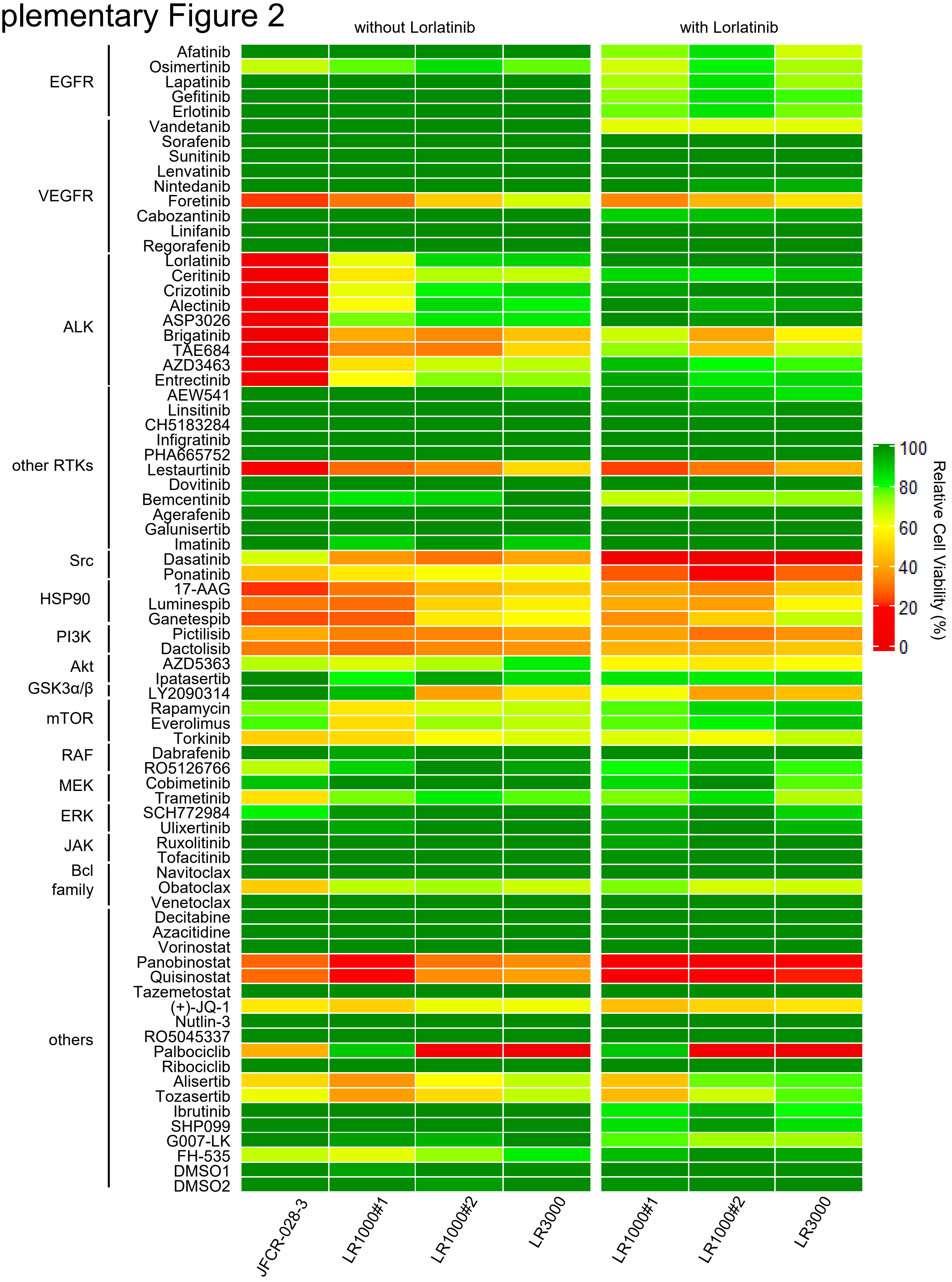

Supplementary Figure 2. A focused inhibitor library screening revealed that GSK3 inhibitors suppressed cell viability of the lorlatinib intermediate resistant cells specifically.

Inhibitor library screening was performed as a single agent and in the presence of 100 nM of lorlatinib. JFCR-028-3 parental cells and the lorlatinib intermediate resistant cells were treated with each inhibitor with or without 100 nM of lorlatinib for 72 h. Cell viability was measured using the CellTiter-Glo assay ($n = 2$). Relative cell viability was calculated from each value divided by DMSO control.

Supplementary Figure 3

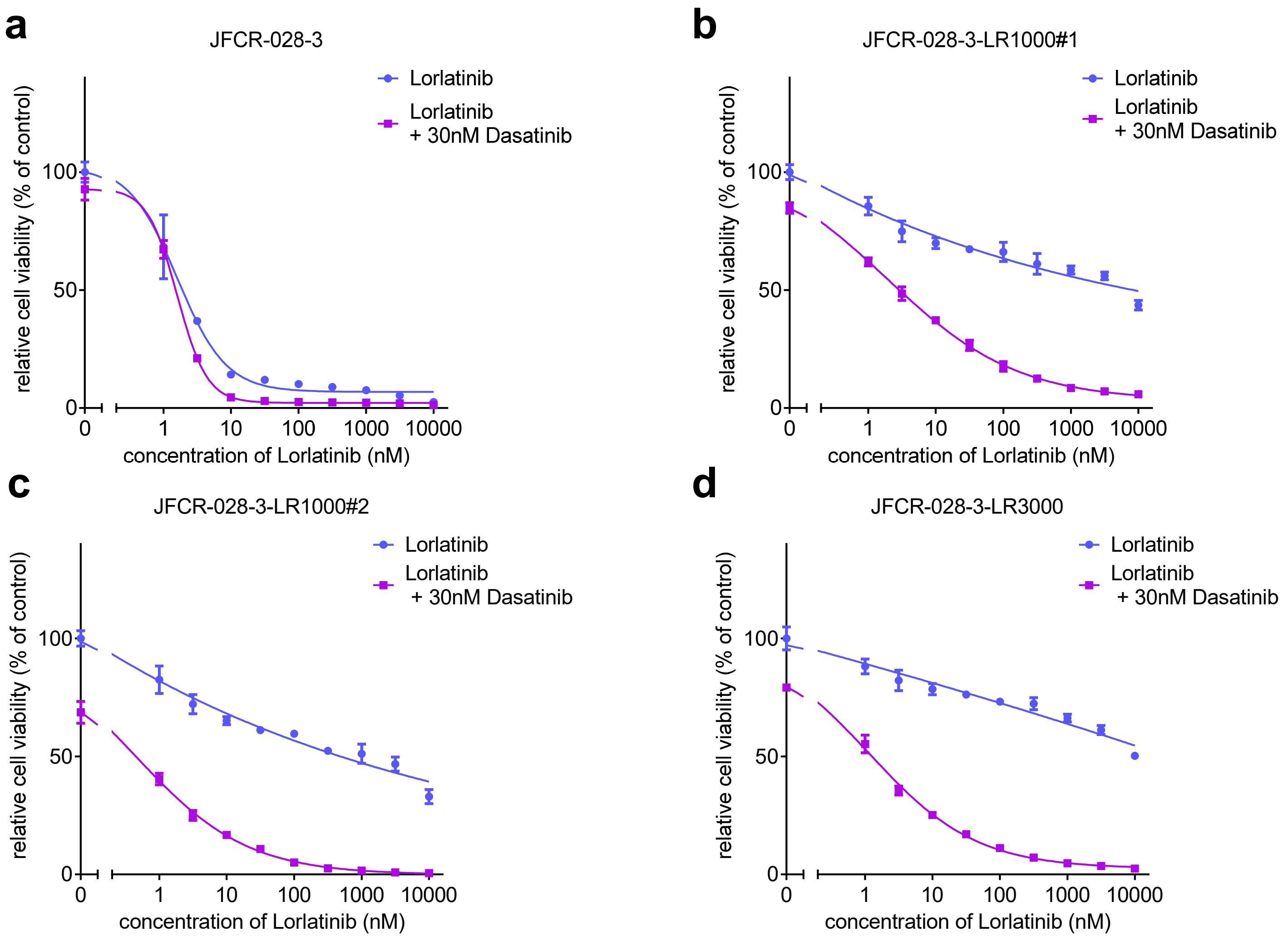

Supplementary Figure 3. A potent Src family kinase inhibitor, dasatinib, showed more strongly growth suppression in the lorlatinib intermediate resistant cells in combination with lorlatinib .

(a-d) JFCR-028-3 parental cells (a), and the lorlatinib intermediate resistant cells (b-d) were treated with the indicated concentration of lorlatinib in the presence or absence of a fixed concentration of dasatinib for 72 h. Cell viability was measured using the CellTiter-Glo assay (*n* = 3). Each points represent mean ± SD of three replicates.

Supplementary Figure 4

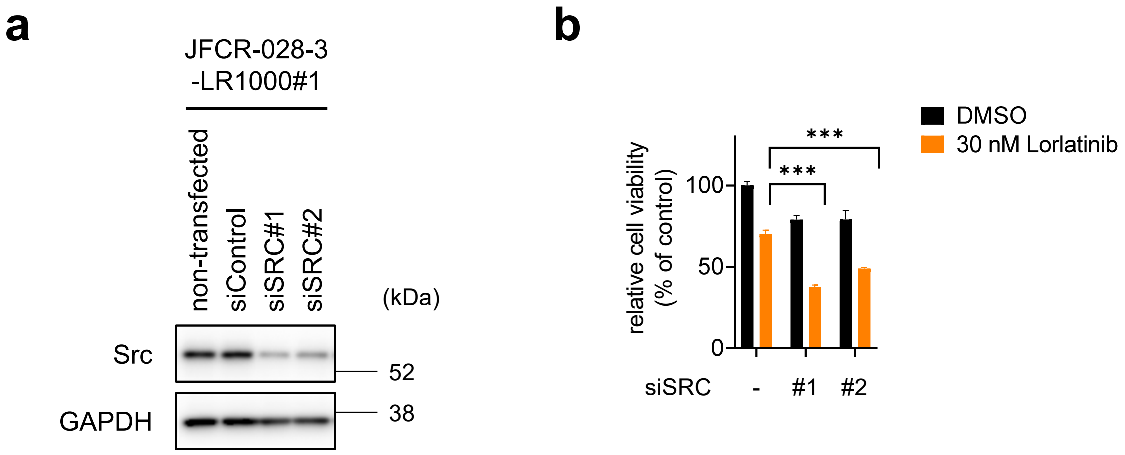

Supplementary Figure 4. Silencing Src enhanced the sensitivity against lorlatinib in the lorlatinib intermediate resistant cells

(a) JFCR-028-3-LR1000#1 cells were treated with siRNA for 48 h. GAPDH was used as a loading control. (b) Cell viability of JFCR-028-3-LR1000#1 cells treated with siRNA for 96 h with or without 30 nM lorlatinib for 72 h. Cell viability was measured using the CellTiter-Glo assay (n = 3). Each bars represent mean ± SD of three replicates. Statistical significance was calculated by two-tailed Welch's t test, and *** indicates $p < 0.001$.

Supplementary Figure 5

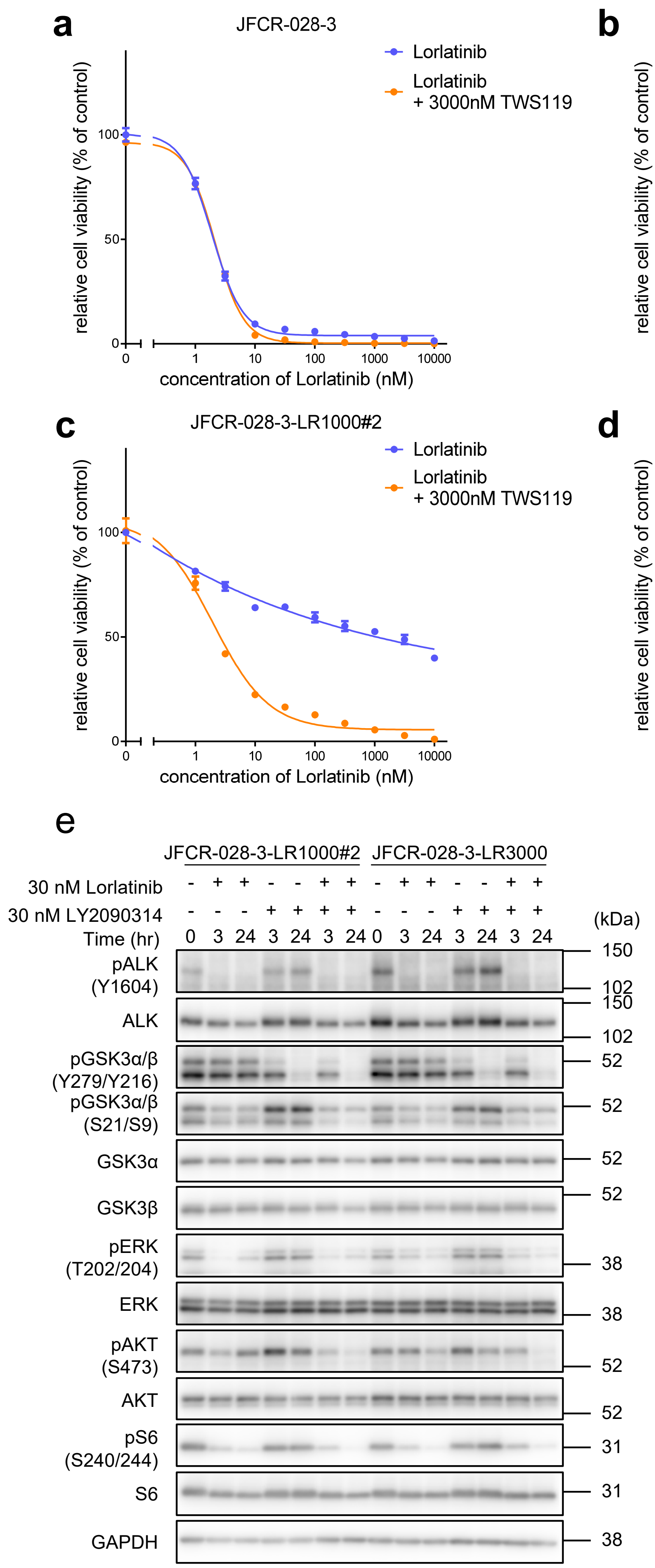

Supplementary Figure 5. The combined inhibition of lorlatinib and TWS119 showed the combinational effect in the lorlatinib intermediate resistant cells. (a-d) JFCR-028-3 parental cells (a), and the lorlatinib intermediate resistant cells (b-d) were treated with the indicated concentration of lorlatinib in the presence or absence of a fixed concentration of TWS119 for 72 h. Cell viability was measured using the CellTiter-Glo assay (n = 3). Each points represent mean ± SD of three replicates. (e) immunoblot analysis of the indicated proteins. JFCR-028-3-LR1000#2 and JFCR-028-3-LR3000 lorlatinib intermediate resistant cells were treated with the indicated concentrations of lorlatinib in the absence or presence of LY2090314 for 0, 3 and 24 h. GAPDH was used as a loading control.

Supplementary Figure 6

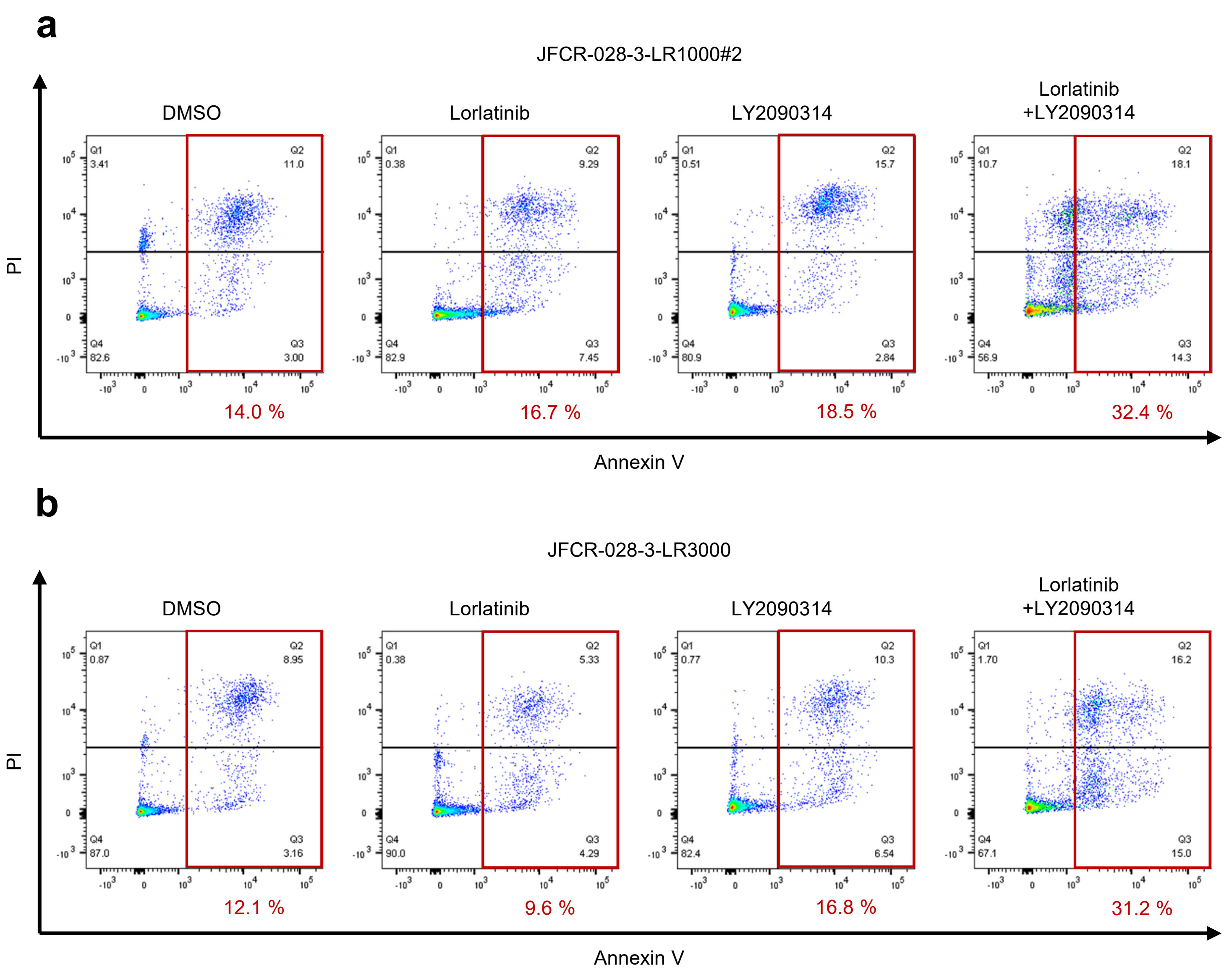

Supplementary Figure 6. The combination of lorlatinib and LY2090314 induced apoptosis in the lorlatinib intermediate resistant cells.

(a-b) JFCR-028-3-LR1000#2 cells (a), and JFCR-028-3-LR3000 cells (b) were treated with single treatment or combination of 30 nM lorlatinib and 100 nM LY2090314. Apoptosis was evaluated using Annexin-V and propidium iodide (PI) staining after 72 h of the indicated drug treatment. The apoptotic cells were shown in red square and the percentage of apoptotic cells is shown in red value.

Supplementary Figure 7

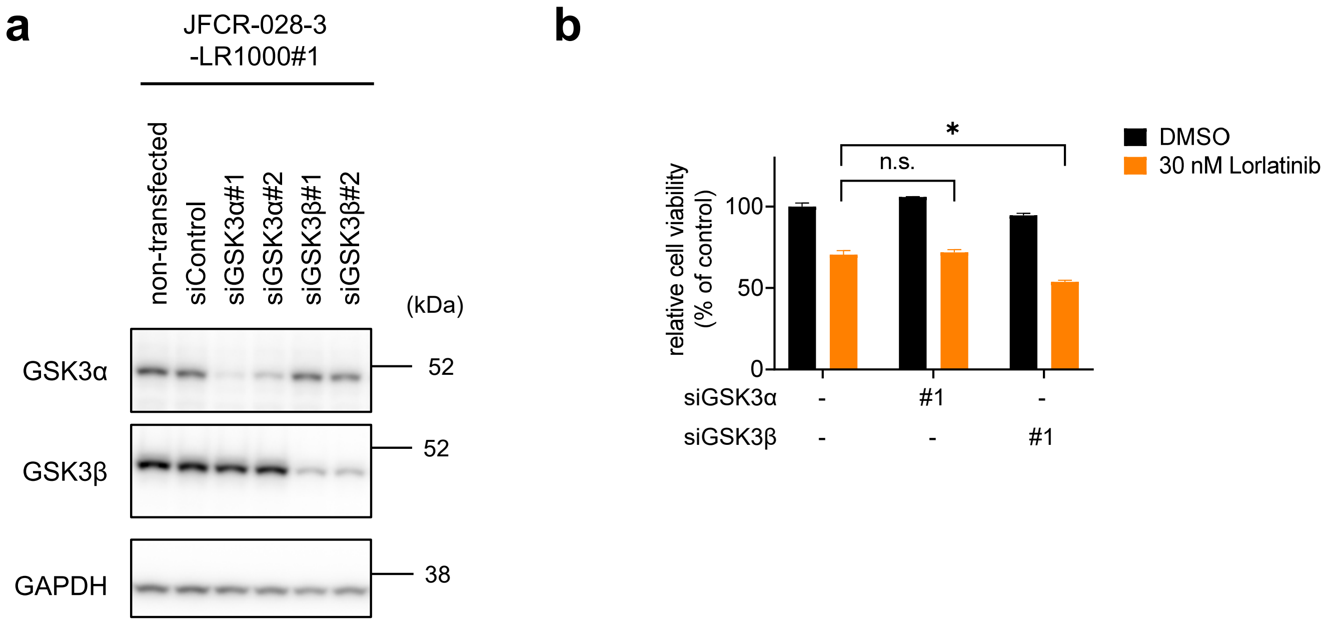

Supplementary Figure 7. Silencing GSK3β partially enhanced the sensitivity against lorlatinib in the lorlatinib intermediate resistant cells

(a) JFCR-028-3-LR1000#1 cells were treated with siRNA for 48 h. GAPDH was used as a loading control. (b) Cell viability of JFCR-028-3-LR1000#1 cells treated with siRNA for 96 h with or without 30 nM lorlatinib for 72 h. Cell viability was measured using the CellTiter-Glo assay (n = 3). Each bars represent mean ± SD of three replicates. Statistical significance was calculated by two-tailed Welch's t test, and * indicates $p < 0.05$.

Supplementary Figure 8

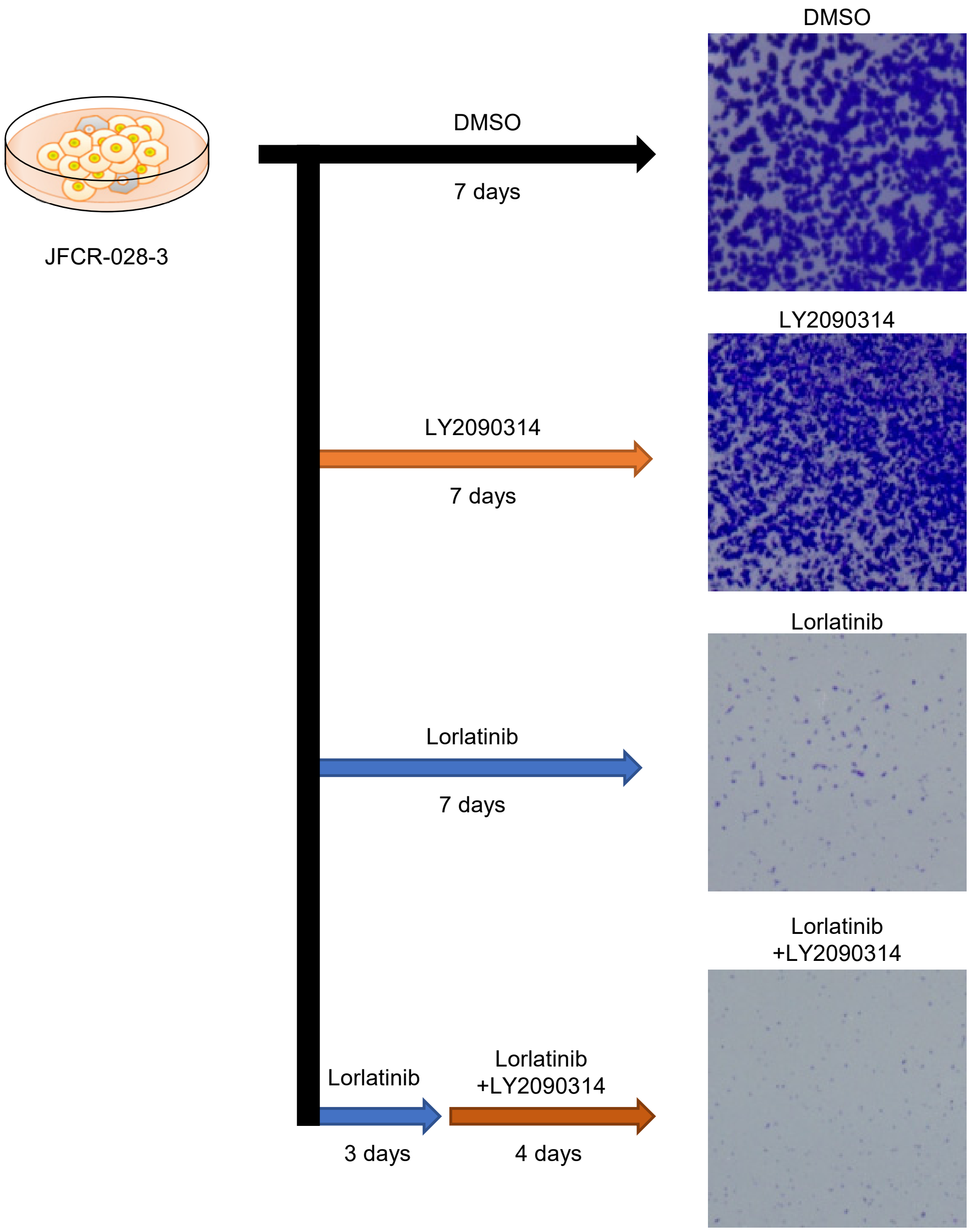

Supplementary Figure 8. The combination with LY2090314 diminished the remained resistant clones more dramatically.

JFCR-028-3 parental cells were treated with 1000 nM lorlatinib and 100 nM LY2090314. In the combination setting, the cells were treated with 1000 nM lorlatinib and 100 nM LY2090314 after 3 days of 1000 nM lorlatinib single treatment. Following 7 days of drug treatment, the cells were stained using crystal violet.

Supplementary Figure 9

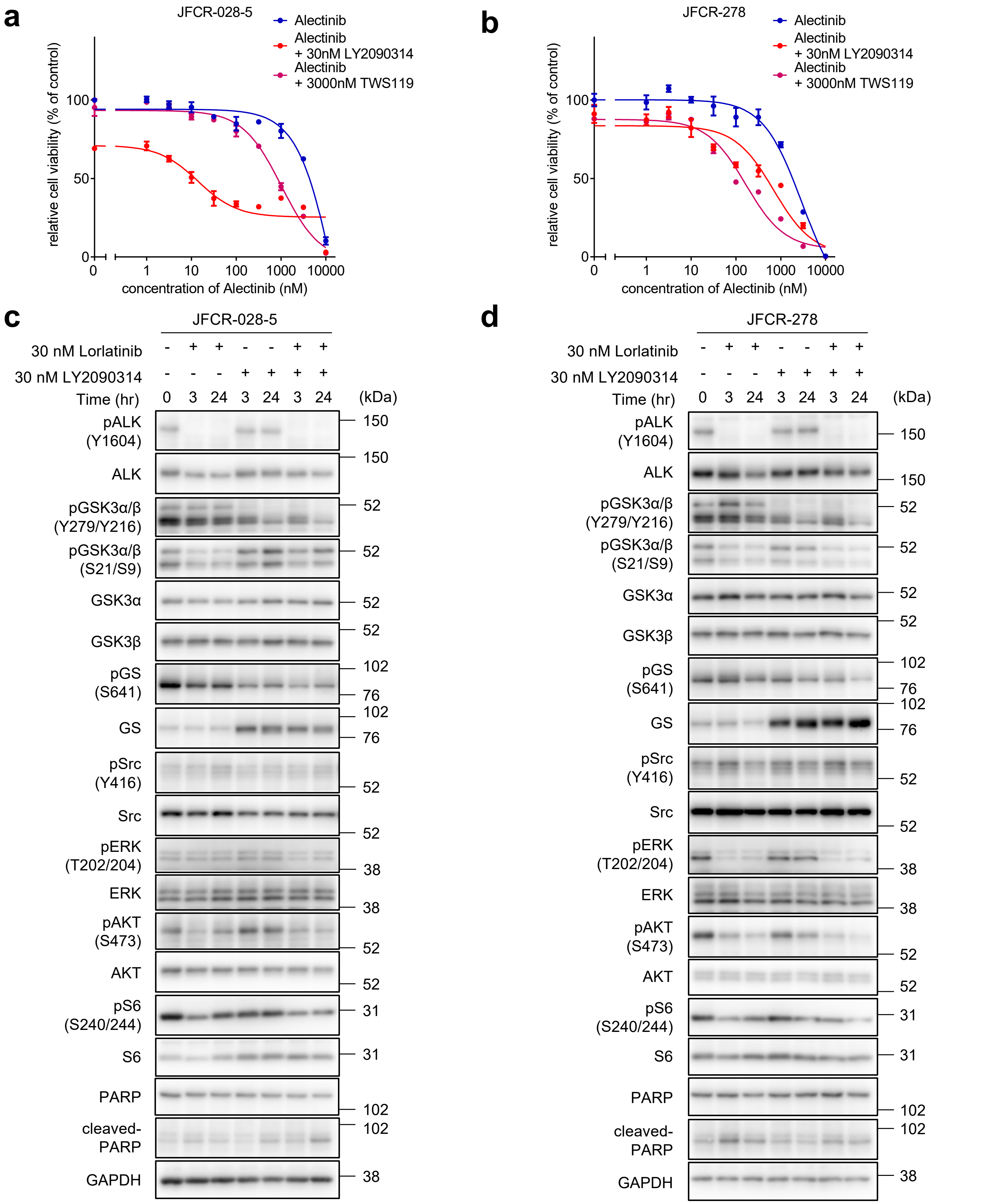

Supplementary Figure 9. GSK3 inhibition induced re-sensitization of alectinib in alectinib-failure patient-derived cells. (a and b) JFCR-028-5 cells (a) and JFCR-278 cells (b) were treated with the indicated concentration of alectinib in the presence or absence of a fixed concentration of the GSK3 inhibitors for 72 h. Cell viability was measured using the CellTiter-Glo assay ($n = 3$). Each points represent mean ± SD of three replicates. (c and d) immunoblot analysis of the indicated proteins. JFCR-028-5 cells (c) and JFCR-278 cells (d) cells were treated with the indicated concentrations of lorlatinib in the absence or presence of LY2090314 for 0, 3 and 24 h. GAPDH was used as a loading control.

Supplementary Figure 10

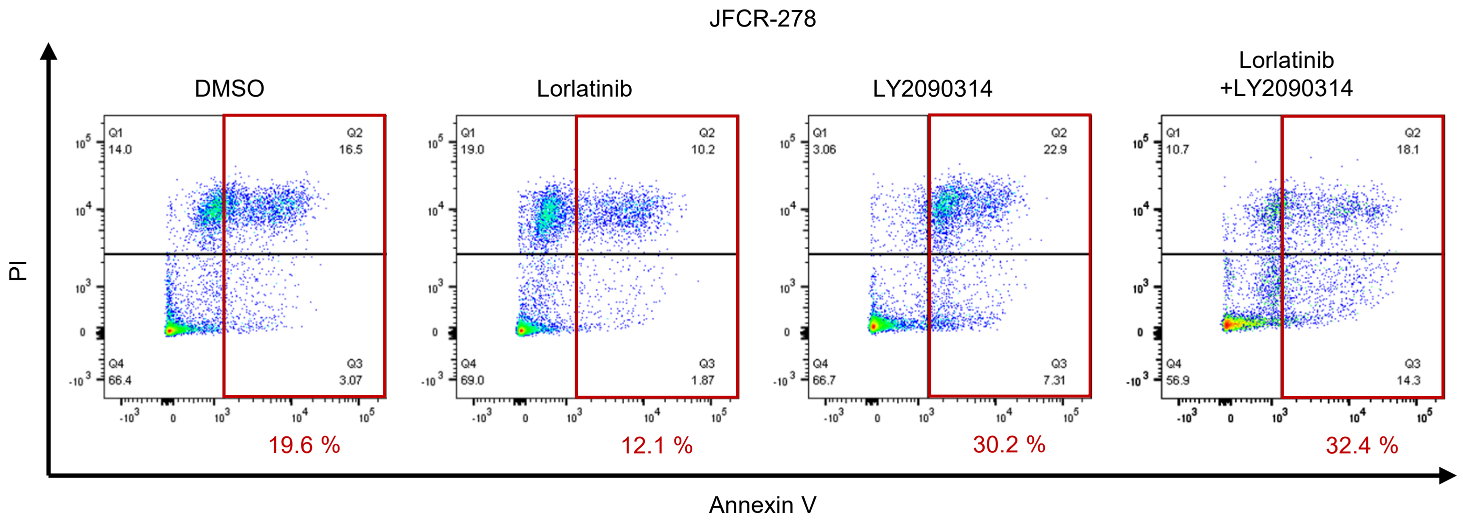

Supplementary Figure 10. The combination of lorlatinib and LY2090314 induced apoptosis in alectinib resistant PDC JFCR-278 cells.

JFCR-278 cells were treated with single treatment or combination of 30 nM lorlatinib and 100 nM LY2090314. Apoptosis was evaluated using Annexin-V and propidium iodide (PI) staining after 72 h of the indicated drug treatment. The apoptotic cells were shown in red square and the percentage of apoptotic cells is shown in red value.

Supplementary Figure 11

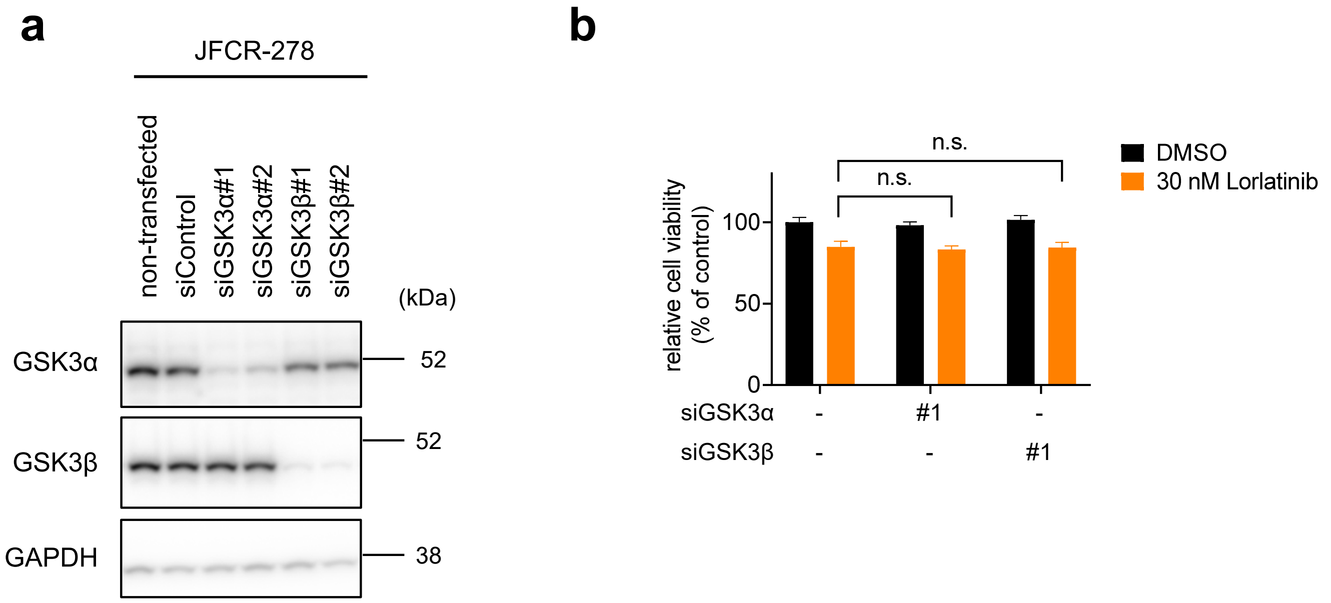

Supplementary Figure 11. Silencing GSK3β did not enhance the sensitivity against lorlatinib in alectinib resistant PDC JFCR-278 cells.

(a) JFCR-278 cells were treated with siRNA for 48 h. GAPDH was used as a loading control. (b) Cell viability of JFCR-278 cells treated with siRNA for 96 h with or without 30 nM lorlatinib for 72 h. Cell viability was measured using the CellTiter-Glo assay (n = 3). Each bars represent mean ± SD of three replicates. Statistical significance was calculated by two-tailed Welch's t test, and n.s. indicates not significant.

Supplementary Figure 12

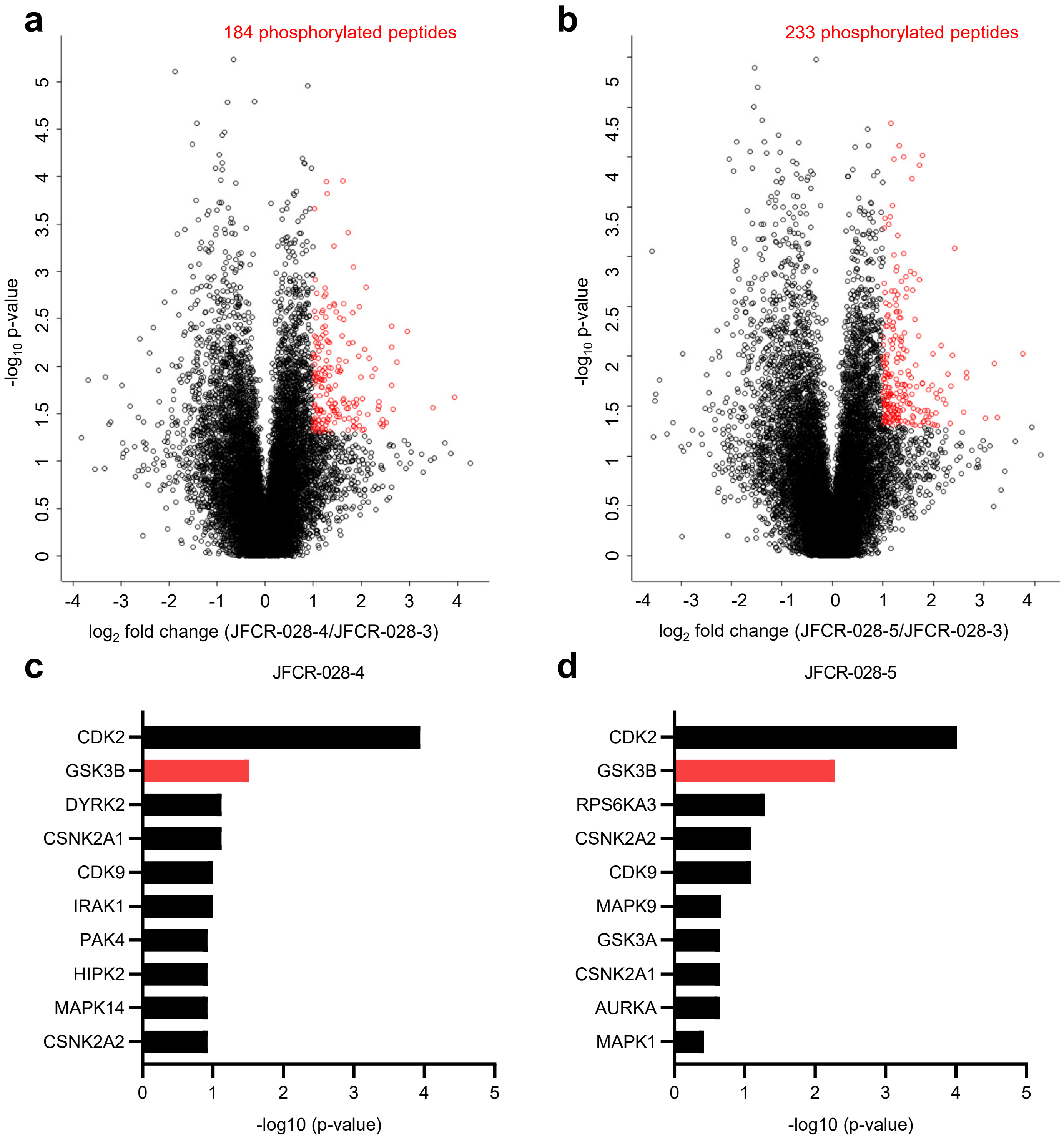

Supplementary Figure 12. GSK3 were enriched in alectinib-failure patient-derived cells compared with alectinib naïve cells.

(a and b) Volcano plot exhibited expression changes in phosphorylated peptides. These plots indicate the log2 fold change between upregulated or downregulated phosphorylated peptides in JFCR-028-4 (a) or JFCR-028-5 (b) compared with JFCR-028-3. Each plot is based on the median fold-change of value and the p-value of three replicated samples. The phosphorylated peptides which showed significant increase of more than two-fold change (p-value < 0.05) are plotted in red. Statistical significance was calculated by a student's t-test (c and d) Kinase enrichment analysis was performed using the phosphorylation proteins differentially expressed in JFCR-028-4 (c) and JFCR-028-5 (d) compared with JFCR-028-3. The top ten enrichment proteins as upstream regulators are presented. The p-value represents corrected p-value with Benjamini-Hochberg calculated based on the method of KEA2 web application.

Supplementary Figure 13

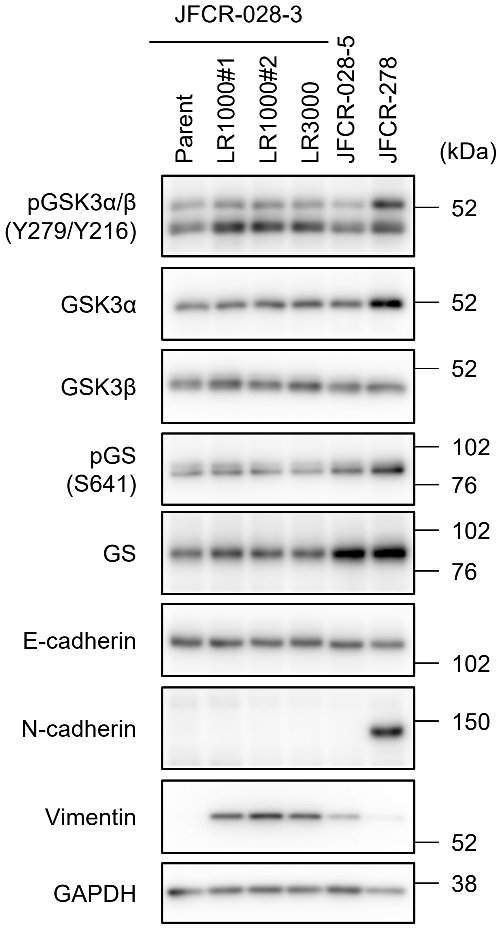

Supplementary Figure 13. The phosphorylation of GSK3 were activated in the lorlatinib intermediate resistant cells and the acquired resistant patient-derived cells compared with alectinib naïve cells.

The phosphorylation and the protein expression in JFCR-028-3 parental cells, the lorlatinib intermediate resistant cells and the acquired resistant patient-derived cells were evaluated using western blot analysis. GAPDH was used as a loading control.

Supplementary Figure 14

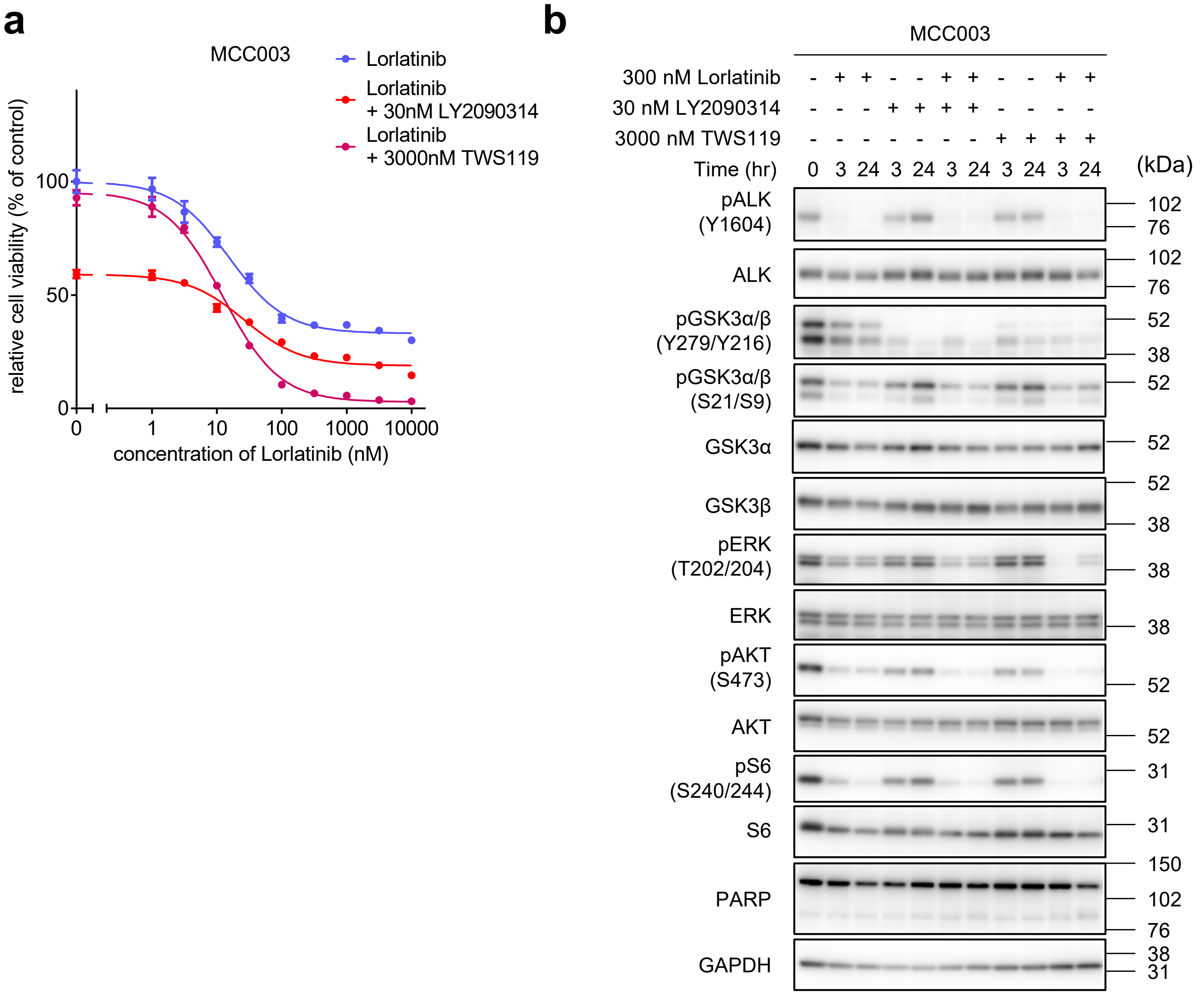

Supplementary Figure 14. The combination of lorlatinib and GSK3 inhibitors more strongly suppressed cell viability in ALK mutation mediated resistant cells.

(a) MCC003 cells was treated with the indicated concentration of lorlatinib with or without a fixed concentration of the GSK3 inhibitors for 72 h. Cell viability was measured using the CellTiter-Glo assay (n = 3). Each points represent mean ± SD of three replicates. (b) The suppression of phospho-ALK, phospho-GSK3, and its downstream signaling in MCC003 cells was evaluated using western blot analysis. Cells were treated with 300 nM lorlatinib in the absence or presence of the indicated concentration of GSK3 inhibitors for 0, 3 and 24 h. GAPDH was used as a loading control.

Supplementary Figure 15

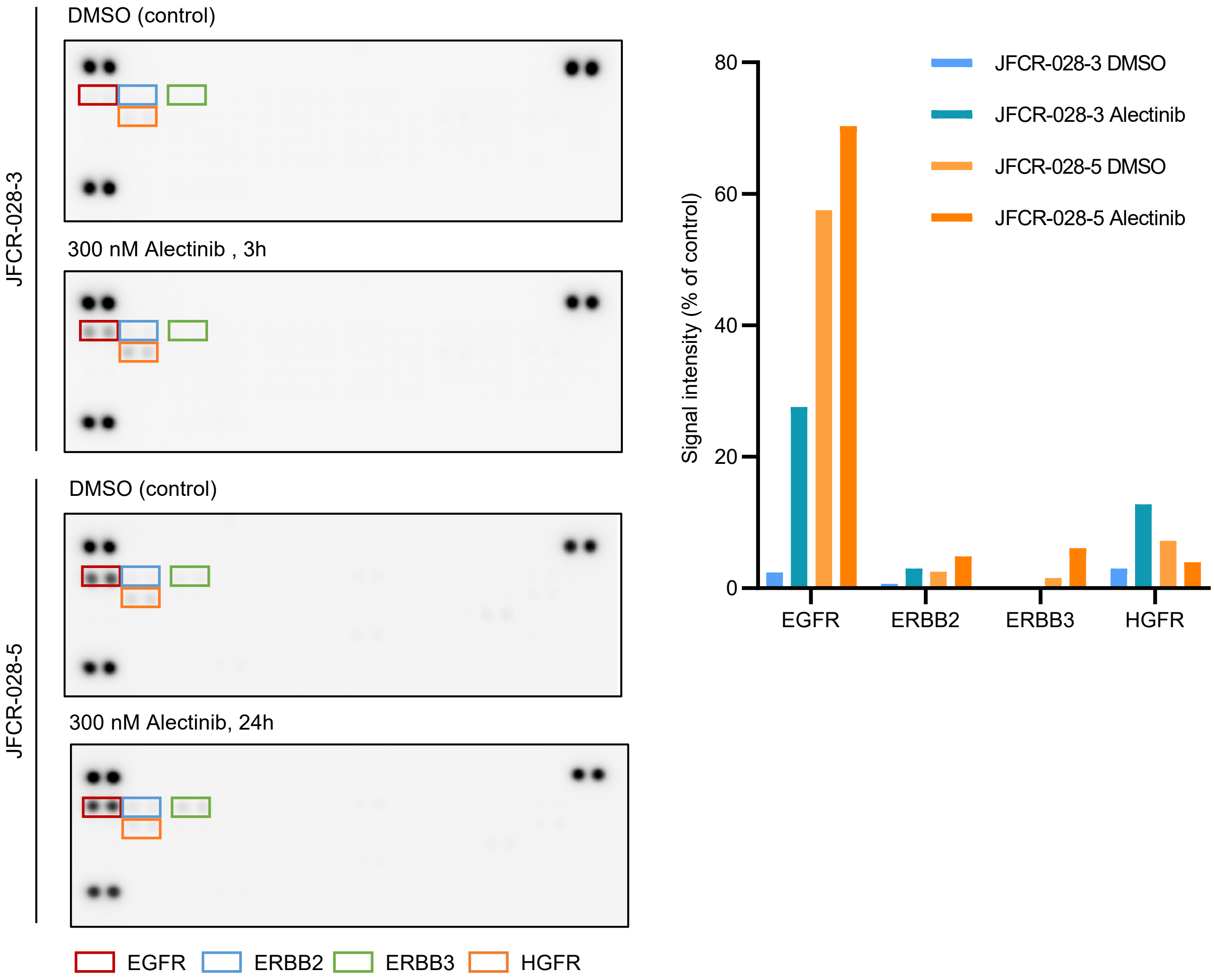

Supplementary Figure 15. The phosphorylation of EGFR was activated in ALK-TKI acquired resistant cell. Cells were treated in DMSO (control) or 300 nM alectinib for the indicated time, and lysates were incubated with phospho-RTK arrays. The positions of phospho-EGFR and phospho-HGFR are indicated. Quantification of signal intensity of each dots is shown in the bar plot in the right side. Dots were quantified using ImageJ software and signal intensity was calculated as percentage of control dots.

Supplementary Figure 16

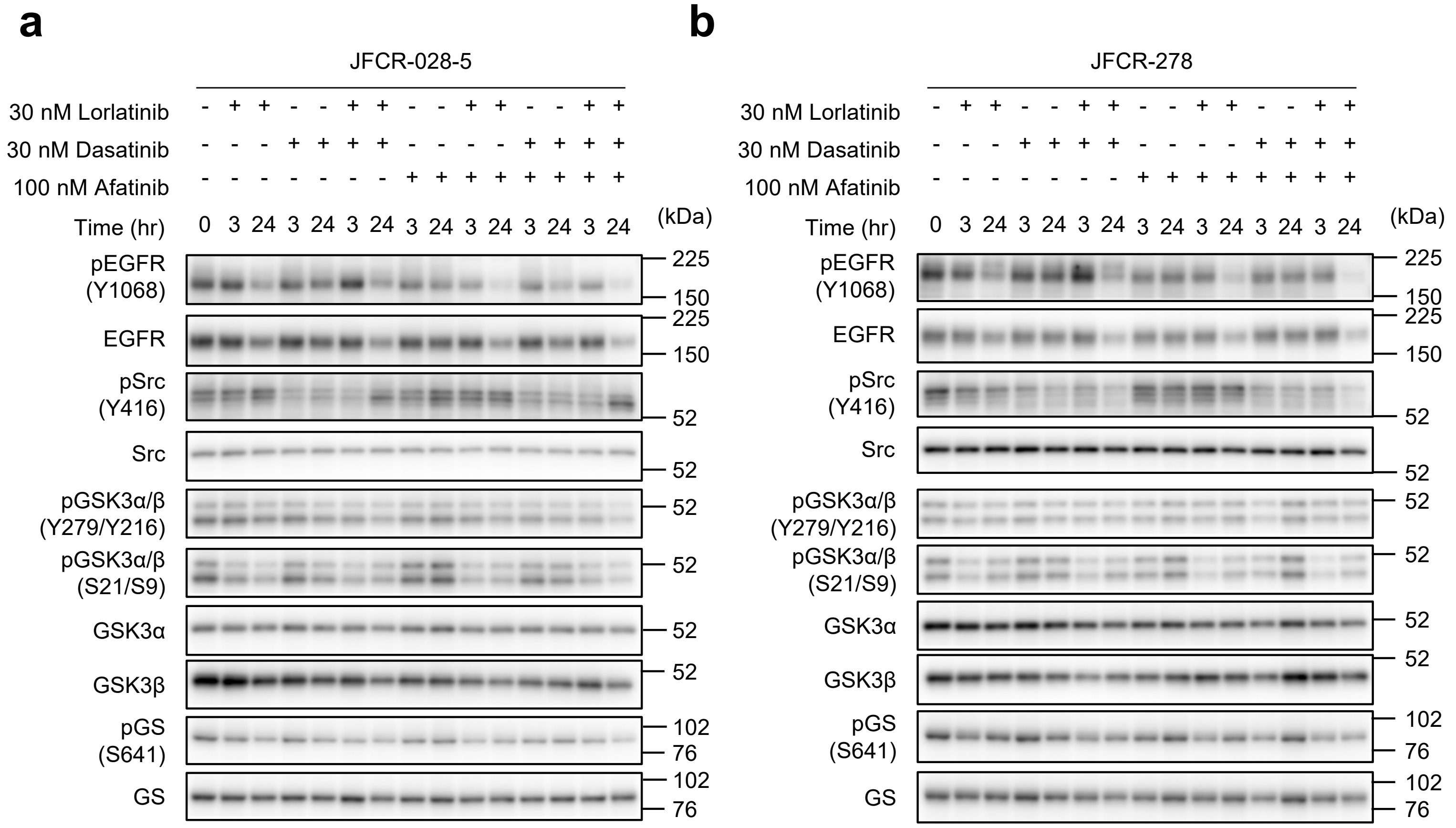

Supplementary Figure 16. Western blotting analysis of the combination therapy of lorlatinib, Src family kinase inhibitor, and EGFR inhibitor related in Figure 5C and 5D.

(a and b) The phosphorylation and total expression of EGFR, Src, GSK3α/β and GS in JFCR-028-5 cells (a) and JFCR-278 cells (b) cells was evaluated using western blot analysis. Cells were treated with 30 nM lorlatinib in the absence or presence of the indicated concentration of dasatinib or/and afatinib for 0, 3 and 24 h.

Supplementary Figure 17

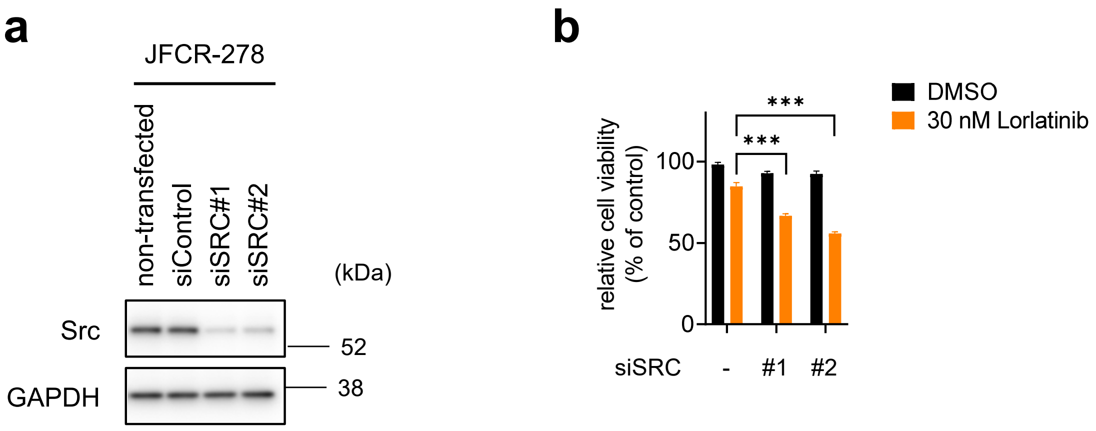

Supplementary Figure 17. Silencing Src enhanced the sensitivity against lorlatinib in JFCR-278.
(a) JFCR-278 cells were treated with siRNA for 48 h. GAPDH was used as a loading control. (b) Cell viability of JFCR-278 cells treated with siRNA for 96 h with or without 30 nM lorlatinib for 72 h. Cell viability was measured using the CellTiter-Glo assay (n = 3). Each bars represent mean ± SD of three replicates. Statistical significance was calculated by two-tailed Welch's t test, and *** indicates $p < 0.001$.

Supplementary Figure 18

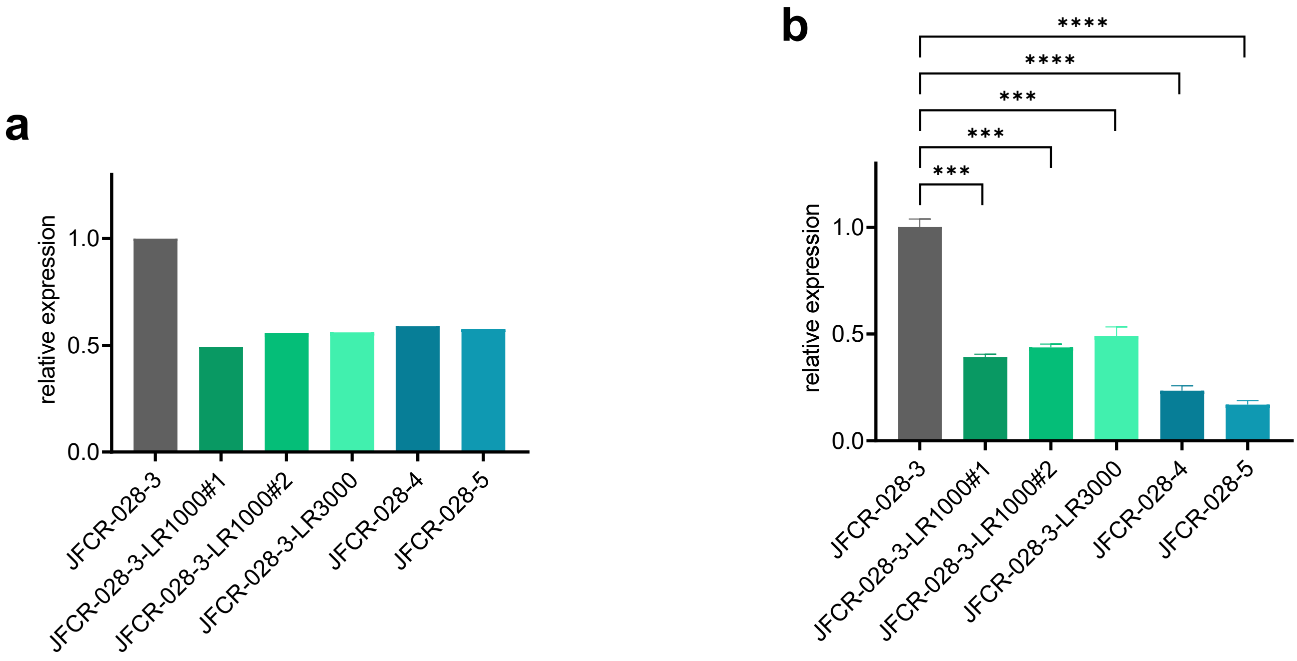

Supplementary Figure 18. The gene expression of EML4-ALK and EML4 was down-regulated in in the lorlatinib intermediate resistant cells and acquired resistant cells compared with JFCR-028-3 parental cells.
(a) The expression of EML4-ALK was detected in JFCR-028-3 parental cells, the lorlatinib intermediate resistant cells and the acquired resistant cells by droplet digital PCR using EML4-ALK fusion genes specific primers.
(b) Analyzing gene expression of EML4 in JFCR-028-3 parental cells, the lorlatinib intermediate resistant cells and the acquired resistant cells by using qPCR. Each data was represent as mean ± SD of three replicates. GAPDH was used as a loading control. Statistical significance was calculated by two-tailed t test, and *** indicates $p < 0.001$ and **** indicates $p < 0.001$.

Supplementary Figure 19

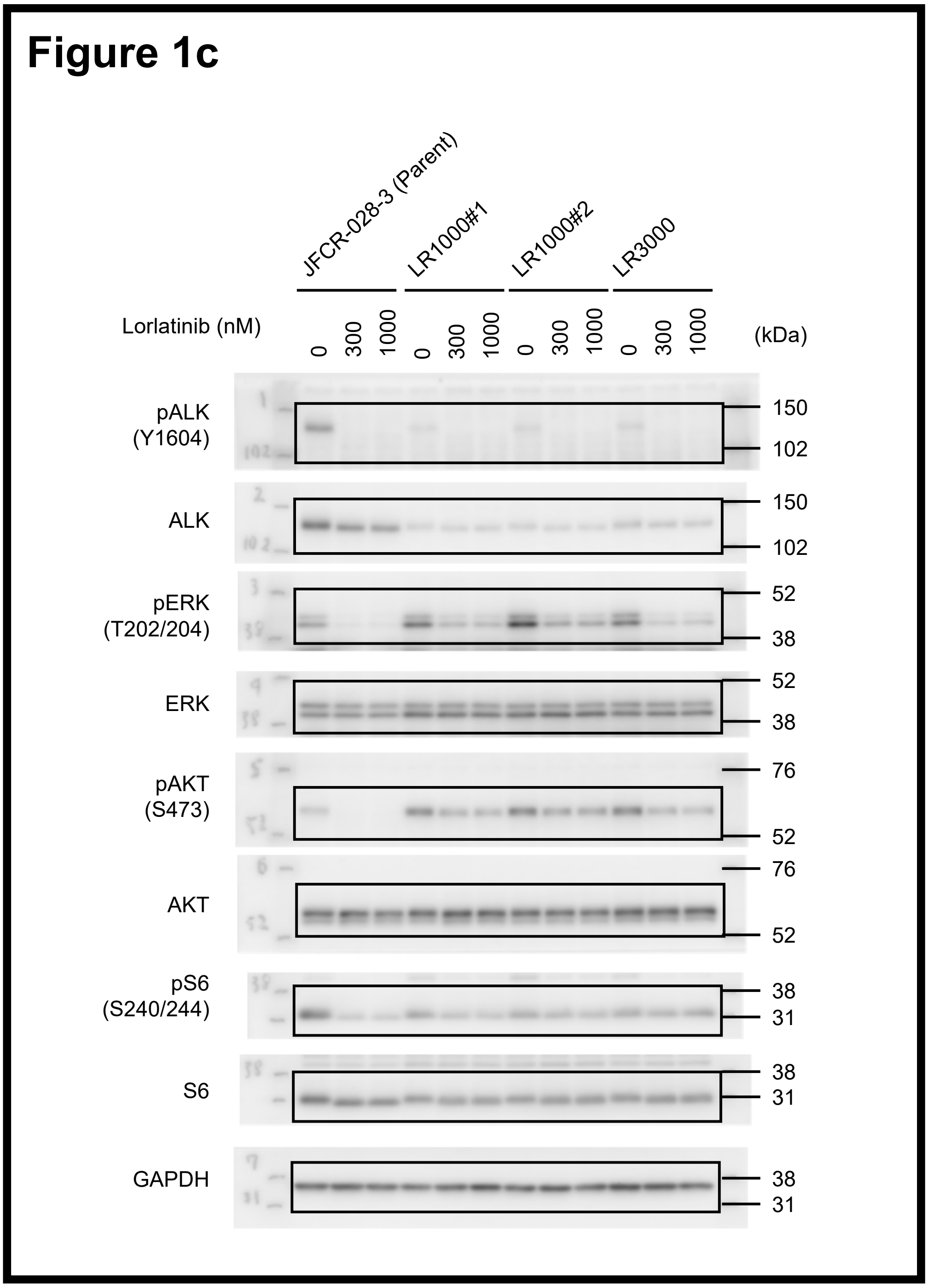

Supplementary Figure 19. Original data of immunoblot analysis for indicated figure.

Supplementary Figure 20

a

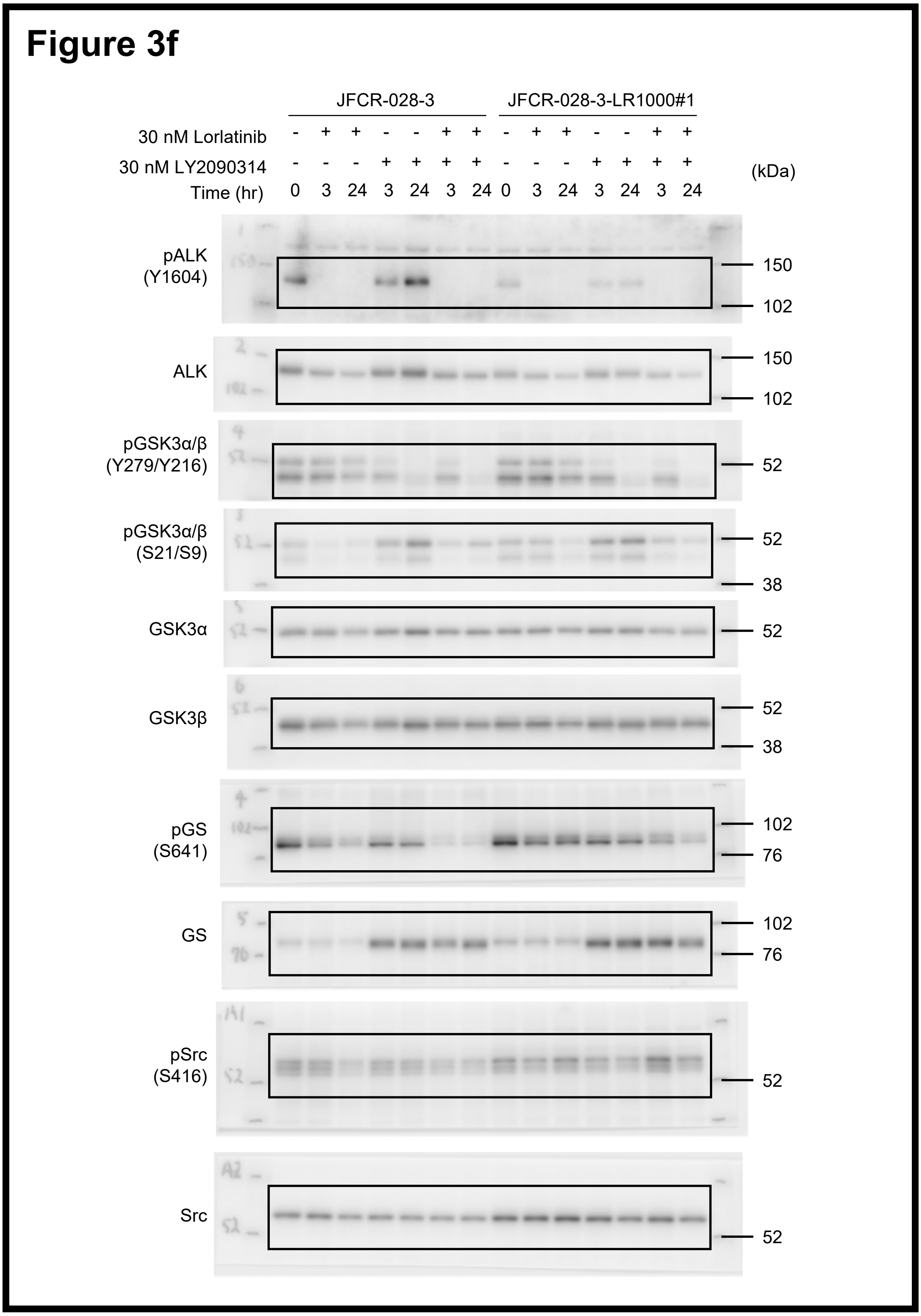

Supplementary Figure 20. Original data of immunoblot analysis for indicated figure.

Supplementary Figure 20

b

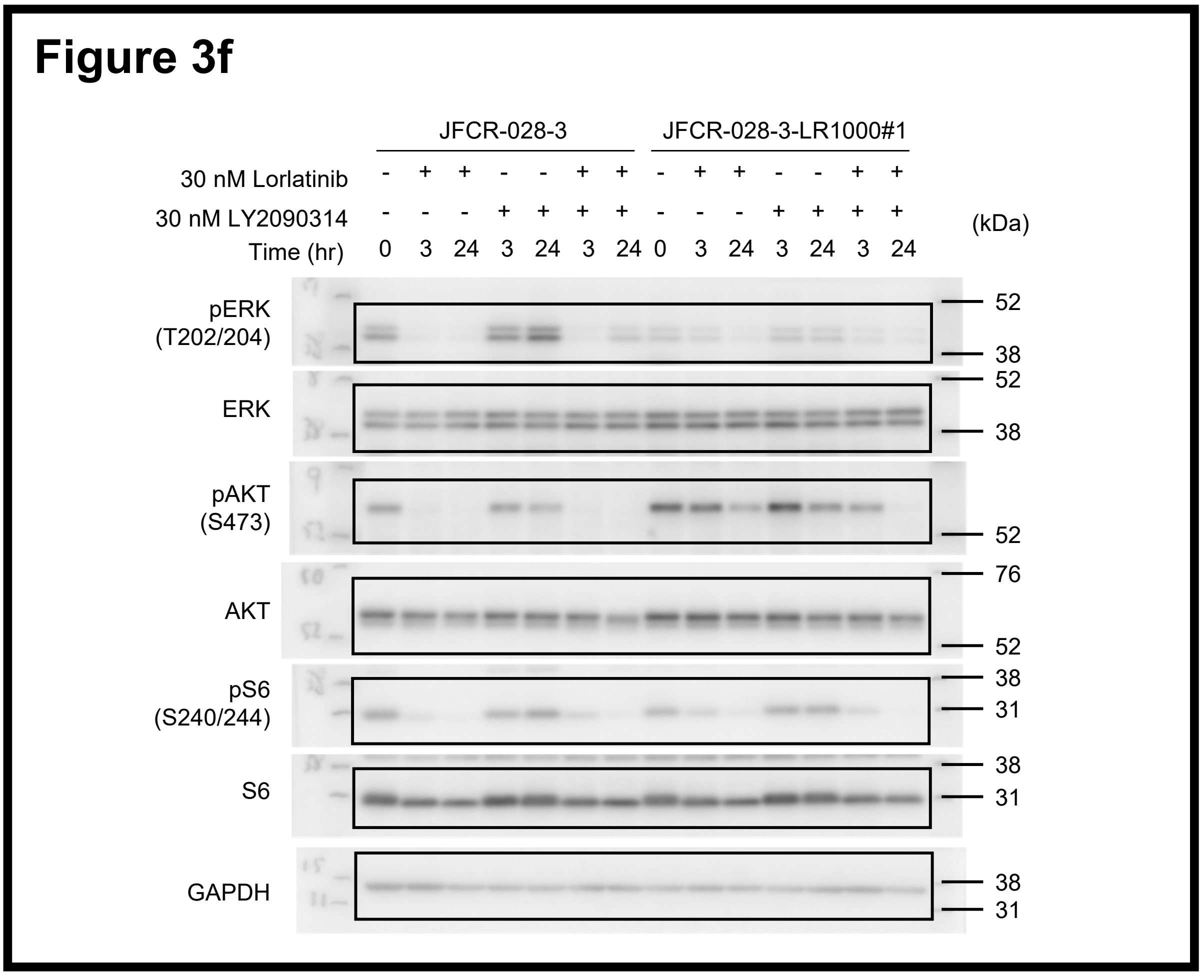

Supplementary Figure 20. Original data of immunoblot analysis for indicated figure.

Supplementary Figure 21

Figure 7c

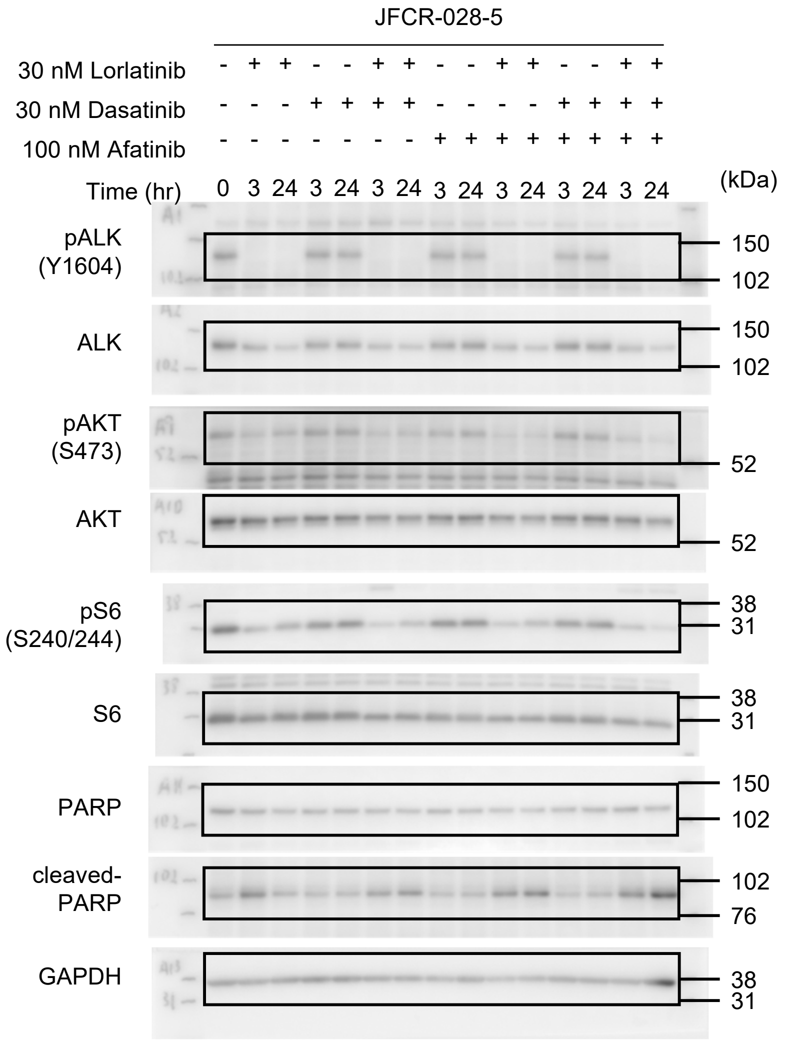

Supplementary Figure 21. Original data of immunoblot analysis for indicated figures.

Supplementary Figure 22

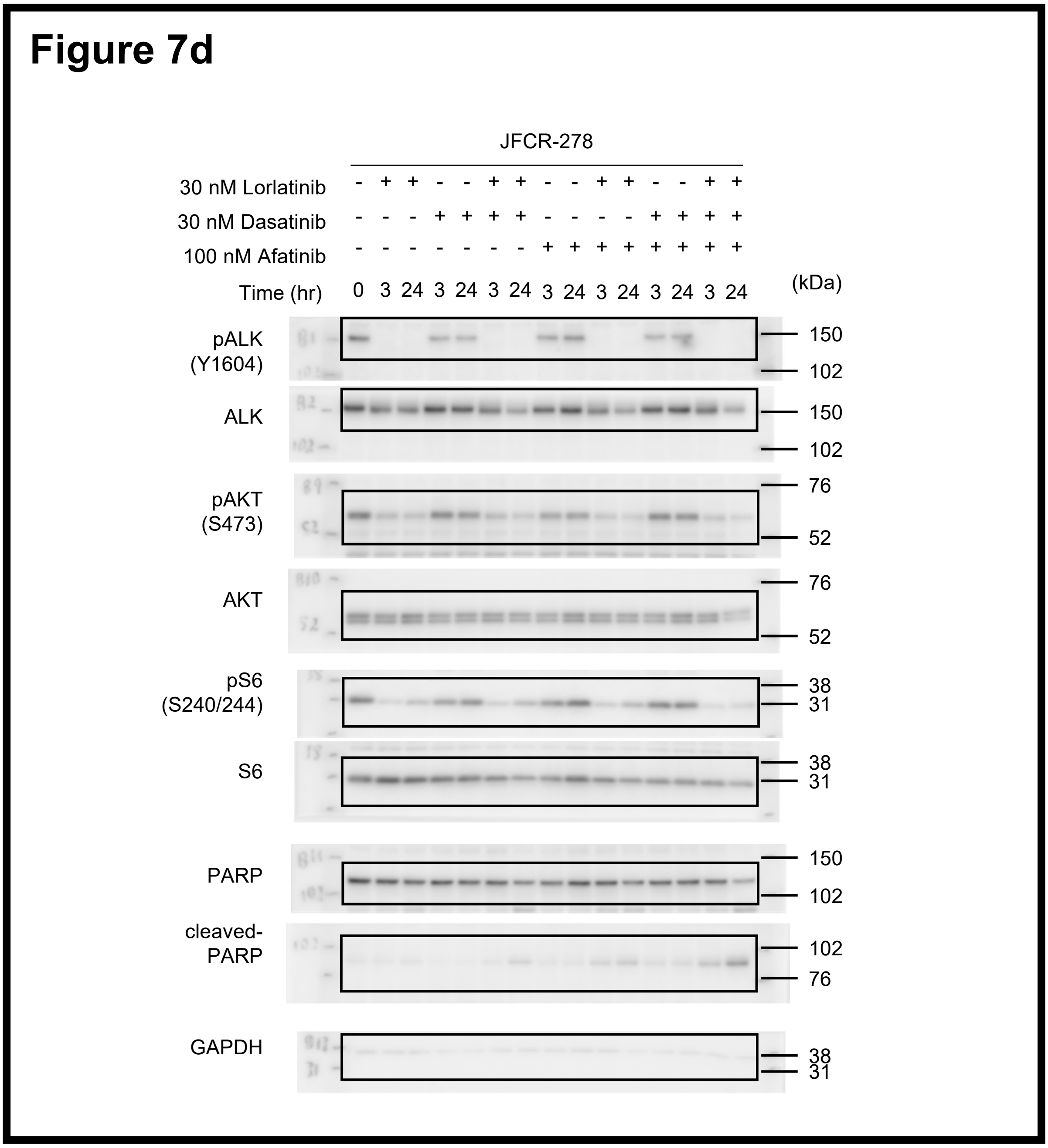

Supplementary Figure 22. Original data of immunoblot analysis for indicated figures.

Supplementary Figure 23

Supplementary Figure 4a

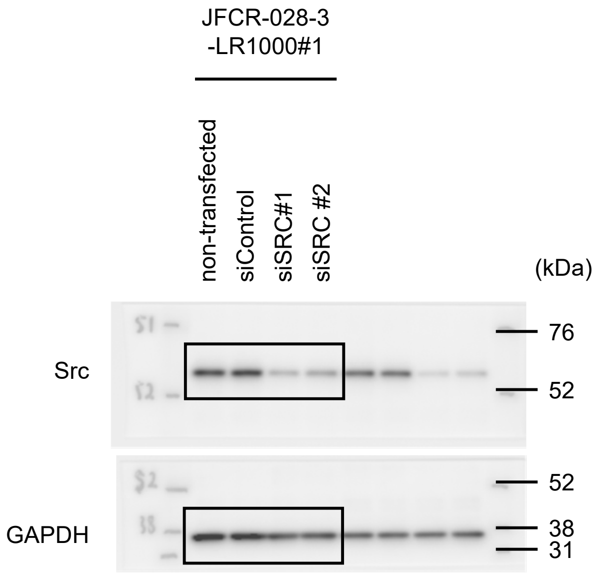

Supplementary Figure 23. Original data of immunoblot analysis for indicated figures.

Supplementary Figure 24

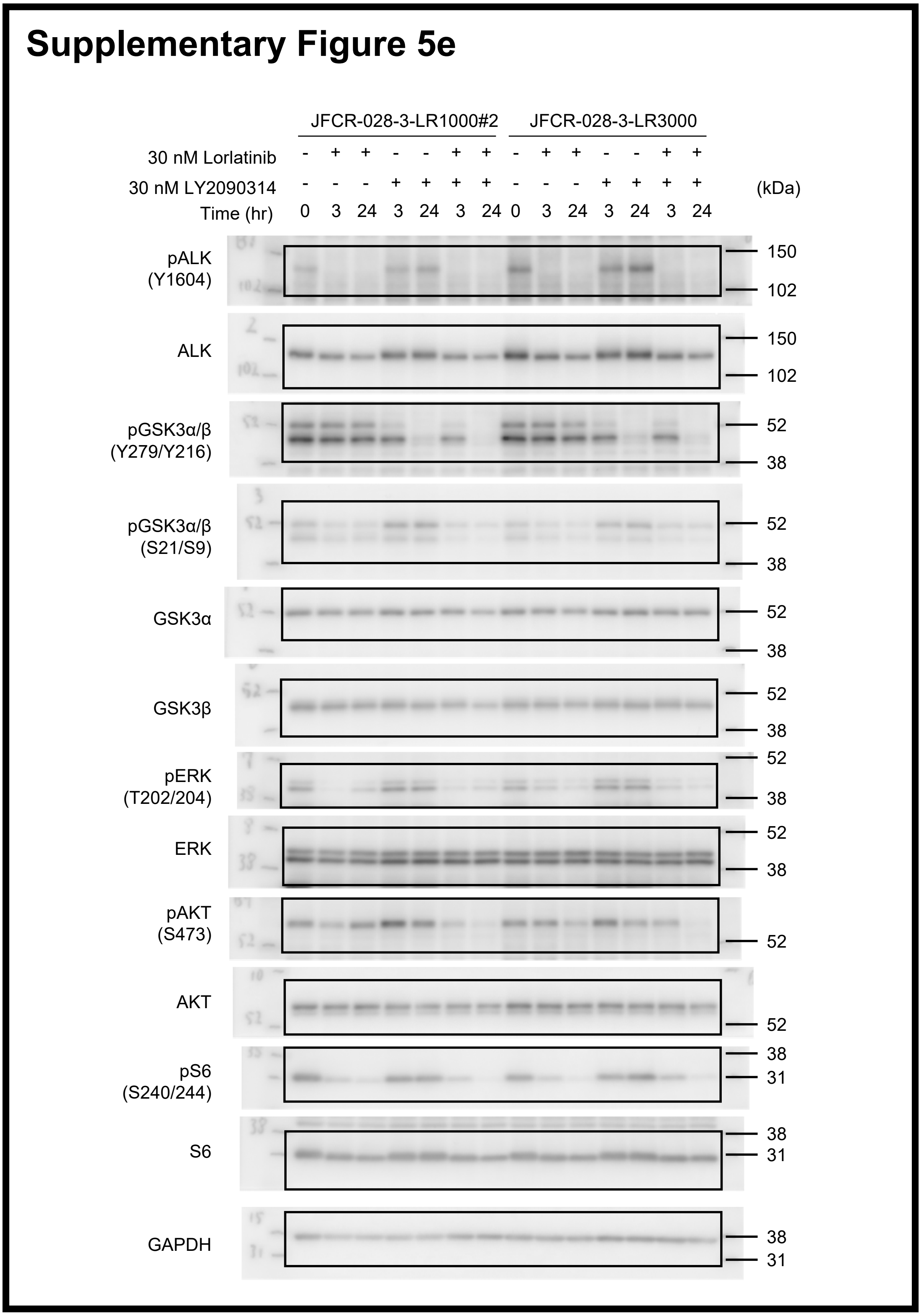

Supplementary Figure 24. Original data of immunoblot analysis for indicated figure.

Supplementary Figure 25

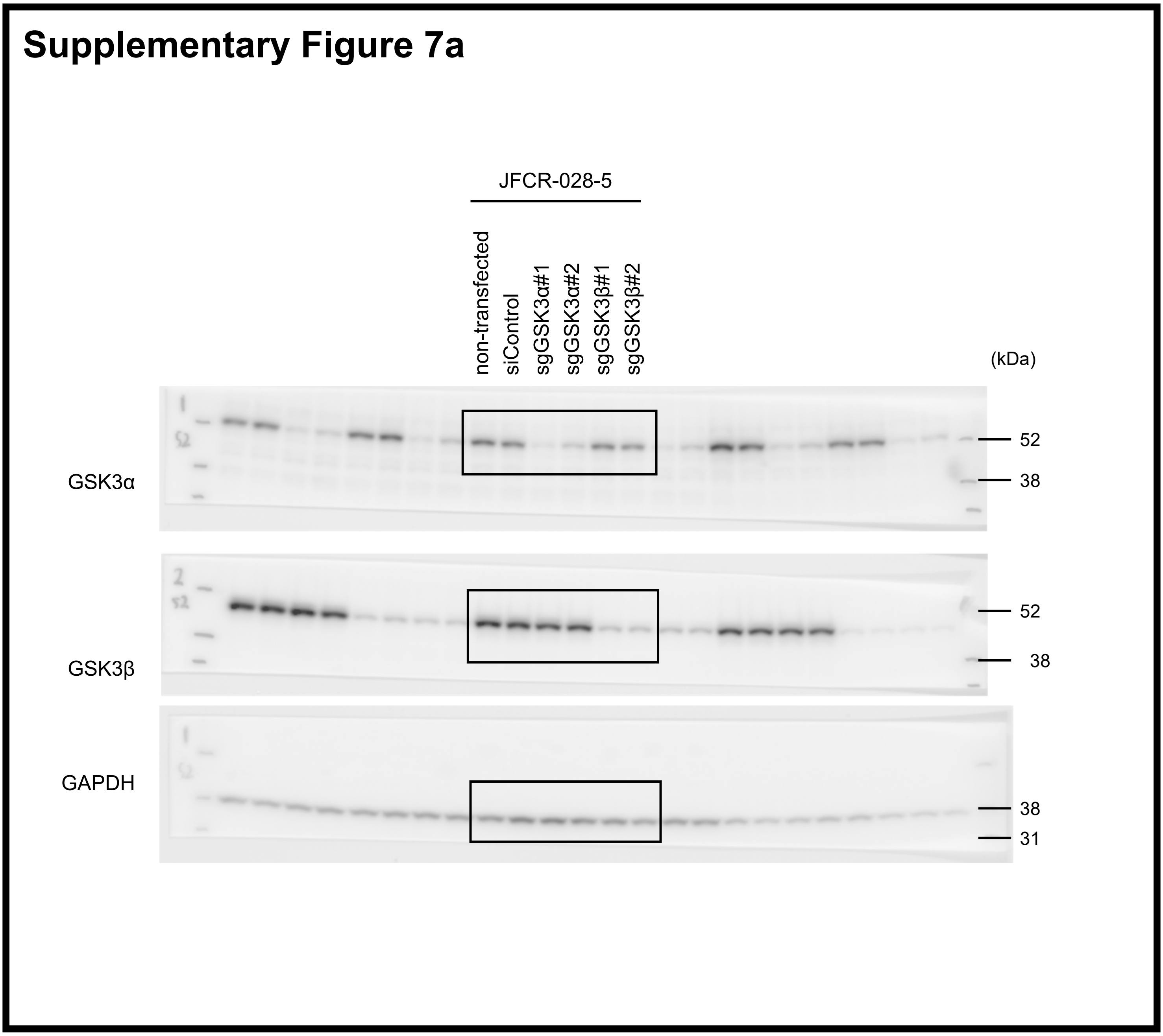

Supplementary Figure 25. Original data of immunoblot analysis for indicated figures.

Supplementary Figure 26

a

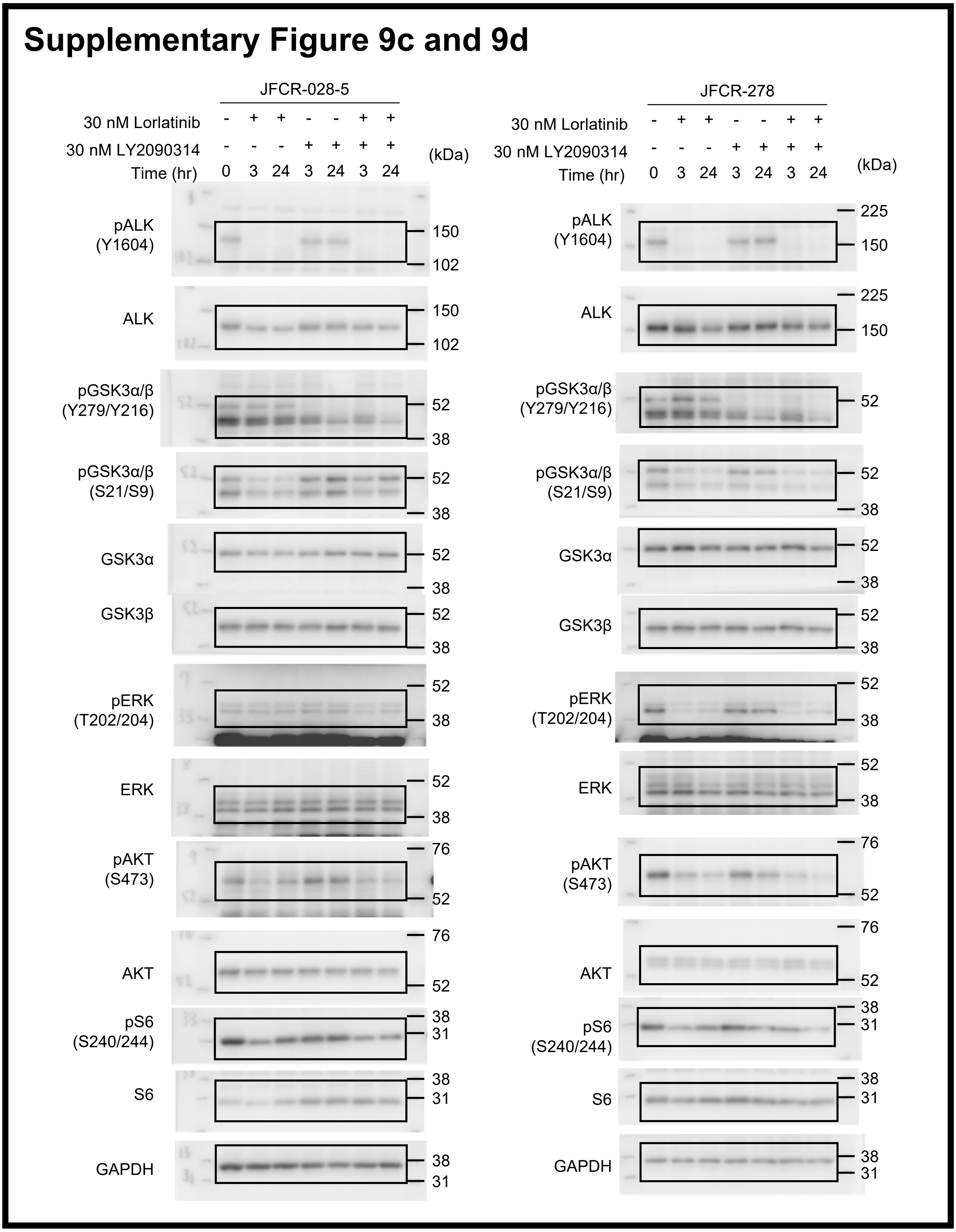

Supplementary Figure 26. Original data of immunoblot analysis for indicated figures.

Supplementary Figure 26

b

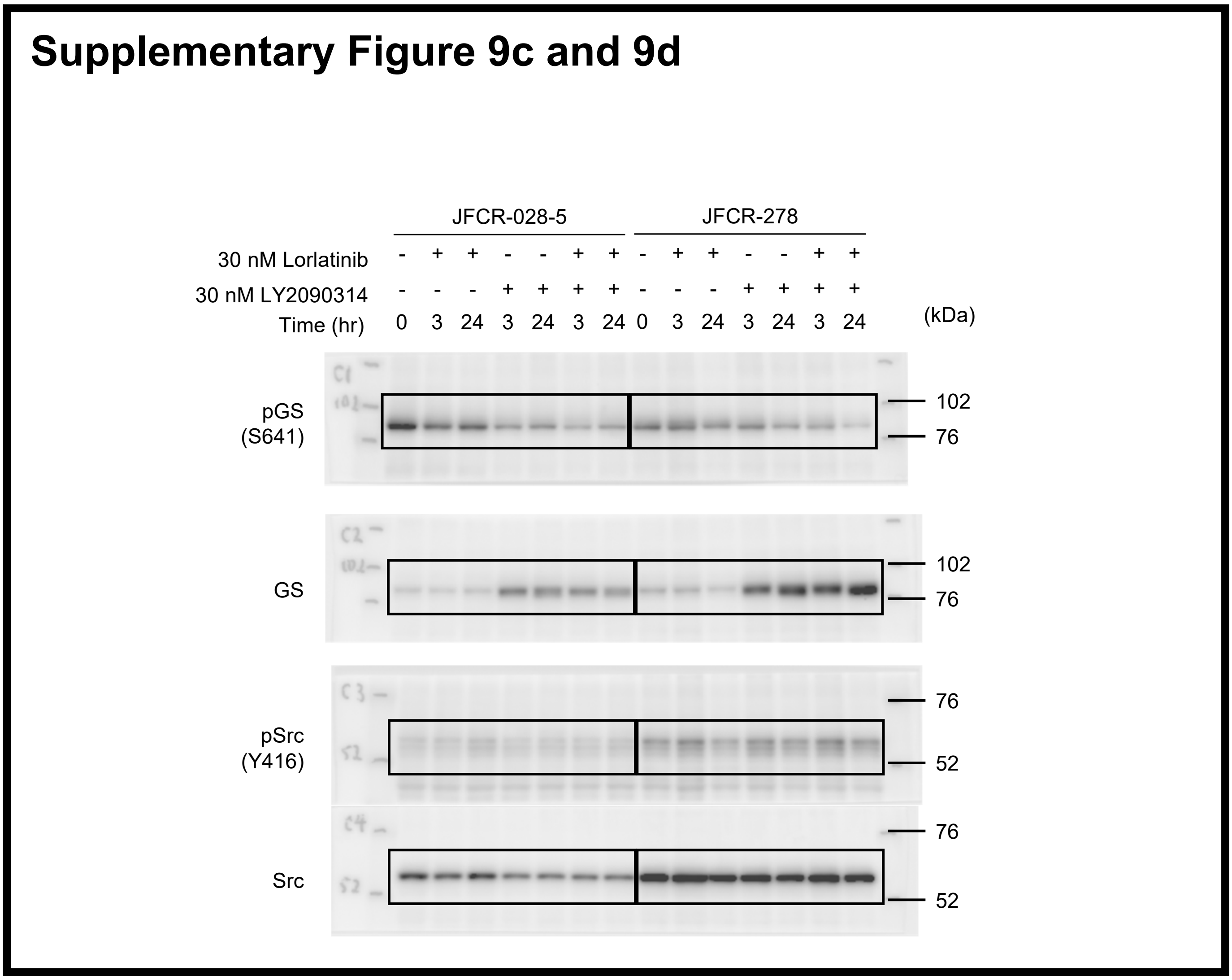

Supplementary Figure 26. Original data of immunoblot analysis for indicated figures.

Supplementary Figure 27

Supplementary Figure 11a

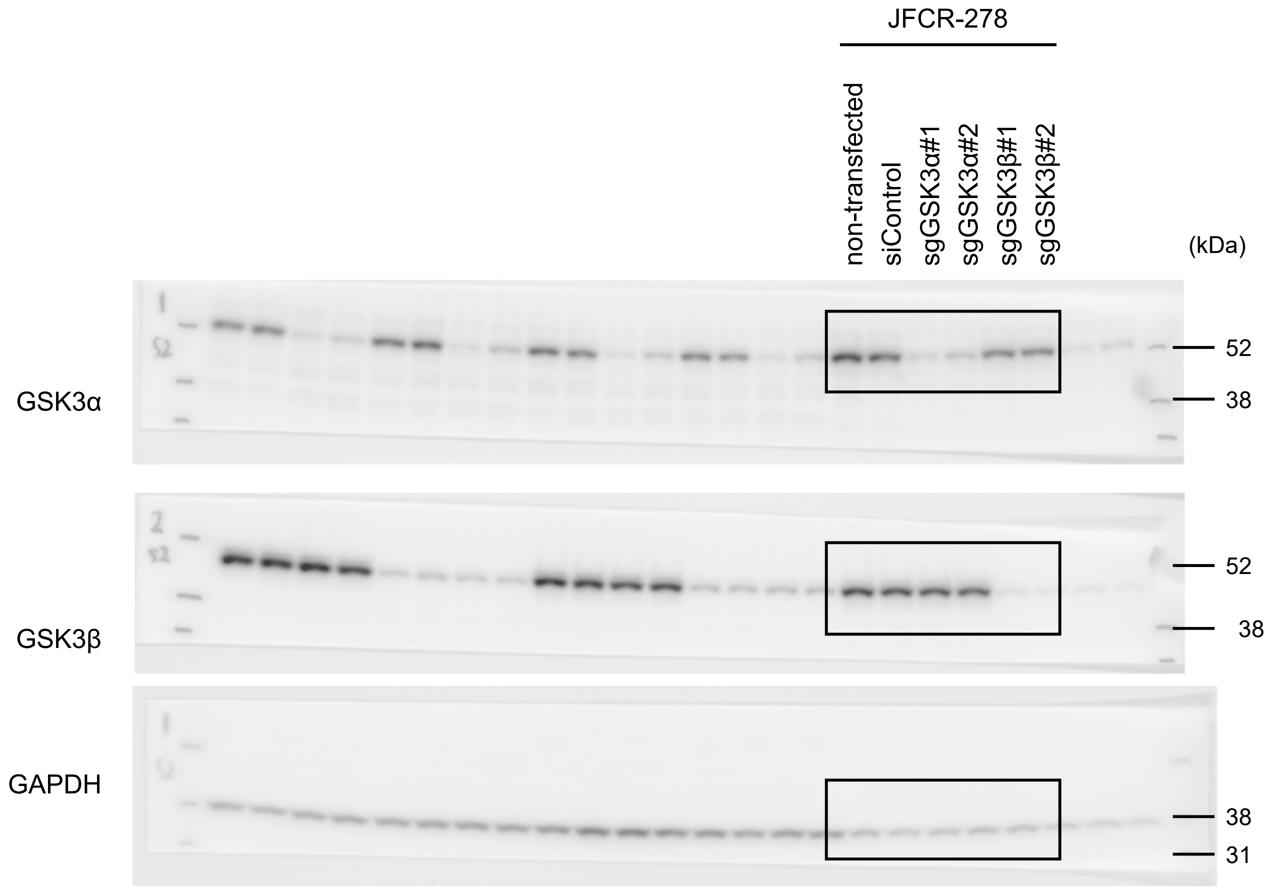

Supplementary Figure 27. Original data of immunoblot analysis for indicated figures.

Supplementary Figure 28

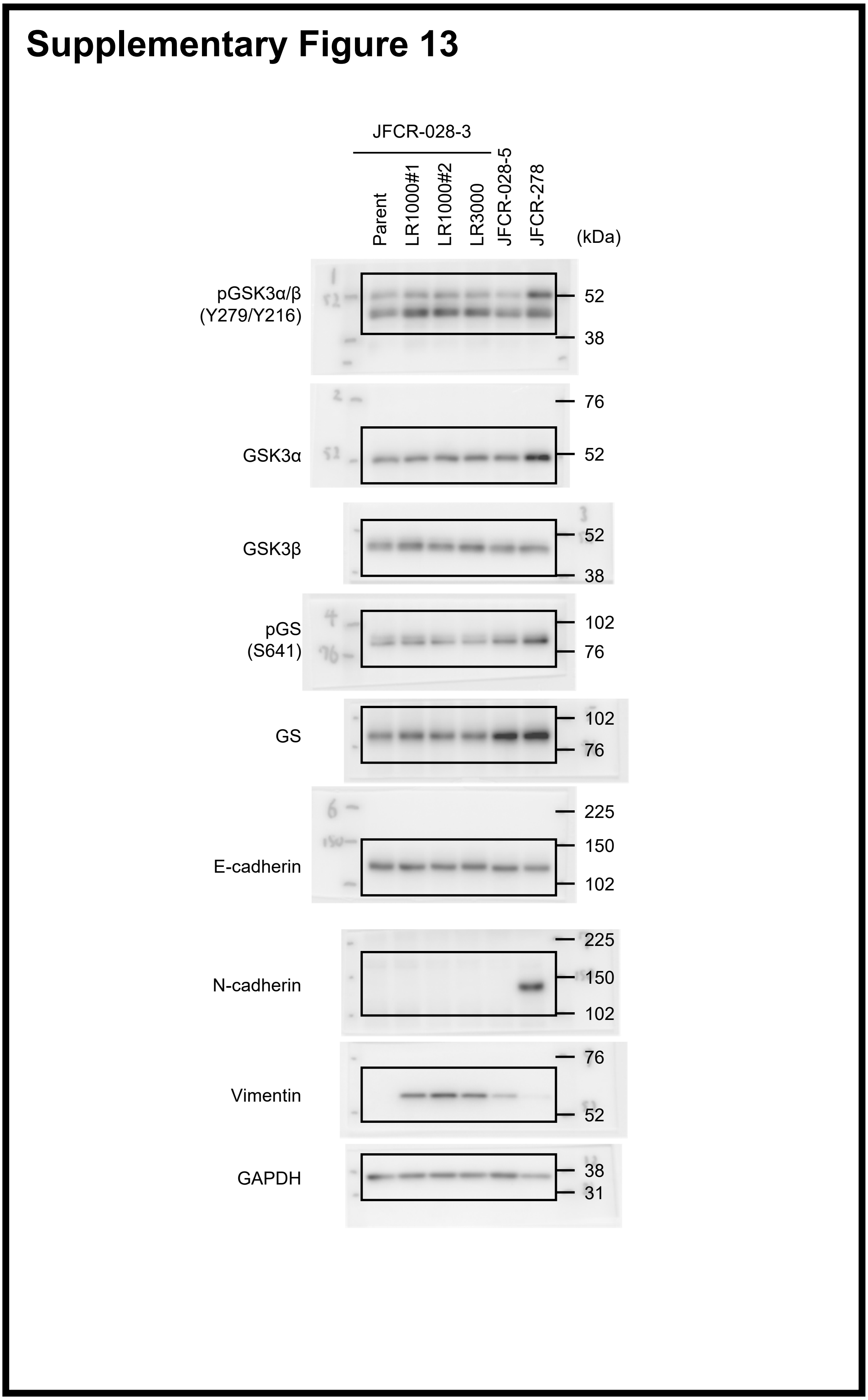

Supplementary Figure 28. Original data of immunoblot analysis for indicated figure.

Supplementary Figure 29

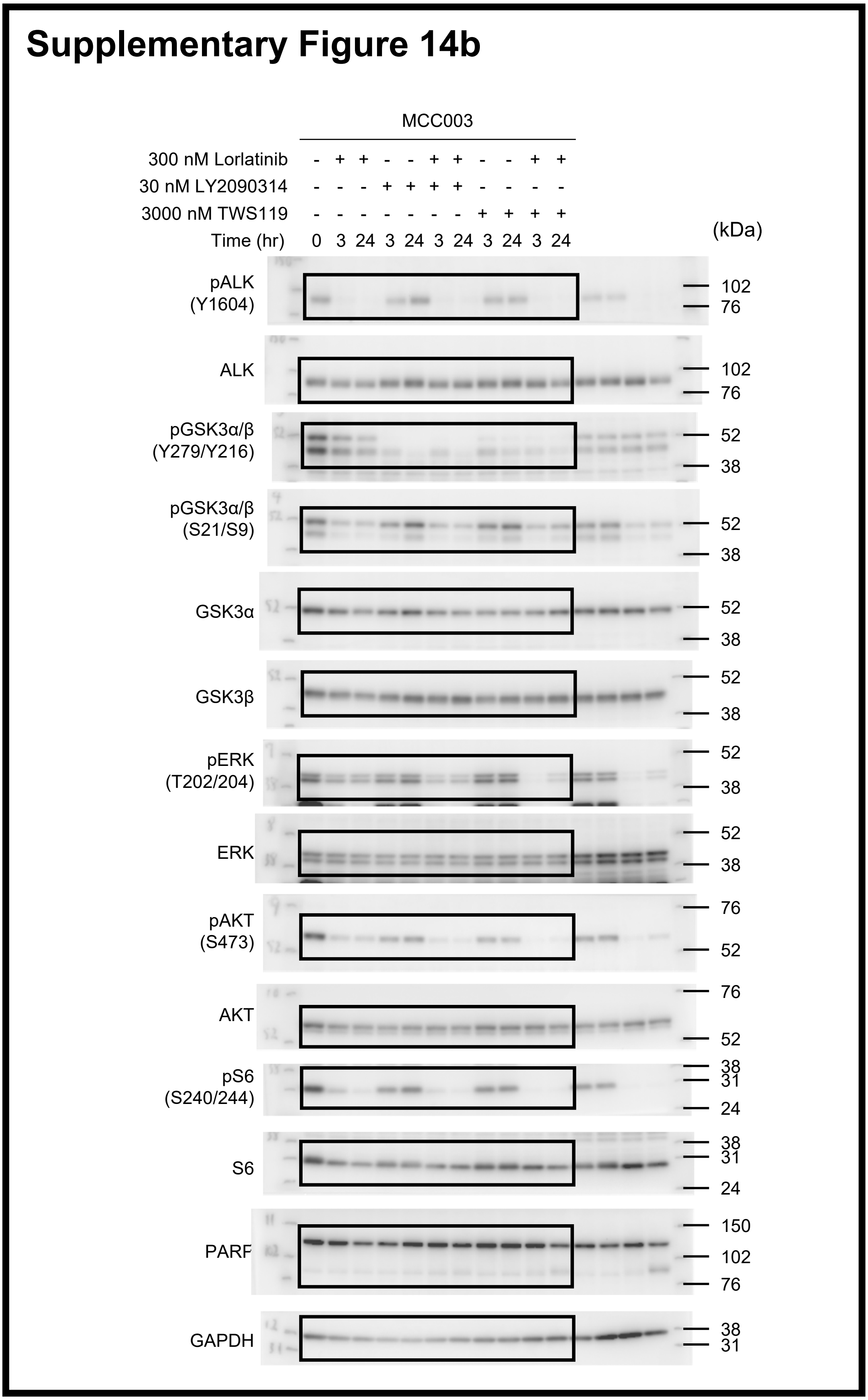

Supplementary Figure 29. Original data of immunoblot analysis for indicated figure.

Supplementary Figure 30

Supplementary Figure 16c

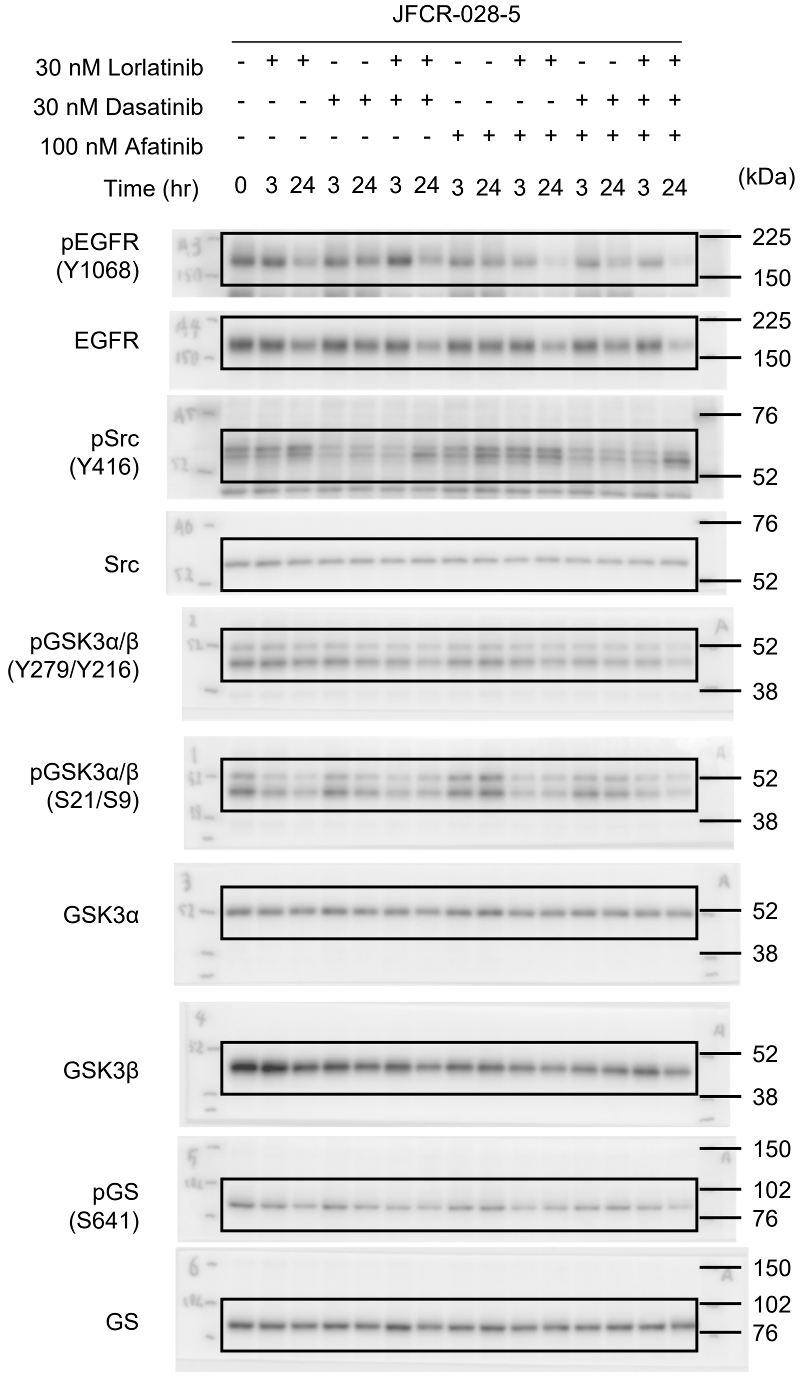

Supplementary Figure 30. Original data of immunoblot analysis for indicated figures.

Supplementary Figure 31

Supplementary Figure 16d

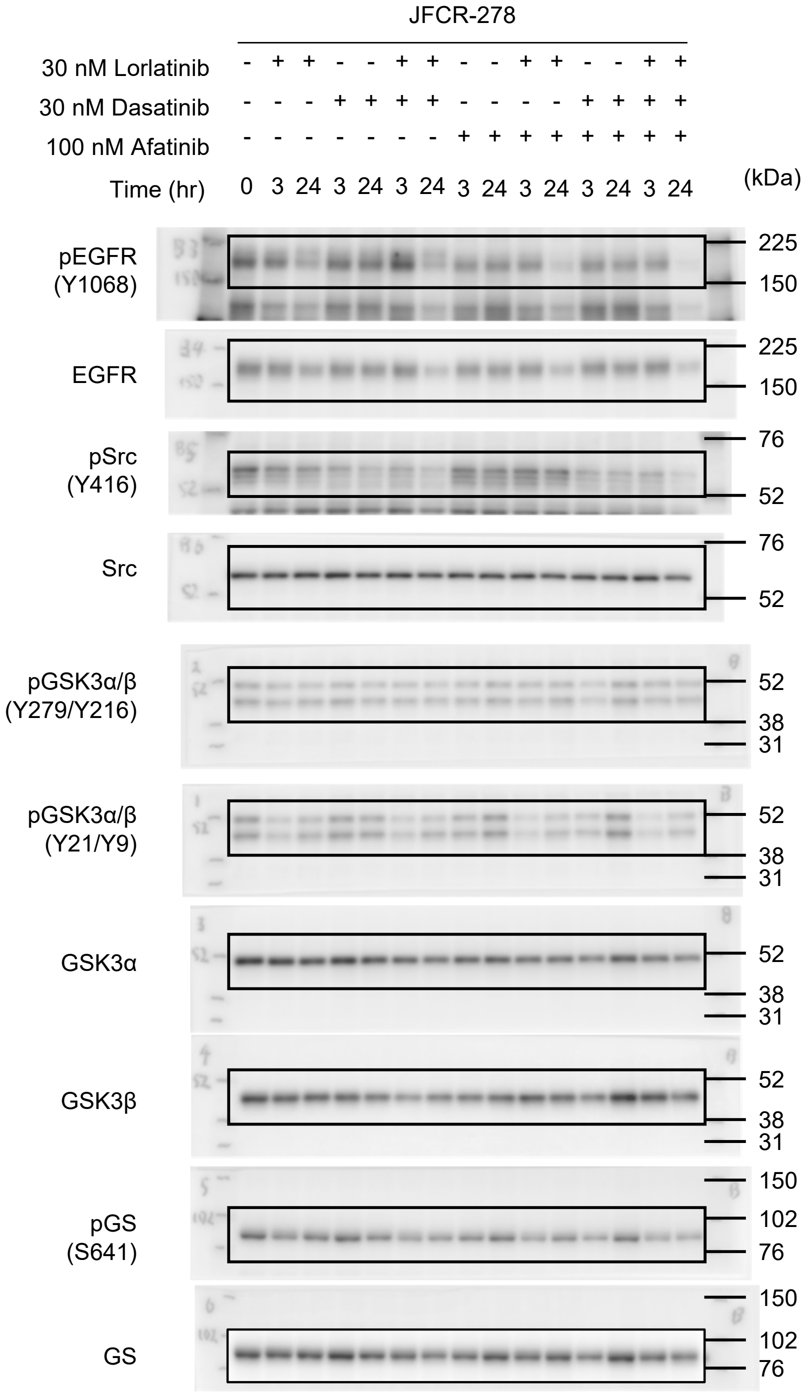

Supplementary Figure 31. Original data of immunoblot analysis for indicated figures.

Supplementary Figure 32

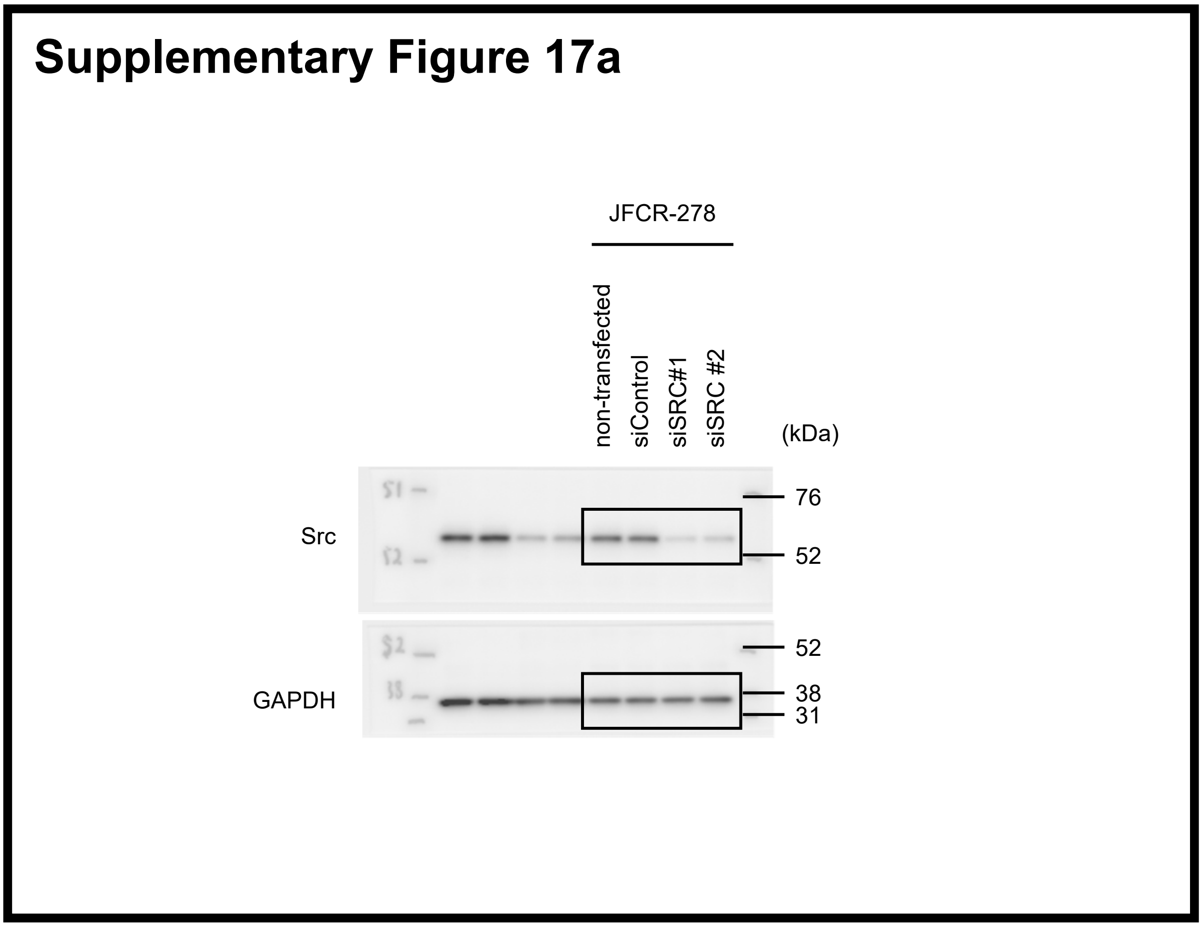

Supplementary Figure 32. Original data of immunoblot analysis for indicated figures.

Supplementary Figure 33

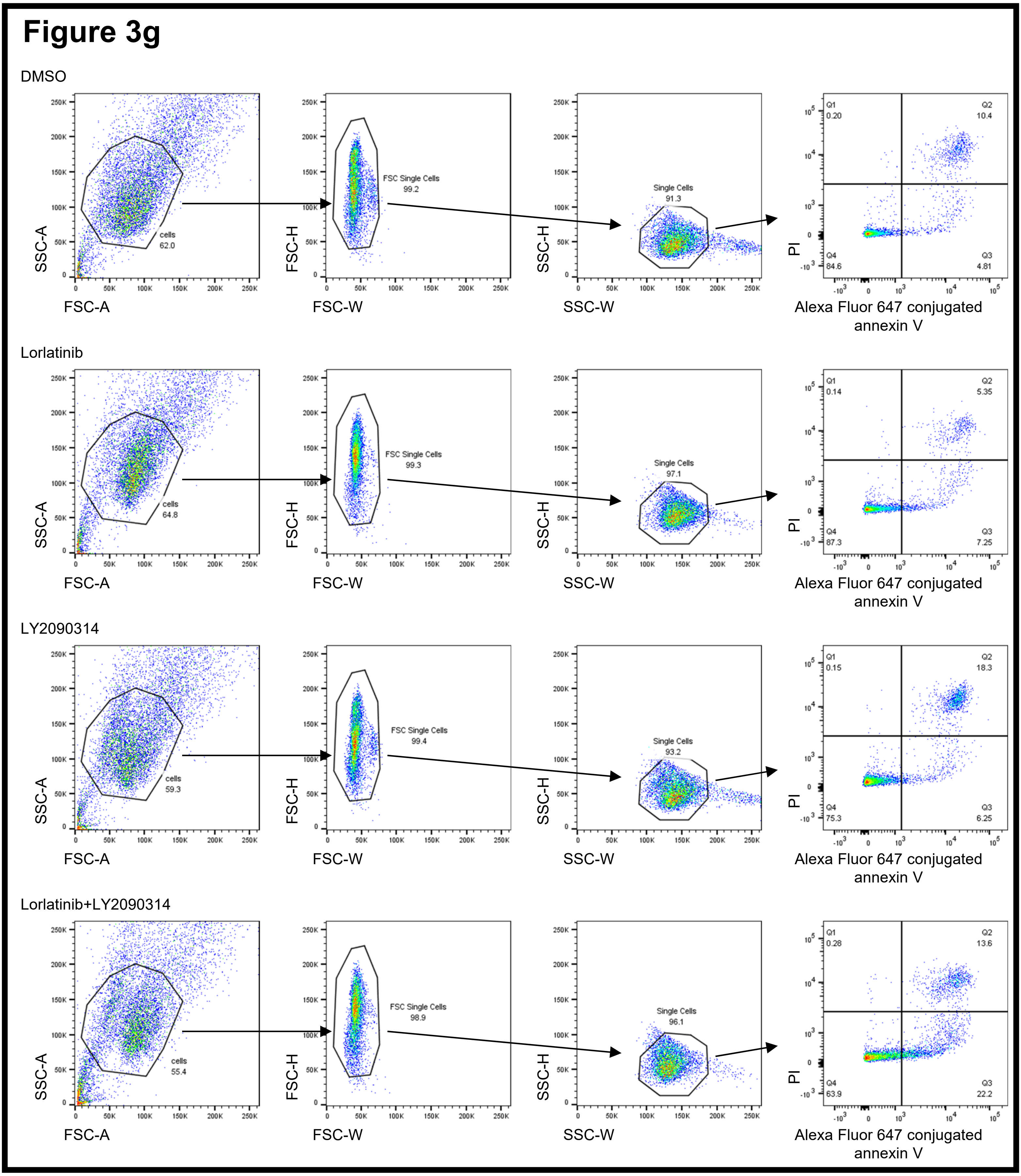

Supplementary Figure 33. Gating strategies for indicated figures of Figure 3g.

Supplementary Figure 34

Figure 6e

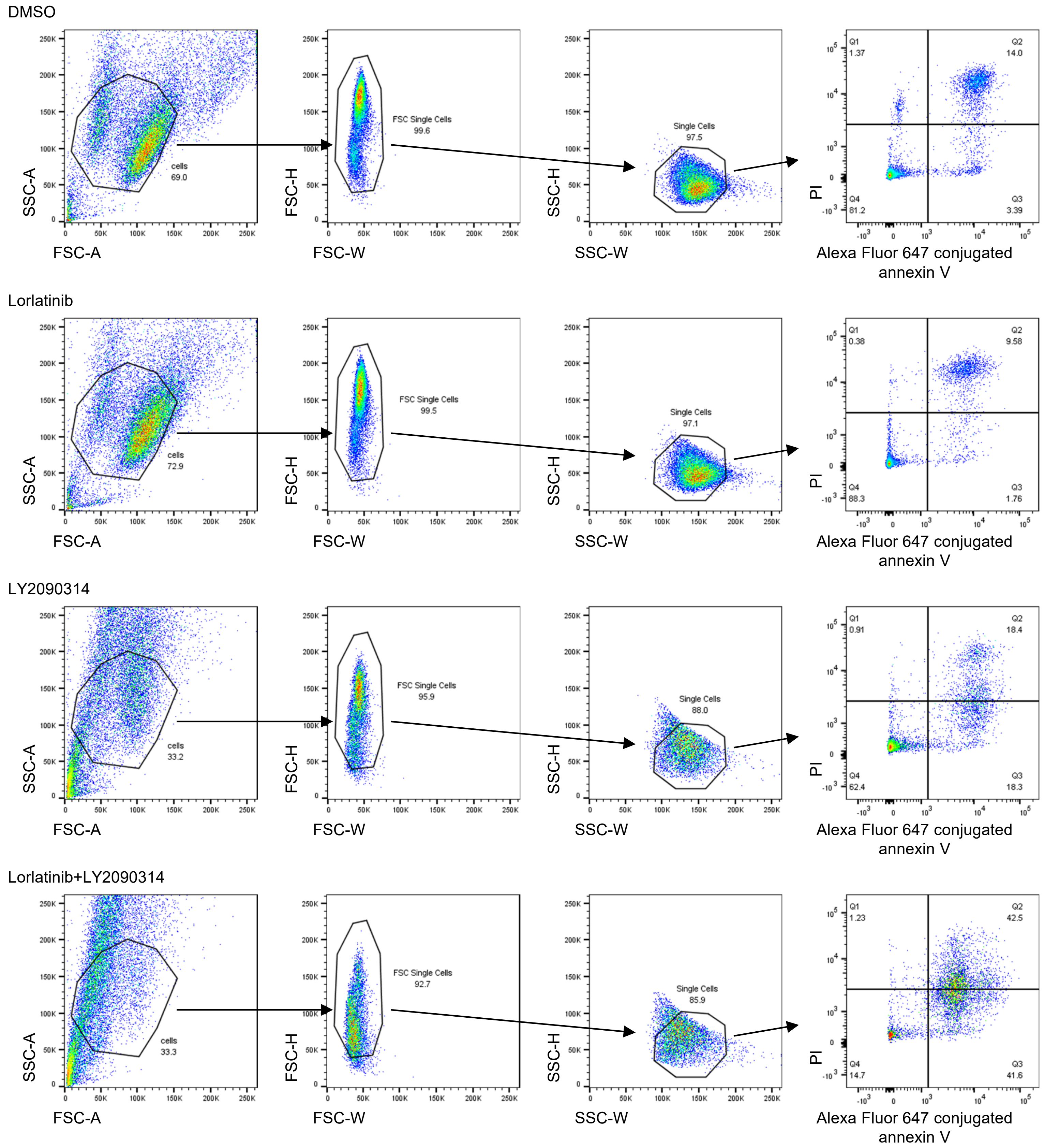

Supplementary Figure 34. Gating strategies for indicated figures of Figure 6e.

Supplementary Figure 35

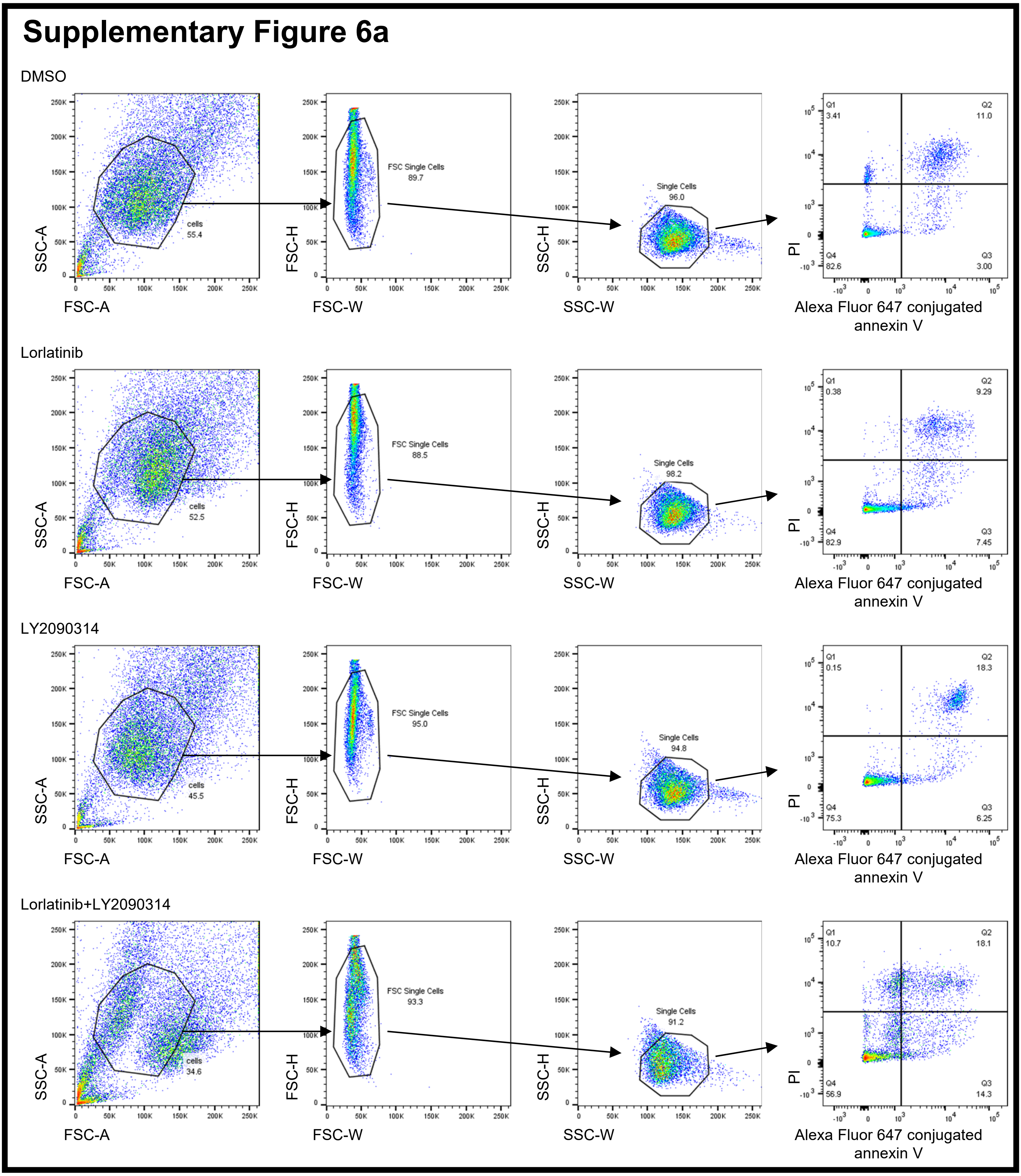

Supplementary Figure 35. Gating strategies for indicated figures of Supplementary Figure 6a.

Supplementary Figure 36

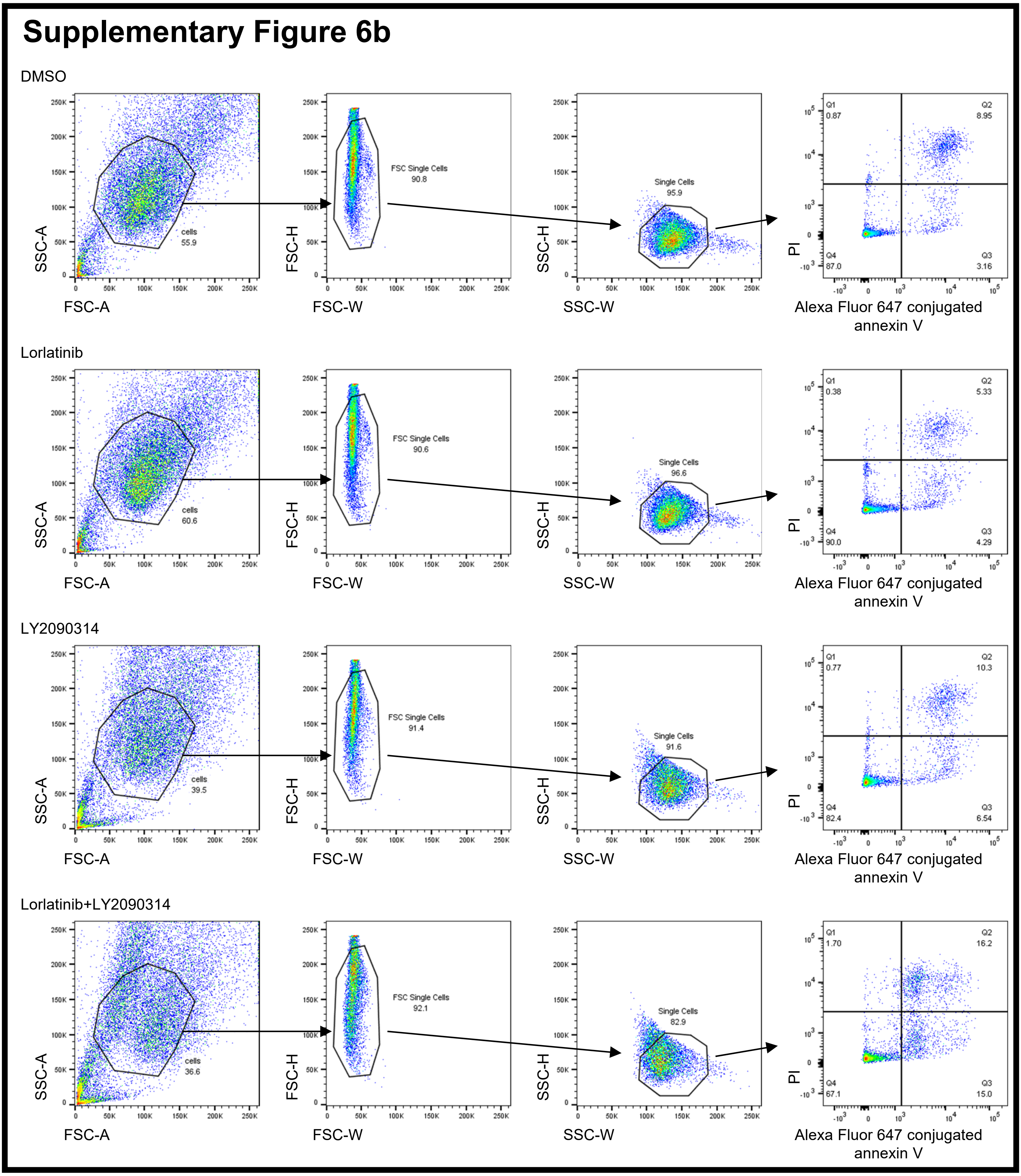

Supplementary Figure 36. Gating strategies for indicated figures of Supplementary Figure 6b.

Supplementary Figure 37

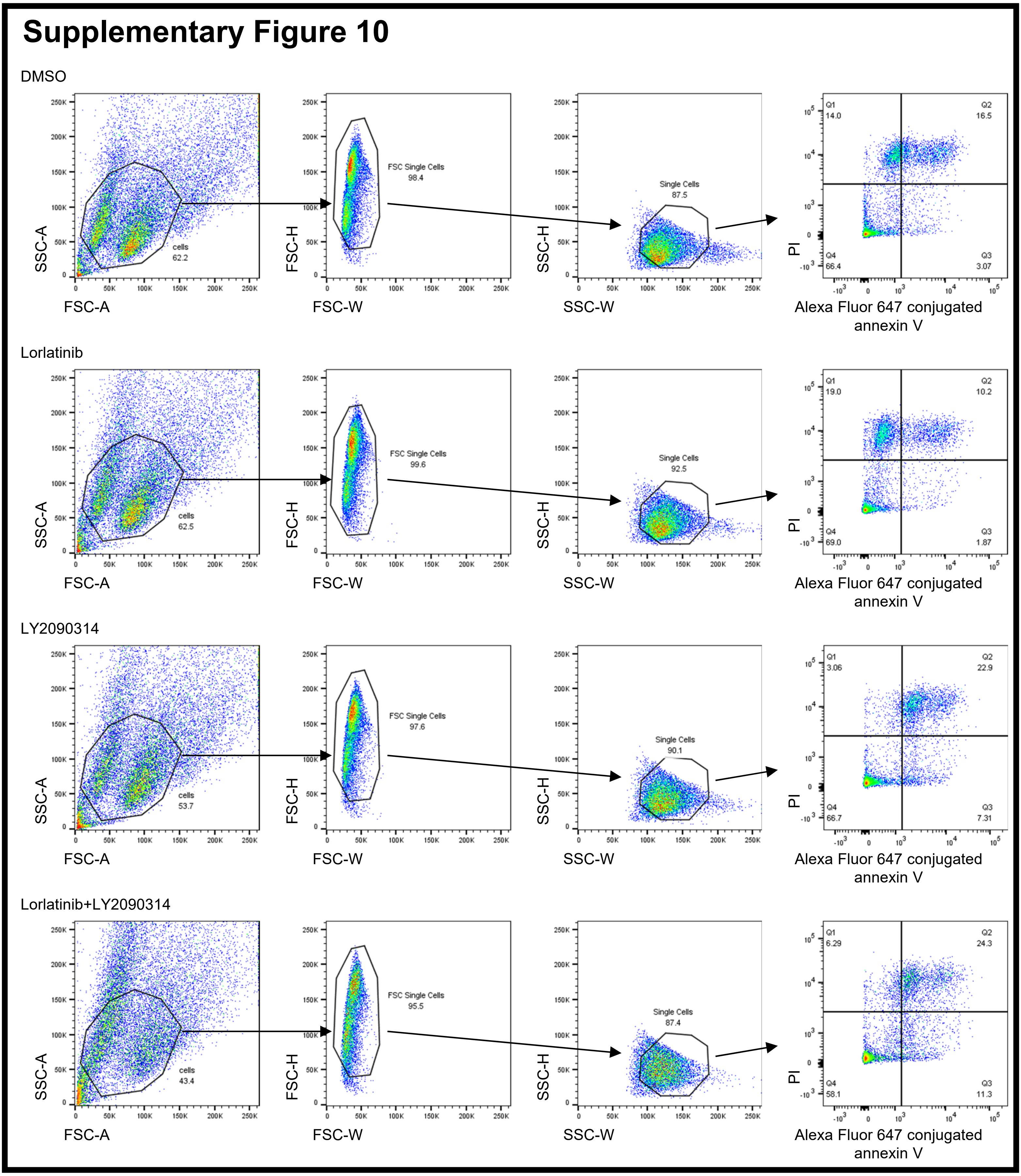

Supplementary Figure 37. Gating strategies for indicated figures of Supplementary Figure 10.

Supplementary Table 1 - The information of the inhibitors using for the focused inhibitor library

drug name	final. conc.	solvent	supplier
Ganetespib	0.1 µM	DMSO	Adooq Bioscience
Dabrafenib	1 µM	DMSO	Adooq Bioscience
BEZ235	1 µM	DMSO	Adooq Bioscience
RO5126766	1 µM	DMSO	Adooq Bioscience
Cobimetinib	0.1 µM	DMSO	Adooq Bioscience
Trametinib	0.1 µM	DMSO	Adooq Bioscience
SCH772984	1 µM	DMSO	Adooq Bioscience
BVD-523	1 µM	DMSO	Adooq Bioscience
GDC0068	1 µM	DMSO	Adooq Bioscience
ABT263	1 µM	DMSO	Adooq Bioscience
Obatoclax	1 µM	DMSO	Adooq Bioscience
ABT199	1 µM	DMSO	Adooq Bioscience
Decitabine	1 µM	DMSO	Adooq Bioscience
Azacitidine	1 µM	DMSO	Adooq Bioscience
Vorinostat	1 µM	DMSO	Adooq Bioscience
Panobinostat	0.1 µM	DMSO	Adooq Bioscience
Quisinostat	0.1 µM	DMSO	ShangHai Biochempartner
Tazemetostat	1 µM	DMSO	Adooq Bioscience
(+)-JQ-1	1 µM	DMSO	ShangHai Biochempartner
Sotrastaurin	1 µM	DMSO	Adooq Bioscience
Nutlin-3	1 µM	DMSO	Adooq Bioscience
RO5045337	1 µM	DMSO	Adooq Bioscience
Ruxolitinib	1 µM	DMSO	Adooq Bioscience
Tofacitinib	1 µM	DMSO	Adooq Bioscience
Palbociclib	1 µM	water	Adooq Bioscience
Ribociclib	1 µM	DMSO	Adooq Bioscience
Alisertib	1 µM	DMSO	Adooq Bioscience
Tozasertib	1 µM	DMSO	Adooq Bioscience
RO4929097	1 µM	DMSO	Adooq Bioscience
LY411575	1 µM	DMSO	Adooq Bioscience
LY2090314	1 µM	DMSO	Adooq Bioscience
Tideglusib	1 µM	DMSO	Adooq Bioscience
Olaparib	1 µM	DMSO	ShangHai Biochempartner
Ibrutinib	1 µM	DMSO	Adooq Bioscience
Erismodegib	1 µM	DMSO	ShangHai Biochempartner
Vismodegib	1 µM	DMSO	Adooq Bioscience
Bortezomib	0.1 µM	DMSO	Adooq Bioscience
Carfilzomib	0.1 µM	DMSO	Adooq Bioscience
Niclosamide	1 µM	DMSO	ShangHai Biochempartner
OSI906	1 µM	DMSO	Adooq Bioscience

drug name	final. conc.	solvent	supplier
5-FU	100 μM	DMSO	Adooq Bioscience
SN-38	0.5 μM	DMSO	Adooq Bioscience
SHP099	5 μM	DMSO	ShangHai Biochempartner
Regorafenib	1 μM	DMSO	Adooq Bioscience
G007-LK	2 μM	DMSO	ShangHai Biochempartner
LY2409881	1 μM	DMSO	Adooq Bioscience
Entrectinib	1 μM	DMSO	Adooq Bioscience
Dovitinib	1 μM	DMSO	Adooq Bioscience
MGCD-265	1 μM	DMSO	Adooq Bioscience
Galunisertib	1 μM	DMSO	Adooq Bioscience
Linifanib	1 μM	DMSO	Adooq Bioscience
AZD3463	1 μM	DMSO	BioVision
AZD5363	1 μM	DMSO	Adooq Bioscience
AUY922	0.1 μM	DMSO	ShangHai Biochempartner
R428	1 μM	DMSO	ShangHai Biochempartner
RXDX105	1 μM	DMSO	ShangHai Biochempartner
Crizotinib	1 μM	DMSO	ShangHai Biochempartner
Ceritinib (LDK378)	1 μM	DMSO	ActiveBiochem
Alectinib	1 μM	DMSO	ActiveBiochem
TAE684	1 μM	DMSO	ChemieTek
AP26113	1 μM	EtOH	ShangHai Biochempartner
Lorlatinib (PF3922)	1 μM	DMSO	ActiveBiochem
ASP3026	1 μM	DMSO	ChemieTek
XL184	1 μM	DMSO	ActiveBiochem
Vandetanib	1 μM	DMSO	ShangHai Biochempartner
E7080	1 μM	DMSO	Selleck
CEP701	1 μM	DMSO	Calbiochem
Foretinib	1 μM	DMSO	Adooq Bioscience
Afatinib (BIBW2992)	0.1 μM	DMSO	ChemieTek
Erlotinib	1 μM	DMSO	LC laboratories
Gefitinib	1 μM	DMSO	LC laboratories
Lapatinib	2 μM	DMSO	LC laboratories
Osimertinib (AZD9291)	1 μM	DMSO	Selleck
PHA665752	1 μM	DMSO	Tocris Bioscience
AEW541	1 μM	DMSO	ActiveBiochem
Sorafenib	1 μM	DMSO	Selleck
Sunitinib	1 μM	DMSO	Selleck
BIBF1120	1 μM	DMSO	Selleck
CH5183284	1 μM	DMSO	ActiveBiochem
BGJ398	1 μM	DMSO	ShangHai Biochempartner
Ponatinib	1 μM	DMSO	Selleck

drug name	final. conc.	solvent	supplier
Imatinib	1 μM	DMSO	LC laboratories
17-AAG	0.1 μM	DMSO	LC laboratories
GDC0941	1 μM	DMSO	LC laboratories
Rapamycin	1 μM	DMSO	AG Scientific
Everolimus	1 μM	DMSO	Chem Scene
PP242	1 μM	DMSO	Adooq Bioscience
SB218078	1 μM	DMSO	Tocris Bioscience
Dasatinib	1 μM	DMSO	Selleck
FH-535	15 μM	DMSO	Adooq Bioscience
Tipifarnib	15 μM	DMSO	Adooq Bioscience
L-OHP	50 μM	DMSO	wako

Supplementary Table 2 - The information of the primers using for RT-qPCR

primer name	sequence
EML4_Forward	TGTTCAAGATCGCCTGTCAGC
EML4_Reverse	TTCACTGAGGCCACATGATC
GAPDH_Forward	ATGGGGAAGGTGAAGGTCG
GAPDH_Reverse	GGGGTCATTGATGGCAACAATA
