# Supplementary material for: GSK3 inhibition circumvents and overcomes acquired lorlatinib resistance in ALK-rearranged non-small-cell lung cancer
Source: NPJ Precis Oncol. 2022 Mar 17;6:16. doi: 10.1038/s41698-022-00260-0 (PMC8931094; doi:10.1038/s41698-022-00260-0)
Supplement: Supplementary file 3 — REPORTING SUMMARY [file 41698_2022_260_MOESM3_ESM.pdf]

nature portfolio

Corresponding author(s): Ryohei Katayama
Last updated by author(s): Feb 2, 2022

Reporting Summary

Nature Portfolio wishes to improve the reproducibility of the work that we publish. This form provides structure for consistency and transparency in reporting. For further information on Nature Portfolio policies, see our Editorial Policies and the Editorial Policy Checklist.

Statistics

For all statistical analyses, confirm that the following items are present in the figure legend, table legend, main text, or Methods section.

n/a	Confirmed	
☐	☒	The exact sample size (*n*) for each experimental group/condition, given as a discrete number and unit of measurement
☐	☒	A statement on whether measurements were taken from distinct samples or whether the same sample was measured repeatedly
☐	☒	The statistical test(s) used AND whether they are one- or two-sided<br>*Only common tests should be described solely by name; describe more complex techniques in the Methods section.*
☒	☐	A description of all covariates tested
☐	☒	A description of any assumptions or corrections, such as tests of normality and adjustment for multiple comparisons
☐	☒	A full description of the statistical parameters including central tendency (e.g. means) or other basic estimates (e.g. regression coefficient) AND variation (e.g. standard deviation) or associated estimates of uncertainty (e.g. confidence intervals)
☐	☒	For null hypothesis testing, the test statistic (e.g. *F*, *t*, *r*) with confidence intervals, effect sizes, degrees of freedom and *P* value noted<br>*Give P values as exact values whenever suitable.*
☒	☐	For Bayesian analysis, information on the choice of priors and Markov chain Monte Carlo settings
☒	☐	For hierarchical and complex designs, identification of the appropriate level for tests and full reporting of outcomes
☒	☐	Estimates of effect sizes (e.g. Cohen's *d*, Pearson's *r*), indicating how they were calculated

Our web collection on statistics for biologists contains articles on many of the points above.

Software and code

Policy information about availability of computer code

Data collection	No software was used.
Data analysis	GraphPad Prism version 9.1.2 and R version 4.0.2 were used to analyze and graphically display the data. FlowJo software was used to analyze and graphically display the data in apoptosis assay. SynergyFinder 2.0 web application tool was used for analyzing synergistic effect of drug combination. ImageJ was used to quantify the band intensity. Information for Proteomics analysis was described in the Supplementary methods and figure legends. QuantaSoft Analysis Pro Software was used for analyzing the expression of genes in droplet digital PCR.

For manuscripts utilizing custom algorithms or software that are central to the research but not yet described in published literature, software must be made available to editors and reviewers. We strongly encourage code deposition in a community repository (e.g. GitHub). See the Nature Portfolio guidelines for submitting code & software for further information.

Data

Policy information about availability of data

All manuscripts must include a data availability statement. This statement should provide the following information, where applicable:
- Accession codes, unique identifiers, or web links for publicly available datasets
- A description of any restrictions on data availability
- For clinical datasets or third party data, please ensure that the statement adheres to our policy

The data that support the findings of this study are available from the corresponding author upon reasonable request.

Field-specific reporting

Please select the one below that is the best fit for your research. If you are not sure, read the appropriate sections before making your selection.

☒ Life sciences ☐ Behavioural & social sciences ☐ Ecological, evolutionary & environmental sciences

For a reference copy of the document with all sections, see nature.com/documents/nr-reporting-summary-flat.pdf

Life sciences study design

All studies must disclose on these points even when the disclosure is negative.

Sample size	Sample size of at least three was used in most experiment for statistical analysis. For the drug screening assay, we performed it by sample size of two.
Data exclusions	No data were excluded.
Replication	We repeated in vitro experiment at least 2 times, and confirmed the reproducibility of the data.
Randomization	No method of randomization was used for in vitro experiments.
Blinding	All experiments were not performed blind due to feasibility.

Reporting for specific materials, systems and methods

We require information from authors about some types of materials, experimental systems and methods used in many studies. Here, indicate whether each material, system or method listed is relevant to your study. If you are not sure if a list item applies to your research, read the appropriate section before selecting a response.

Materials & experimental systems

n/a	Involved in the study	
☐	☒	Antibodies
☐	☒	Eukaryotic cell lines
☒	☐	Palaeontology and archaeology
☒	☐	Animals and other organisms
☒	☐	Human research participants
☒	☐	Clinical data
☒	☐	Dual use research of concern

Methods

n/a	Involved in the study	
☒	☐	ChIP-seq
☐	☒	Flow cytometry
☒	☐	MRI-based neuroimaging

Antibodies

Antibodies used	The following antibodies were purchased from Cell Signaling Technology: total ALK (#3633), phospho-ALK (Y1604, #3341), total AKT (#4691), phospho-AKT (S473, #4060), total p42/44 ERK/MAPK (#9102), phospho-p42/44 ERK/MAPK (T202/Y204, #9101), total GSK3α (#4337), total GSK3β (#12456), phospho-GSK3α/β (S21/S9, #8566), total Glycogen Synthase (GS) (#3893), phospho-Glycogen Synthase (S641, #3891), total Src (#2123), phospho-Src (Y416, #6943), total β-catenin (#8480), phospho-β-catenin (S33/37/T41, # 9561) , total S6 ribosomal protein (#2217), phospho-S6 ribosomal protein (S240/244, #5364), poly(ADP-ribose) polymerase (PARP) (#9542), and cleaved PARP (#9541), E-cadherin (#3195), N-cadherin (#13116), and Vimentin (#5741). In addition, phospho-GSK3α/β (Y279/Y216) antibody was purchased from Abcam, and glyceraldehyde 3-phosphate dehydrogenase (GAPDH) antibody was purchased from Millipore.
Validation	All antibodies are commercially available and have been validated by the companies.

Eukaryotic cell lines

Policy information about cell lines

Cell line source(s)	H3122 cell line was kindly gifted by Dr. Engelman JA. JFCR-018-1, JFCR-028-3, JFCR-028-4, JFCR-028-5, JFCR-093-3, JFCR-198-2, JFCR-278, MCC-003, DU-LAD-002 cells were established from ALK-positive patient-derived NSCLC patients.
Authentication	Public available cell lines were authenticated. H3122 cell line was authenticated by applying short tandem-repeat (STR) DNA profiling analysis. Patient-derived cell lines were confirmed by the sequencing of driver oncogenes.
Mycoplasma contamination	All public cell lines were not detected mycoplasma by the PCR based assay kit. Patient-derived cell lines were not tested for mycoplasma contamination.

Commonly misidentified lines (See ICLAC register)	No commonly misidentified cell lines were not used in this study.

Flow Cytometry

Plots

Confirm that:

☒ The axis labels state the marker and fluorochrome used (e.g. CD4-FITC).

☒ The axis scales are clearly visible. Include numbers along axes only for bottom left plot of group (a 'group' is an analysis of identical markers).

☒ All plots are contour plots with outliers or pseudocolor plots.

☒ A numerical value for number of cells or percentage (with statistics) is provided.

Methodology

Sample preparation	Cells were seeded at a density of 1 × 105 cells/well in 6-well plates. After overnight culture, cells were treated with the indicated concentration of drugs. All floating and adherent cells were collected after 72 h of drug treatment. Cells were stained with propidium iodide and Alexa Fluor 647 conjugated annexin V using a Annexin V / Dead Cell Apoptosis Kit (Thermo Fischer Scientific) for 15 min at room temperature.
Instrument	FACS Lyric
Software	FlowJo
Cell population abundance	More than 10,000 cells were counted for the apoptosis assay.
Gating strategy	We used only FSC and SSC for gating strategy.

☒ Tick this box to confirm that a figure exemplifying the gating strategy is provided in the Supplementary Information.
